# Supplementary material for: Using routine healthcare data to evaluate the impact of the Medicines at Transitions Intervention (MaTI) on clinical outcomes of patients hospitalised with heart failure: protocol for the Improving the Safety and Continuity Of Medicines management at Transitions of care (ISCOMAT) cluster randomised controlled trial with embedded process evaluation, health economics evaluation and internal pilot
Source: BMJ Open. 2022 Apr 29;12(4):e054274. doi: 10.1136/bmjopen-2021-054274 (PMC9058770; doi:10.1136/bmjopen-2021-054274)
Supplement: Supplementary data [file bmjopen-2021-054274supp001.pdf]

MaTI Form A

Checklist for MaTI patients

Keep this checklist in the MaTI box file in the agreed place on the ward

Patient name: ______________________________

DoB: ____________________ NHS number: ____________________

MaTI Form A

Checklist for MaTI patients

FOR STUDY USE ONLY

INITIALS ____ DOB ____ TRIAL No. ____

SITE NAME ____ SITE No. ____

Step	Description and tool used	When?	Date completed	Who? (role, initials)	Unable to complete (reason)
1	MaTI checklist in patient's notes	During admission			
2a	Identify community pharmacist (Pharmacy form to include in notes)	During admission/ medicines reconciliation			
2b	Contact the pharmacist to let them about the patient and discharge information				
3	Patient introduced to the My Medicines Toolkit booklet (MaTI booklet)	Before the day of discharge			
4	Discharge heart failure medicines log completed (foldout in MaTI toolkit)	On day of discharge			
5	Patient informed about being referred to their community pharmacy for a follow up and a medicines use review / discussion about medicines	On day of discharge			
6	Transfer the patient's discharge advice letter and medicines list to the community pharmacy (MaTI community pharmacy cover letter)	At / soon after discharge			
7	Information has been received by the community pharmacist? (by telephone)	At / soon after discharge	Y/ N		
