# Supplementary material for: Using routine healthcare data to evaluate the impact of the Medicines at Transitions Intervention (MaTI) on clinical outcomes of patients hospitalised with heart failure: protocol for the Improving the Safety and Continuity Of Medicines management at Transitions of care (ISCOMAT) cluster randomised controlled trial with embedded process evaluation, health economics evaluation and internal pilot
Source: BMJ Open. 2022 Apr 29;12(4):e054274. doi: 10.1136/bmjopen-2021-054274 (PMC9058770; doi:10.1136/bmjopen-2021-054274)
Supplement: Supplementary data [file bmjopen-2021-054274supp002.pdf]

MaTI Form B

Community pharmacy record

Date: ______________________________

Patient name: ______________________________

DoB: ______________________________

MaTI Form B

Community pharmacy record

FOR STUDY USE ONLY	INITIALS		DOB		TRIAL No.	
	SITE NAME				SITE No.	

Date: ______________________________

Community pharmacy name: ______________________________

Community pharmacy address: ______________________________

____

____

____

Phone: ______________________________

Fax: ______________________________

Secure NHS email: ______________________________
