# Supplementary material for: Using routine healthcare data to evaluate the impact of the Medicines at Transitions Intervention (MaTI) on clinical outcomes of patients hospitalised with heart failure: protocol for the Improving the Safety and Continuity Of Medicines management at Transitions of care (ISCOMAT) cluster randomised controlled trial with embedded process evaluation, health economics evaluation and internal pilot
Source: BMJ Open. 2022 Apr 29;12(4):e054274. doi: 10.1136/bmjopen-2021-054274 (PMC9058770; doi:10.1136/bmjopen-2021-054274)
Supplement: Supplementary data [file bmjopen-2021-054274supp003.pdf]

Community Pharmacy Cover Letter

Ward Contact Details Name ________________________ Telephone ________________________

Handover of patient medicines care to community pharmacy

Patient name: ____

D.O.B: ____

To the Pharmacist in charge

A patient has now been discharged from hospital and action is required by you

What is this about?

We are writing to you because we have introduced improved discharge communication between our hospital and community pharmacy for patients with heart failure. It is part of a study called ISCOMAT/MaTI. For more information please see **https://www.brad.ac.uk/life-sciences/pharmacy-medical-sciences/morg/iscomat/**

What do I need to do?

We have attached a copy of the patient's discharge advice note / letter and this includes a copy of the patient's discharge medicines list. You now need to:

- Reconcile the patient's medicines (resolve any discrepancies between the medicines in the discharge list and the patient's next repeat prescription).
- Invite the patient for a post-discharge Medicines Use Review (MUR), or a discussion about their medicines (to answer any questions they may have about their medicines, to resolve any discrepancies and to provide support as necessary).
- Complete the checklist included and keep it with your records.

If the patient wishes their MUR to take place by telephone you can apply for permission to do this using the form on the PSNC website:
http://psnc.org.uk/services-commissioning/advanced-services/murs/conducting-murs-off-the-pharmacy-premises/

Free online CPD for you and your staff

If you and/or a member of your team would like to you can complete a short e-learning CPD module for staff involved with patients' medicines - to help them find out more about the risks that patients face when their care is transferred between providers. This training, which will take approximately one hour, is available free of charge to you and your team. We hope you will encourage your staff to undertake it. It has been developed by the Centre for Pharmacy Postgraduate Education (CPPE). It can be accessed here:
www.cppe.ac.uk/canvas/iscomat

ISCOMAT is a national study and has Health Research Authority (HRA) and Research Ethics Committee approval.

If you require any further information about the patient, please contact staff on the ward using the contact details at the top of this letter.
