# Supplementary material for: Using routine healthcare data to evaluate the impact of the Medicines at Transitions Intervention (MaTI) on clinical outcomes of patients hospitalised with heart failure: protocol for the Improving the Safety and Continuity Of Medicines management at Transitions of care (ISCOMAT) cluster randomised controlled trial with embedded process evaluation, health economics evaluation and internal pilot
Source: BMJ Open. 2022 Apr 29;12(4):e054274. doi: 10.1136/bmjopen-2021-054274 (PMC9058770; doi:10.1136/bmjopen-2021-054274)
Supplement: Author's manuscript [file bmjopen-2021-054274.draft_revisions.pdf]

BMJ Open is committed to open peer review. As part of this commitment we make the peer review history of every article we publish publicly available.

When an article is published we post the peer reviewers' comments and the authors' responses online. We also post the versions of the paper that were used during peer review. These are the versions that the peer review comments apply to.

The versions of the paper that follow are the versions that were submitted during the peer review process. They are not the versions of record or the final published versions. They should not be cited or distributed as the published version of this manuscript.

BMJ Open is an open access journal and the full, final, typeset and author-corrected version of record of the manuscript is available on our site with no access controls, subscription charges or pay-per-view fees (http://bmjopen.bmj.com).

If you have any questions on BMJ Open's open peer review process please email info.bmjopen@bmj.com

BMJ Open

# Using Routine Healthcare Data to evaluate the impact of the Medicines at Transitions Intervention (MaTI) on clinical outcomes of patients hospitalised with heart failure: protocol for the Improving the Safety and Continuity Of Medicines management at Transitions of care (ISCOMAT) cluster randomised controlled trial with embedded process evaluation, health economics evaluation and internal pilot

| | |
|---|---|
| Journal: | *BMJ Open* |
| Manuscript ID | bmjopen-2021-054274 |
| Article Type: | Protocol |
| Date Submitted by the Author: | 10-Jun-2021 |
| Complete List of Authors: | Moreau, Lauren; University of Leeds Clinical Trials Research Unit, CTRU<br>Holloway, Ivana; University of Leeds Clinical Trials Research Unit<br>Fylan, Beth; University of Bradford, School of Pharmacy and Medical Sciences; NIHR Yorkshire and Humber PSTRC, Bradford Insitiue for Health Research<br>Hartley, Suzanne; University of Leeds Clinical Trials Research Unit, Clinical Trials Research Unit<br>Cundill, Bonnie; University of Leeds Clinical Trials Research Unit<br>Fergusson, Alison; University of Leeds Clinical Trials Research Unit<br>Alderson, Sarah; University of Leeds, Academic Unit of Primary Care<br>Alldred, David; University of Leeds, School of Healthcare; NIHR Yorkshire and Humber Patient Safety Translational Research Centre, Bradford Institute for Health Research<br>Bojke, Chris; University of Leeds, Academic Unit of Health Economics<br>Breen, Liz; University of Bradford, School of Pharmacy and Medical Sciences; University of Bradford<br>Ismail, Hanif; University of Bradford, School of Pharmacy and Medical Sciences<br>Gardner, Peter; University of Bradford, School of Pharmacy and Medical Sciences; University of Bradford, Wolfson Centre for Applied Health Research<br>Mason, Ellen; University of Leeds Clinical Trials Research Unit<br>Powell, Catherine; University of Bradford Faculty of Health Studies, Centre for Applied Dementia Studies<br>Silcock, Jonathan; University of Bradford School of Pharmacy and Medical Sciences<br>Taylor, Andrew; ISCOMAT Patient Led Steering Group<br>Farrin, Amanda; University of Leeds Clinical Trials Research Unit<br>Gale, Chris; University of Leeds Leeds Institute of Cardiovascular and Metabolic Medicine; Leeds Teaching Hospitals NHS Trust Department of Cardiology |
| <b>Primary Subject Heading</b>: | |

| | |
|---|---|
| Secondary Subject Heading: | |
| Keywords: | Heart failure < CARDIOLOGY, Cardiology < INTERNAL MEDICINE, Clinical trials < THERAPEUTICS |
| | |


SCHOLARONE™ Manuscripts

## Title

Using Routine Healthcare Data to evaluate the impact of the Medicines at Transitions Intervention (MaTI) on clinical outcomes of patients hospitalised with heart failure: protocol for the Improving the Safety and Continuity Of Medicines management at Transitions of care (ISCOMAT) cluster randomised controlled trial with embedded process evaluation, health economics evaluation and internal pilot

## Names of protocol contributors

Lauren Moreau[1], Ivana Holloway[1], Beth Fylan[2], Suzanne Hartley[1], Bonnie Cundill[1], Alison Fergusson[1], Sarah Alderson[3], David Alldred[2, 3], Chris Bojke, Liz Breen[2], Hanif Ismail[2], Peter Gardner[2], Ellen Mason[1], Catherine Powell[2], Jon Silcock[2], Andrew Taylor[5], Amanda J. Farrin[1], Chris P. Gale[6]. On behalf of the ISCOMAT Programme Management Team

[1]Leeds Institute for Clinical Trials Research, Clinical Trials Research Unit, University of Leeds, Leeds, UK LS2 9JT

[2]School of Pharmacy and Medical Sciences, Faculty of Life Sciences, University of Bradford, Bradford, UK; Wolfson Centre for Applied Health Research, Bradford, UK

NIHR Yorkshire and Humber Patient Safety Translational Research Centre, Bradford, UK

[3]School of Healthcare, University of Leeds, Leeds, UK

[4]Academic Unit for Health Economics, University of Leeds, Leeds UK LS2 9JT

[5] ISCOMAT Patient Led Steering Group, Manchester UK

[6]Leeds Institute of Cardiovascular and Metabolic Medicine, University of Leeds, Leeds, UK; Leeds Institute for Data Analytics, University of Leeds, Leeds, UK; Department of Cardiology, Leeds Teaching Hospitals NHS Trust, Leeds, UK

Lauren Moreau (corresponding author), Leeds Institute for Clinical Trials Research, Clinical Trials Research Unit, University of Leeds, Leeds, UK LS2 9JT; 0113 343 7588; l.a.moreau@leeds.ac.uk

Ivana Holloway, Leeds Institute for Clinical Trials Research, Clinical Trials Research Unit, University of Leeds, Leeds, UK LS2 9JT; I.Holloway@leeds.ac.uk

Beth Fylan, School of Pharmacy and Medical Sciences, Faculty of Life Sciences, University of Bradford, Bradford, UK; Wolfson Centre for Applied Health Research, Bradford, UK; B.Fylan@bradford.ac.uk

Suzanne Hartley, Leeds Institute for Clinical Trials Research, Clinical Trials Research Unit, University of Leeds, Leeds, UK LS2 9JT; S.Hartley@leeds.ac.uk

Bonnie Cundill, Leeds Institute for Clinical Trials Research, Clinical Trials Research Unit, University of Leeds, Leeds, UK LS2 9JT; B.E.Cundill@leeds.ac.uk


For peer review only - http://bmjopen.bmj.com/site/about/guidelines.xhtml

Alison Fergusson, Leeds Institute for Clinical Trials Research, Clinical Trials Research Unit, University of Leeds, Leeds, UK LS2 9JT; A.Fergusson@leeds.ac.uk

Sarah Alderson, Leeds Institute for Health Sciences, University of Leeds, Leeds, UK LS2 9JT; S.L.Alderson@leeds.ac.uk

David Alldred, Wolfson Centre for Applied Health Research, Bradford, UK; Bradford Institute for Health Research, NIHR Yorkshire and Humber Patient Safety Translational Research Centre, Bradford, UK; School of Healthcare, University of Leeds, Leeds, UK; D.P.Alldred@leeds.ac.uk

Chris Bojke, Academic Unit for Health Economics, University of Leeds, Leeds UK LS2 9JT; C.Bojke@leeds.ac.uk

Liz Breen School of Pharmacy and Medical Sciences, Faculty of Life Sciences, University of Bradford, Bradford, UK; Wolfson Centre for Applied Health Research, Bradford, UK; Bradford Institute for Health Research, NIHR Yorkshire and Humber Patient Safety Translational Research Centre, Bradford, UK; L.Breen@bradford.ac.uk

Hanif Ismail School of Pharmacy and Medical Sciences, Faculty of Life Sciences, University of Bradford, Bradford, UK; Wolfson Centre for Applied Health Research, Bradford, UK; H.Ismail11@bradford.ac.uk

Peter Gardner School of Pharmacy and Medical Sciences, Faculty of Life Sciences, University of Bradford, Bradford, UK; Wolfson Centre for Applied Health Research, Bradford, UK; P.H.Gardner@bradford.ac.uk

Ellen Mason, Leeds Institute for Clinical Trials Research, Clinical Trials Research Unit, University of Leeds, Leeds, UK LS2 9JT; E.Mason1@leeds.ac.uk

Catherine Powell School of Pharmacy and Medical Sciences, Faculty of Life Sciences, University of Bradford, Bradford, UK; Wolfson Centre for Applied Health Research, Bradford, UK; C.Powell2@bradford.ac.uk

Jon Silcock School of Pharmacy and Medical Sciences, Faculty of Life Sciences, University of Bradford, Bradford, UK; Wolfson Centre for Applied Health Research, Bradford, UK; J.Silcock@bradford.ac.uk

Andrew Taylor, ISCOMAT Patient Led Steering Group, Manchester UK; ataylor102@outlook.com

Amanda J. Farrin Leeds Institute for Clinical Trials Research, Clinical Trials Research Unit, University of Leeds, Leeds, UK LS2 9JT; A.J.Farrin@leeds.ac.uk

Chris P. Gale Leeds Institute of Cardiovascular and Metabolic Medicine, University of Leeds, Leeds, UK; Leeds Institute for Data Analytics, University of Leeds, Leeds, UK; Department of Cardiology, Leeds Teaching Hospitals NHS Trust, Leeds, UK; c.p.gale@leeds.ac.uk


For peer review only - http://bmjopen.bmj.com/site/about/guidelines.xhtml

**Abstract**

**Introduction**

Heart failure affects 26 million people globally, approximately 900 thousand people in the UK, and is increasing in incidence. Appropriate management of medicines for heart failure at the time of hospital discharge reduces readmissions, improves quality of life and increases survival. The ISCOMAT trial tests the effectiveness of the Medicines at Transition Intervention (MaTI) which aims to enhance self-care and increase community pharmacy involvement in the medicines management of heart failure patients.

**Methods and analysis**

ISCOMAT is a parallel-group cluster randomised controlled trial, randomising 42 National Health Service trusts with cardiology wards in England on a 1:1 basis to implement the MaTI or treatment as usual. Around 2100 patients over the age of 18, admitted to hospital with heart failure with at least moderate left ventricular systolic dysfunction within the last 5 years, and planned discharge to the geographical area of the cluster will be recruited. The MaTI consists of training for staff, a toolkit for participants, transfer of discharge information to community pharmacies, and a medicines reconciliation/review. Treatment as usual is determined by local policy and practices. The primary outcome is a composite of all-cause mortality and heart failure-related hospitalisation at 12 months post-registration obtained from national electronic health records. The key secondary outcome is continued prescription of guideline-indicated therapies at 12-months measured via patient-reported data and Hospital Episode Statistics. The trial contains a parallel mixed-methods process evaluation and an embedded health economics study.

**Ethics and dissemination**

The study obtained approval from the Yorkshire and the Humber – Bradford Leeds Research Ethics Committee; REC reference 18/YH/0017. Findings will be disseminated via academic and policy conferences, peer-reviewed publications and social media. Amendments to the protocol are disseminated to all relevant parties as required.

**Registration**

ISRCTN66212970

**Key words**

Heart failure; cardiology; clinical trials; cluster trial; medicines

**Word count:** 3936


For peer review only - http://bmjopen.bmj.com/site/about/guidelines.xhtml
For peer review only

## Article summary

### Strengths and limitations of this study

- ISCOMAT is a trial which evaluates a process to optimise medicines use for patients discharged from hospital with heart failure to their usual place of residence
- The trial intervention was developed through experience-based co-design with patients and health care professionals
- The trial will use nationwide patient-level health records for the primary endpoint
- Due to intervention implementation requirements, patients were recruited from designated cardiology wards only
- Clinical events cannot be centrally adjudicated as they arise from electronic health records. As such this is a pragmatic trial with results generalizable to the 'real world' environment.


For peer review only - http://bmjopen.bmj.com/site/about/guidelines.xhtml

## Background

Heart failure affects 26 million people globally,[1] approximately 900 thousand people in the UK, and is increasing in incidence.[2] People with heart failure frequently use multiple healthcare services,[3] so the economic burden of heart failure is substantial and is further compounded by high rates of hospitalisation and subsequent readmission.[4]

Heart failure may be managed through a combination of pharmacological treatments at titrated doses including angiotensin-converting enzyme (ACE) inhibitors, beta-adrenoceptor antagonists (beta-blockers), mineralocorticoid/aldosterone receptor antagonists (MRA), angiotensin receptor neprilysin inhibitors, sodium-glucose transport protein 2 (SGLT2) inhibitors and diuretics as well as implanted devices.[5] Medicines optimisation reduces hospitalisation, improves quality of life and increases survival rates.[6] However, this requires the coordinated input of several healthcare professionals, following hospitalisation, which may be subject to variation.[7] Estimated readmissions rate within three months of discharge for patients with heart failure are as high as 50%[8], indicating a need to achieve improvements in the continuity of care at the time of transfer to the community.

The Medicines at Transitions Intervention (MaTI) was designed to improve the use of prescribed medicines when patients with heart failure are discharged from hospital. It was developed through qualitative research and co-design with patients and healthcare staff.[7 9] Intervention feasibility was tested in three healthcare areas with 31 participants in northern England in 2017.[10] Following the feasibility study, the intervention was refined before progressing to the main trial. [10] Following Medical Research Council guidance for the design and evaluation of complex interventions,[11] we report the protocol of the ISCOMAT trial, a cluster randomised controlled trial to evaluate the clinical and cost-effectiveness of the MaTI among heart failure patients.

## Methods and design

### Aim and Objectives

The overall aim of ISCOMAT (ISRCTN Registration 66212970) is to evaluate the effectiveness of the MaTI on the delivery of guideline recommended care and clinical outcomes for patients who are discharged from hospital with heart failure, when compared with treatment as usual.

The primary objective is to establish whether the MaTI reduces all-cause mortality and heart failure rehospitalisation measured over 12 months from registration.

The key secondary objective is to determine whether the intervention increases the proportion of participants being prescribed the composite of guideline-indicated therapies at 12 months post-registration (i.e. ACE inhibitor, angiotensin II receptor blocker (ARB) or salcubitril/valsartan, beta


For peer review only - http://bmjopen.bmj.com/site/about/guidelines.xhtml

blocker and/or ivabradine and MRA).[12]

Other secondary objectives, measured at 12-months post-registration unless stated otherwise, are to establish if the intervention:

- Increases time to all-cause mortality
- Increases time to heart failure-related rehospitalisation
- Increases time on guideline recommended (and indicated as above) cardiovascular medicines.
- Improves patient understanding of their medicines and satisfaction with medicines-related care at 2- and 6-week post-discharge and 12-months post-registration
- Increases number of days alive and out of hospital
- Reduces hospitalisations (all cause, cardiovascular related and heart failure)
- Reduces mortality due to specific causes.
- Is cost-effective

The internal pilot objective is to assess whether the trial recruitment meets the pre-defined progression criteria thresholds.

The objectives of the mixed-methods process evaluation are to determine intervention fidelity; the relationship between implementation/fidelity and outcomes; and the barriers or facilitators to the successful implementation and roll out of the intervention.[13]

**Trial Design**

This publication describes REC approved Study Protocol v4.0 and dated 08 August 2018.

ISCOMAT is a parallel-group cluster randomised controlled trial that aims to recruit 2100 participants hospitalised with heart failure from 42 National Health Service (NHS) trusts in England (approximately 50 participants per site) over a 12-month recruitment period. Clusters are defined as a distinct geographical area and consist of the NHS trust with a cardiology ward(s) and/or a Coronary Care Unit (CCU; defined as a ward with nursing staff with specific cardiac nursing expertise) together with associated community pharmacies within the Clinical Commissioning Group boundaries in that geographical area (Figure 1).

ISCOMAT was purposively designed to utilise the routine data sources available through providers such as NHS Digital, the Office for National Statistics and the National Heart Failure Audit. The use

For peer review only


For peer review only - http://bmjopen.bmj.com/site/about/guidelines.xhtml

of routine health records data has enabled the trial to reduce the burden of data collection on research teams and patients and ensure a high level of accuracy with minimal data loss for the primary endpoint. Furthermore, this design maximizing routine data sources helped to minimise the impact of COVID-19 on trial analysis plans.

For peer review only


For peer review only - http://bmjopen.bmj.com/site/about/guidelines.xhtml

[See separate TIFF File for Figure 1]
*Clinical Trials Research Unit (CTRU)
*Figure 1: Trial process diagram*

For peer review only


For peer review only - http://bmjopen.bmj.com/site/about/guidelines.xhtml

### Cluster eligibility

Acute NHS Trusts with accompanying cardiology wards that met all of the inclusion criteria were eligible to take part in the study as described in Table 1.

*Table 1: Site Eligibility Criteria*

| Eligibility Criteria | Details |
|---|---|
| Inclusion | Commitment to achieve the recruitment target |
| | Participation in the National Heart Failure Audit[1] |
| | Hospital Episode Statistics available via NHS Digital |
| | Deemed suitable to implement and deliver the intervention (as determined by the ISCOMAT team).[2] |
| Exclusion | Already providing medicines management deemed to be sufficiently similar to the MaTI |

[1] Participation in UK National Heart Failure Audit was required to facilitate acquiring the dataset from NICOR
[2] Sites were deemed suitable where there was at least one designated cardiology ward and/or CCU and had an existing mechanism to communicate with community pharmacies for all heart failure patients (or were willing to introduce one).

For peer review only

### Randomisation and blinding

NHS Trusts were required to obtain all necessary local management approvals prior to randomisation and this was used to evidence consent. Randomisation was undertaken independently by the Leeds Clinical Trials Research Unit (CTRU). Clusters were allocated on a 1:1 basis either to implement the MaTI or treatment as usual (TAU) using minimisation with a random element, minimised by:

- geographical region, as defined by National Institute for Health Research Clinical Research Networks;[14]
- type of hospital (university hospital or non-university hospital as defined by membership of the University Hospital Association)[15]; and
- method of clinical handover/information transfer to community pharmacy (electronic system, e.g. PharmOutcomes;[16] or manual system, e.g. standard email, fax, letter or phone).

### Participant eligibility and registration

Patient recruitment commenced approximately four weeks following cluster randomisation. Potential participants were screened following admission to hospital and recruited prior to discharge. Patient screening, consent, recruitment and baseline data collection was completed by research staff, who had no role in the delivery of care or treatment and, wherever possible, were


For peer review only - http://bmjopen.bmj.com/site/about/guidelines.xhtml

blinded to the cluster allocation to mitigate selection bias.[17] Site staff were asked to self-report instances of unblinding to CTRU. This was monitored at monthly Trial Management Group Meetings (TMG) and annual Trial Steering Committee (TSC) meetings. Consented participants meeting the eligibility criteria described in Table 2 Table 2 are included in the trial.

*Table 2: Participant Eligibility Criteria*

| **Eligibility Criteria** | **Details** |
|---|---|
| Inclusion | Admitted or transferred to a ward participating in the ISCOMAT trial |
| | Heart failure with evidence of at least moderate left ventricular systolic dysfunction* confirmed within the last 5 years. **Defined as: a left ventricular ejection fraction of less than or equal to 44%, quantified via echocardiogram; or the equivalent of this, if quantified by a different imaging modality.* |
| | Aged 18 years or over at hospital admission. |
| | Planned discharged from recruiting hospital to their home (defined by usual place of residence) or a care home. |
| | Planned discharge within geographical area of that cluster |
| | Capacity to provide informed consent |
| | Provide informed consent |
| Exclusion | Patients in a terminal phase of illness / end of life care pathway who were not expected to survive beyond 6 weeks post-discharge . |
| | Patients already participating in ISCOMAT (e.g., re-admissions) |

Documented reasons for ineligibility or declining participation were closely monitored by the trial team as part of a regular review of recruitment progress, to ensure generalisability of study results in accordance with CONSORT reporting guidelines, and to highlight issues in identifying or recruiting patients during the internal pilot.

Following confirmation of written informed consent and eligibility, participants were registered into the trial by an authorised member of staff at the trial research site, via a central automated 24-hour online registration system hosted at CTRU.


For peer review only - http://bmjopen.bmj.com/site/about/guidelines.xhtml

## Trial status

The first trial site was randomised on 16th April 2018, opened to recruitment on 1st June 2018 and the first participant was recruited on 8th June 2018. A total of 44 clusters were randomised, of which 43 opened to recruitment and registered at least one participant. Overall 1641 participants consented to take part. Recruitment was due to end on 31st December 2019 but was extended to 31st December 2020. Following discussion with the sponsor and in line with government guidance, recruitment at all sites paused on 18th March 2020 due to the COVID-19 pandemic and following consultation with the TSC/Programme Steering Committee (PSC)/funder the decision was taken to close ISCOMAT to recruitment on 28th July 2020.

## Governance

The trial is funded by a National Institute for Health Research (NIHR) Programme Grant for Applied Research (Reference: RP-PG-0514-20009. The views expressed in this paper are those of the authors and not necessarily those of the NIHR or the Department of Health and Social Care. The trial is sponsored by Bradford Teaching Hospitals NHS Foundation Trust.

## Intervention

The MaTI was initiated by the discharge nurse (or equivalent) and continued post-discharge through involvement of the community pharmacist. MaTI comprises of:

1) Patient-held personalisable medicines toolkit containing: information about heart failure, medicines and their effects; details of the patients' healthcare team; a checklist of medicines-related activity that should occur in hospital before discharge, at discharge and once the patient has returned home; a traffic-light guide to symptoms plus actions to take if symptoms worsen. This will be delivered to patients face-to-face by a nurse of pharmacist from the cardiology ward as part of routine care at intervention sites.
2) Enhanced communication between hospitals and the patients' community pharmacists through transfer of the patient's discharge letter and medicines list to community pharmacy, with a request for the community pharmacist to reconcile the patient's medicines and invite the patient for a medicines use review (MUR) or a discussion about their medicines.
3) Increased engagement of community pharmacists with patient care after discharge comprising medicines reconciliation and invitation to the patient to attend the pharmacy for a MUR or a medicines discussion with the community pharmacist.

The intervention was delivered to all eligible patients as part of routine care. Clinical staff were not aware which patients have consented to data collection and evaluation.


For peer review only - http://bmjopen.bmj.com/site/about/guidelines.xhtml

### Intervention Training

Clusters randomised to the intervention were asked to identify key members of the clinical team involved in medicines management (e.g. ward nurses, hospital pharmacists, primary care heart failure nurses) to complete intervention training online and in-person. The intervention team aimed to train around 5-7 staff members in each hospital site to enable the site coordinator and nominated staff to brief others about the MaTI. The online training was developed with the Centre for Pharmacy Postgraduate Education (www.cppe.ac.uk) to enable staff to gain an understanding of the MaTI. Face-to-face training explained the background to MaTI (importance, purpose, design, outcomes) and went through the supporting materials/ guides designed to aid facilitation of delivery. Approximately one month was allowed in each cluster for training and embedding the MaTI into routine practice prior to study recruitment commencing. Each cluster identified one member of staff to adopt the 'site coordinator' role, responsible for ensuring the intervention was implemented according to the guidance and training provided. The number and role of hospital staff trained in each cluster was dependent on the structure of cardiology/pharmacy services and available staff. After 3-4 weeks, further follow up visits were arranged to ensure adherence to the protocol.

Staff delivery of the intervention on an individual level was communicated to intervention team regularly to support ongoing implementation of the intervention. Additional support and training were available when required.

### Measuring adherence to the intervention

The TMG monitored adherence to intervention protocols every month by reviewing delivery of key intervention components across all intervention sites. Performance feedback was provided to site teams via monthly emails. Data on adherence was also reviewed at annual TSC meetings.

### Treatment as usual

Treatment as usual was determined by local Trust policies and practices. Variations in treatment as usual were expected across NHS cardiology services in England and documented in site feasibility assessments, during recruitment, and at the end of follow-up.

### Process Evaluation

A parallel embedded process evaluation aims to inform the trial findings and aid potential future wider scale implementation. The process evaluation involved six purposively selected intervention sites using a mixed-methods design. Fidelity and barriers/enablers of implementation of MaTI were explored using observation, interviews of up to 20 patients and 40 healthcare professionals, staff

For peer review only


For peer review only - http://bmjopen.bmj.com/site/about/guidelines.xhtml

surveys and trial-specific data collection on MaTI adherence.[13] A parallel mixed analysis is planned. Qualitative data is thematically analysed using Framework analysis and survey data is analysed using descriptive statistics. Data is synthesised, triangulated and mapped to the Consolidated Framework for Implementation Research (CFIR)[18 19] where appropriate. A full description of methods are published in Powell et al.[13]

**Outcome measures**

The primary outcome is the binary composite endpoint of all-cause mortality and heart failure-related hospitalisation within 12 months post-registration. Mortality data is obtained from the Civil Registration Death data from the Office for National Statistics, and hospitalisation data from Hospital Episode Statistics (HES)[20] from NHS Digital. Routine data for the analysis of primary and secondary outcomes is supported by site reported and patient reported data where appropriate.

Box 1: Definition of hospitalisation

| |
|---|
| **Definition of hospitalisation:** Hospitalisation is defined as an acute inpatient admission (regardless of length of stay). A hospitalisation will be considered heart-failure related if the primary reason / diagnosis for admission included ICD-10 codes or procedural classifications related to heart failure |

The key secondary outcome is participants still being prescribed at least one of the guideline recommended therapies at 12 months in each of the following three groups:

- ACE inhibitor (ACEI); angiotensin II receptor blocker (ARB); salcubitril/valsartan
- Beta blocker; ivabradine
- Mineralocorticoid receptor antagonist (MRA)

For patients with contraindications to any of the three groups, the endpoint will be derived with respect to groups that are indicated. Data is collected via patient-reported questionnaires collected at baseline, 3-month and 6-month post-registration. We also plan to obtain data from the NHS Business Service Authority on medicines dispensed in primary care, via NHS Digital.

The following secondary outcomes are collected over the 12-month post-registration via routine data sources:

- Individual components of the primary endpoint regarded as time to event endpoints. Time will be measured from registration to the first day of heart failure rehospitalisation or death
- Length of time on guideline recommended cardiovascular medications
- Days alive and out of hospital: defined as the number of days in the year beginning the day after registration that the patient spends alive and not in hospital
- Time to cardiovascular-related hospitalisation and all cause hospitalisation


For peer review only - http://bmjopen.bmj.com/site/about/guidelines.xhtml

- Mortality due to specific causes
- Healthcare resource use

The following secondary outcomes are measured at baseline, and 3-month and 12-month post-registration unless stated otherwise:

- Patient satisfaction with their medicines care and patient understanding of their medicines; measured via a patient experience survey at 2 and 6-weeks post-discharge, and 12-months post-registration with a 10-point Likert scale. For intervention participants data on the use of the MaTI over a 12-month post-registration period is also captured.
- EuroQol5-Dimension Health Questionnaire (3 levels) (EQ-5D-3L)[21]; measures health utility (quality of life) comprising five dimensions: mobility, self-care, usual activities, pain/discomfort and anxiety/depression. Dimension responses are combined and converted into a summary index (0 for dead, 1 for perfect health and negative values for states worse than death).
- Healthcare resource use: additional to routine datasets, we capture patient reported healthcare resource use using standard self-reported resource use questionnaires, to include hospital attendances, GP, nurse, specialist heart failure nurse and pharmacy contact.

**Data collection**

The outcome assessment schedule is outlined in Table 3**Error! Reference source not found.**. All personal information is processed in accordance with the Data Protection Act 2018[22] (and any successor legislation). Appropriate storage, restricted access and disposal arrangements for personal and clinical details are in place. Archiving is for a minimum of 10 years.

*Routine Data*

ISCOMAT will obtain data from routine providers NHS Digital (including HES and ONS data) and NICOR and the UK National Heart Failure Audit for the primary and secondary outcomes as outlined above. The trial was designed to use data from national routine sources to maximise efficiency in answering the research questions, ensure consistency across multiple sites, and minimise burden on patients and research staff at hospitals.

*Clinical Data*

Recruiting teams collected information on study eligibility and details of the participants' admission and discharge, including heart failure history, clinical details, primary aetiology for heart failure, detailed medicines information, and related unexpected serious adverse events (RUSAEs). All-


For peer review only - http://bmjopen.bmj.com/site/about/guidelines.xhtml

cause mortality and re-admission data were collected throughout the study up until 12 months post registration from hospital sites and electronic health records (NHS Digital). Consent was sought to obtain data from electronic health records – including secondary care records and NHS Digital.

*Participant reported data*

Patient self-reported baseline measures were collected in hospital inpatient setting. Patient reported outcomes at 2- and 6-week post discharge, and 3- and 12-months post registration were collected via post. When questionnaires were not returned, the CTRU sent postal reminders.

*Intervention fidelity data and quantitative and qualitative data from observations, participant and staff interviews and surveys*

Clinical teams, randomised to the intervention, returned a checklist of seven steps for each trial participant, confirming completion of intervention activity and local pharmacy information. The CTRU sought data from the participants' community pharmacy following discharge. Details about the heart failure service models and the clinical role of those staff responsible for the delivery of the MaTI were documented.

*Table 3: Schedule of enrolment, interventions and assessments for participants*

| Assessment | Type | Method of Completion | Timepoint | | | | | |
|---|---|---|---|---|---|---|---|---|
| | | | Screening | Baseline | 2 weeks post-discharge | 6 weeks post-discharge | 3 months post-registration | 12 months post-registration |
| PARTICIPANT DATA | | | | | | | | |
| Screening (demographics / assessment of eligibility) | CRF[1] | CRN[2] / local research staff | X | | | | | |
| Consent | Consent Form | Self-completion | | X | | | | |
| Eligibility | CRF | CRN / local research staff | | X | | | | |
| Demographics | CRF | CRN / local research staff | | X | | | | |
| Contact Details | CRF | CRN / local research staff | | X | | | | |
| Admission/discharge details | CRF | CRN / local research staff | | X | | | | |
| Health Related Quality of Life (EuroQol EQ-5D-3L) | Questionnaire booklet (post) | Self-completion | | X | | | X | X |


For peer review only - http://bmjopen.bmj.com/site/about/guidelines.xhtml
For peer review only

| | | | | | | | | |
|---|---|---|---|---|---|---|---|---|
| Healthcare Resource Use (in last 3 months) | Questionnaire booklet (post) | Self-completion | | X | | | X | X |
| Current heart failure medications | Questionnaire booklet (post) | Self-completion | | | | | X | X |
| Patient Experience Survey | Questionnaire booklet (post) | Self-completion | | | X | X | | X |
| Primary outcome (all-cause mortality and HF rehospitalisation) | Routine data / CRF | Data Access Request: Routine data providers > CTRU[3] | | DATA EXTRACTS TO BE AGREED WITH DATA PROVIDERS TO ALLOW 12 MONTH DATA COLLECTION | | | | |
| Secondary outcomes | Routine data / CRF | Data Access Request: Routine data providers > CTRU | | DATA EXTRACTS TO BE AGREED WITH DATA PROVIDERS TO ALLOW 12 MONTH DATA COLLECTION | | | | |
| Patient status | CRF | CTRU | | ONGOING | | | | |

| SITE DATA | | | | | |
|---|---|---|---|---|---|
| Assessment | Type | Method of Completion | Baseline | 6 months post-randomisation | 12 months post-implementation of intervention |
| Site questionnaire | Questionnaire Booklet | CTRU | X | X | X |

| INTERVENTION DATA | | | | |
|---|---|---|---|---|
| Assessment | Type | Method of Completion | 3 months post-registration | 6 months post-implementation of intervention |
| Adherence / fidelity data (collection of patient checklists to confirm what happened at discharge, MUR review confirmation from the pharmacy) | CRF | CTRU | ONGOING | |
| Interviews - Patients | Process Evaluation | Research Fellow | X | |
| Interviews - Staff | Process Evaluation | Research Fellow | | x |
| Survey - Staff | Process Evaluation | Research Fellow | | x |
| Observations - ward | Process Evaluation | Research Fellow | | x |

[1]Case report form (CRF)
[2]Clinical Research Network (CRN)
[3]Clinical Trials Research Unit (CTRU)

## Statistical methods

## Sample size


For peer review only - http://bmjopen.bmj.com/site/about/guidelines.xhtml

Sample size calculations were based on previous research[23 24] indicating that the combined primary event rate is likely to be at least 20% in control sites. With 42 clusters, an estimated intracluster correlation coefficient (ICC) of 0.01, a coefficient of variation in cluster size of 0.23, and 15% loss to follow-up (based on studies in this patient group),[25] 50 patients with heart failure in each cluster (2100 in total) are required to provide at least 80% power at 5% significance level to detect a minimal clinically important difference (MCID) of 6%.[25] The MaTI is delivered to each heart failure participant by dispersed teams across the care transition (secondary-care, primary-care, community pharmacy), therefore, the anticipated ICC is likely to be relatively low.

This sample size also provides power in excess of 90% to detect a MCID of 12% in the key secondary outcome even with the most conservative assumption for the control rate.

**Internal pilot**

Descriptive statistics were used to evaluate progression criteria assessing site-level recruitment rates. This analysis informed study continuation beyond the internal pilot phase. The progression criterion was assessed at 6-months after recruitment commenced, based on the following traffic light system of green (go), amber (review) and red (stop):

Green: ≥4 patients/month/NHS trust;

Amber: ≥2 but <4 patients/month/NHS trust;

Red: <2 patients/month/NHS trust.

**Data analysis**

A full statistical analysis plan (SAP) will be finalised prior to any analyses. The CTRU will conduct the statistical analysis and data summaries on an intention-to-treat (ITT) population, which is defined as all clusters randomised and all participants registered regardless of non-compliance with the protocol or withdrawal from the study. The study is conducted and will be reported according to the CONSORT extensions for cluster trials[26] and CONSORT-ROUTINE[27] for trials conducted using cohorts and routinely collected data. Baseline data will be summarised by treatment group and at the participant level and/or the cluster level using descriptive statistics as appropriate.

Additionally, the SAP will describe the derivation of outcomes and detail the analyses for each outcome measure. The analyses models will reflect clustering and include fixed effects for the minimisation factors with clusters as random effects, see Table 4.


For peer review only - http://bmjopen.bmj.com/site/about/guidelines.xhtml

A complier average causal effect (CACE) analysis approach to estimate the treatment effect for the primary outcome amongst compliant clusters will be considered depending on levels of adherence to the intervention as intended. Definitions of adherence and pre-defined thresholds will be outlined in the SAP and made without reference to the effectiveness data.

No formal interim analyses were planned. Blinded interim reports were presented to the TSC containing descriptive information. This included data on recruitment, follow-up, adherence, safety and data quality. These were presented by randomised arm if specifically requested as such, with treatments masked. As participants were recruited following cluster randomisation, monitoring of baseline characteristics by arm are presented to assess post-randomisation selection bias. A single final analysis is planned when all follow-up data has been entered onto trial database, and the database is cleaned and locked.

In the cost-effectiveness analysis, the primary outcome is Quality Adjusted Life Years (QALYs) compared to costs from an NHS and PSS perspective. A secondary cost effectiveness analysis will look at the incremental cost per all-cause deaths prevented and re-hospitalisation prevented. Resource use associated with 'treatment as usual' and the intervention will be collected through the health resource use questionnaire administered to patients. Prescription and hospitalisation data will be collected from the data linkage outputs and supported where necessary by the patient health resource use questionnaire at 3 & 12 months. Unit costs for health service resources will be obtained from national sources (e.g. Personal Social Services Research Unit [PSSRU] British National Formulary for medicines). The intervention cost will include any enhancement of existing medicines management components and any 'new' components of the MaTI (e. g. the staff training module or necessary enhancements to existing community pharmacy medicines services). We will assess uncertainty using a within trial probabilistic sensitivity analysis undertaken using Monte Carlo simulation with results presented as Incremental Cost-Effectiveness rations (ICERs) and cost effectiveness acceptability curves.

For further detail on outcome measures, see Table 4.

*Table 4: Data analysis plan for primary and secondary outcomes and health economic analysis*

| Analysis activities | Assessed At | Statistical methods |
|---|---|---|
| **Primary Endpoint – all-cause mortality and heart failure-related rehospitalisation within** | 12 months post registration | The primary endpoint will be analysed using a two-level logistic regression model with participants nested within clusters, with clusters treated as random effects. The model will be adjusted for the following fixed effects: the cluster level stratification variables |


For peer review only - http://bmjopen.bmj.com/site/about/guidelines.xhtml

| | | |
|---|---|---|
| **12 months post-registration** | | (level 2): geographical region, type of hospital and method of clinical handover/information transfer to community pharmacy, and patient level covariates (level 1) and other relevant known predictors of outcome. Results will be expressed as point estimates, with corresponding 95% CIs, p-values. An estimate of the ICC and corresponding CI will also be presented. |
| **Key secondary endpoint** | 12 months post-registration | This endpoint will be analysed using a similar modelling strategy as the primary endpoint. |
| **Other secondary endpoints** | 12 months post-registration | Time-to-event analyses will be by multi-level shared frailty models with the event right-censored at either 12 months follow-up, date of death or date of withdrawal, whichever is earliest.<br><br>Days alive and out of hospital will be analysed using mixed effects Poisson regression model.[28] with data censored at either 12-months post-registration, death or withdrawal, whichever is earliest.<br><br>Length of time on guideline recommended cardiovascular medications and cause specific death endpoints will be summarized descriptively. |
| | 2 & 6 weeks post-discharge; 12 months post-registration | Patient understanding of their medicines and satisfaction with medicines-related care will be analysed using Ordinal logistic regression with clustering model to compare participant responses between treatment arms. |
| **Cost-effectiveness analysis** | 12 months post-registration | The primary outcome will be Quality Adjusted Life Years (QALYs). A secondary cost effectiveness analysis will look at the incremental cost per all-cause deaths prevented and re-hospitalisation prevented.<br><br>In order to assess the long-term cost-effectiveness of the intervention, compared to treatment as usual, a decision analytic cost-effectiveness model will be used to estimate the expected incremental cost per QALY. The model will include a Value of Information Analysis (VOIA) to assess the value of undertaking further research to reduce decision uncertainty in the model. The perspective will be the same as the within-trial analysis but the time horizon will be the lifetime of the individual to capture the full impact of any mortality differences on the long-term cost-effectiveness. Estimation of health-related quality of life will use QALYs and discounting will be at the same rate as for the within trial analysis. Parameter uncertainty will be addressed using probabilistic sensitivity analysis and running Monte Carlo simulations. Results will meet |


For peer review only - http://bmjopen.bmj.com/site/about/guidelines.xhtml
For peer review only

| | | |
|---|---|---|
| | | international CHEERS standards of reporting and results will be presented as Incremental Cost-Effectiveness rations (ICERs), Incremental Net Health and Monetary Benefits and Cost Effectiveness Acceptability Curves (CEACs). |

### Data monitoring

Data is monitored for quality and completeness by the Clinical Trials Research Unit (CTRU) using established verification, validation and checking processes. The Trial Management Group (TMG) comprising the chief investigator (CI), CTRU team, other co-applicants and PPI representative will be responsible for the on-going management of the trial. The TMG is responsible for any audits for trial conduct required. The TMG reports to the Trial Steering Committee (TSC) annually. The TSC is formed with an independent chair, health economist and statisticians.

### Methods to handle missing data

Missing data are expected and the proportion of missing data will be compared between intervention and control groups. Sensitivity analyses of the primary endpoint will be conducted to assess the impact of missing data, the choice of imputation model and assumptions around the missingness mechanism, as appropriate.

### Ethics and dissemination

The trial received favourable REC opinion from Yorkshire and the Humber – Bradford Leeds Health Research Ethics Committee on 30/01/2018 with reference number 18/YH/0017
The trial was registered (ISRCTN66212970).

The trial is sponsored by Bradford Teaching Hospitals NHS Foundation Trust, coordinated by the CTRU at the University of Leeds and supported by a TMG and Programme Management Group (PMG) TSC. The TSC includes independent external experts who provide overall supervision for a trial on behalf of the Trial Sponsor and Trial Funder.

This longstanding and ongoing engagement with stakeholders provides a direct pathway to impact for the outputs of this research. Our Patient-Led Steering Group (PLSG) will inform our dissemination strategy and its members will play an active role in the format and content of academic papers (specifically patient implications) and will present at local, regional and national conferences and meetings.

### Patient and public involvement

ISCOMAT has a Patient-Led Steering Group that has contributed throughout the programme.


For peer review only - http://bmjopen.bmj.com/site/about/guidelines.xhtml

Patient representatives have membership of the TMG, Programme Management Group and TSC. The PLSG has played an integral role in several aspects of the study, including intervention development, the development of data collection tools and the analysis of qualitative interview data. Members of the group have also served as co-authors in programme and trial publications.

**COVID-19 pandemic**

The COVID-19 adversely affected routine NHS services, and there was a decline in admissions with cardiovascular disease.[29 30] Following national NIHR guidance, sponsor decision and TSC review, the ISCOMAT trial was paused in March 2020 and formally closed to recruitment in July 2020. Uncertainty about the post-COVID landscape raised concerns for intervention relevance and deliverability, future research capacity and participant willingness to be involved in research, number of admissions and organisation of heart failure services in sites, plus external policy changes, such as medicines use reviews (MURs). Additional concerns included the effect of site withdrawals following a restart on the trial design and increased post-COVID death rates on our findings. The decision to close the trial was supported by statistical review that concluded that closing recruitment would not have a significant impact on the statistical power or analysis of the primary and key secondary outcomes, given on the proportion of the target sample size recruited to date.

**Conclusion**

Heart failure outcomes, such as mortality and hospital readmission can be improved though the optimal management of medicines. Optimising treatment after transitions of care requires the coordinated input of different healthcare organisations and professions, and patients.

ISCOMAT is a pragmatic multi-centre cluster RCT that aims to recruit 2100 participants hospitalised with heart failure from across 42 hospitals, approximately 50 participants per site, over a 12-month recruitment period. The ISCOMAT trial will establish if a complex intervention comprising enhanced and personalised communication for patients about their medicines and the transfer of discharge information to the patients' community pharmacy will reduce hospital admissions and mortality over a 12-month period. As such, ISOMAT has the potential to improve the safety and continuity of medicines at care transitions for hospitalised patients with heart failure, and reduce mortality and subsequent hospitalisations.

For peer review only


For peer review only - http://bmjopen.bmj.com/site/about/guidelines.xhtml

**Acknowledgements**

We would like to thank Alison Blenkinsopp, Gerry Armitage, Theo Rayner, Ian Kellar, Jan Speechley, the ISCOMAT Patient-led Steering Group, the ISCOMAT Trial Management Group, Trial Steering Committee and Programme Steering Committee.

**Author contributions**

IH, BF, SH, BC, AF, SA, DA, CB, LB, HI, EM, PG, CP, JS, AT, AJF, CPG developed the methodology and detail of the ISCOMAT trial protocol. LM drafted the manuscript, with all authors reviewing the draft critically for intellectual content. All authors approved the final version submitted for publication.

**Funding Statement**

The study was funded as part of a National Institute for Health Research Programme Grant for Applied Research (RP-PG-0514-20009). The views expressed in this paper are those of the authors and not necessarily those of the NHS, the NIHR or the Department of Health.

**Trial Sponsor**

The trial is sponsored by Bradford Teaching Hospitals NHS Foundation Trust.

**Data statement**

All data requests should be submitted to the corresponding author for consideration. Access to anonymised data may be granted following review.

**Disclaimer**

The views expressed are those of the authors and not necessarily those of the NIHR.

**Competing interests Statement.**

None declared.

For peer review only


For peer review only - http://bmjopen.bmj.com/site/about/guidelines.xhtml

## References


1. Ambrosy AP, Fonarow GC, Butler J, et al. The Global Health and Economic Burden of Hospitalizations for Heart Failure: Lessons Learned From Hospitalized Heart Failure Registries. *Journal of the American College of Cardiology* 2014;63(12):1123-33. doi: https://doi.org/10.1016/j.jacc.2013.11.053
2. Conrad N, Judge A, Tran J, et al. Temporal trends and patterns in heart failure incidence: a population-based study of 4 million individuals. *The Lancet* 2018;391(10120):572-80. doi: 10.1016/S0140-6736(17)32520-5
3. Kim D, Hayhoe B, Aylin P, et al. Health service use by patients with heart failure living in a community setting: a cross-sectional analysis in North West London. *British Journal of General Practice* 2020;70(697):e563. doi: 10.3399/bjgp20X711749
4. Shafie AA, Tan YP, Ng CH. Systematic review of economic burden of heart failure. *Heart Failure Reviews* 2018;23(1):131-45. doi: 10.1007/s10741-017-9661-0
5. Ponikowski P, Voors AA, Anker SD, et al. 2016 ESC Guidelines for the diagnosis and treatment of acute and chronic heart failure: The Task Force for the diagnosis and treatment of acute and chronic heart failure of the European Society of Cardiology (ESC)Developed with the special contribution of the Heart Failure Association (HFA) of the ESC. *European Heart Journal* 2016;37(27):2129-200. doi: 10.1093/eurheartj/ehw128
6. McMurray JJV, Adamopoulos S, Anker SD, et al. ESC Guidelines for the diagnosis and treatment of acute and chronic heart failure 2012 The Task Force for the Diagnosis and Treatment of Acute and Chronic Heart Failure 2012 of the European Society of Cardiology. Developed in collaboration with the Heart Failure Association (HFA) of the ESC. *Eur J Heart Fail* 2012;14(8):803-69. doi: 10.1093/eurjhf/hfs105
7. Fylan B, Marques I, Ismail H, et al. Gaps, traps, bridges and props: a mixed-methods study of resilience in the medicines management system for patients with heart failure at hospital discharge. *Bmj Open* 2019;9(2) doi: ARTN e023440
10.1136/bmjopen-2018-023440
8. Simms AD, Baxter PD, Cattle BA, et al. An assessment of composite measures of hospital performance and associated mortality for patients with acute myocardial infarction. Analysis of individual hospital performance and outcome for the National Institute for Cardiovascular Outcomes Research (NICOR). *European Heart Journal: Acute Cardiovascular Care* 2013;2(1):9-18.
9. Raynor DK, Ismail H, Blenkinsopp A, et al. Experience-based co-design-Adapting the method for a researcher-initiated study in a multi-site setting. *Health Expect* 2020 doi: 10.1111/hex.13028 [published Online First: 2020/02/12]
10. Fylan B, Ismail H, Hartley S, et al. A non-randomised feasibility study of an intervention to optimise medicines at transitions of care for patients with heart failure. *Pilot and Feasibility Studies* 2021;7(1):85. doi: 10.1186/s40814-021-00819-x
11. Council MR. Developing and evaluating complex interventions [Available from: https://mrc.ukri.org/documents/pdf/complex-interventions-guidance/.
12. National Institute for Health & Care Excellence (NICE). Nice guideline [NG106}. Chronic heart failure in adults: diagnosis and management 2018 [Available from: https://www.nice.org.uk/guidance/ng106.
13. Powell C, Breen L, Fylan B, et al. Improving the Safety and Continuity Of Medicines management at Transitions of care (ISCOMAT): protocol for a process evaluation of a



For peer review only - http://bmjopen.bmj.com/site/about/guidelines.xhtml

cluster randomised control trial. 2020;10(11):e040493. doi: 10.1136/bmjopen-2020-040493 %J BMJ Open
14. National Institute for Health Research. Clinical Research Network [Available from: https://www.nihr.ac.uk/explore-nihr/support/clinical-research-network.htm accessed 18/01/2021 2021.
15. AUKUH Member Organisations. https://www.universityhospitals.org.uk/ [accessed 06/06/2021]
16. Pharmoutcomes [program].
17. Puffer S, Torgerson D, Watson J. Evidence for risk of bias in cluster randomised trials: review of recent trials published in three general medical journals. *BMJ* 2003;327(7418):785-9. doi: 10.1136/bmj.327.7418.785 [published Online First: 2003/10/04]
18. Gale NK, Heath G, Cameron E, et al. Using the framework method for the analysis of qualitative data in multi-disciplinary health research. *BMC medical research methodology* 2013;13(1):117.
19. Damschroder LJ, Aron DC, Keith RE, et al. Fostering implementation of health services research findings into practice: a consolidated framework for advancing implementation science. *Implementation science* 2009;4(1):50.
20. NHS Digital. Hospital Episode Statistics (HES) Data [Available from: https://digital.nhs.uk/data-and-information/data-tools-and-services/data-services/hospital-episode-statistics accessed 18/01/2021 2021.
21. Williams A. Euroqol - a New Facility for the Measurement of Health-Related Quality-of-Life. *Health Policy* 1990;16(3):199-208.
22. Data Protection Act 2018, 2018.
23. Shah RV, McNulty S, O'connor CM, et al. Effect of admission oral diuretic dose on response to continuous versus bolus intravenous diuretics in acute heart failure: an analysis from diuretic optimization strategies in acute heart failure. *American heart journal* 2012;164(6):862-68.
24. Palazzuoli A, Pellegrini M, Ruocco G, et al. Continuous versus bolus intermittent loop diuretic infusion in acutely decompensated heart failure: a prospective randomized trial. *Critical care* 2014;18(3):R134.
25. McMurray J, Packer M, Desai A, et al. PARADIGM-HF Investigators and Committees. *Angiotensin-neprilysin inhibition versus enalapril in heart failure N Engl J Med* 2014;371(11):993-1004.
26. Campbell MK, Piaggio G, Elbourne DR, et al. Consort 2010 statement: extension to cluster randomised trials. *Bmj* 2012;345:e5661. doi: 10.1136/bmj.e5661 [published Online First: 2012/09/07]
27. Kwakkenbos L, Imran M, McCall SJ, et al. CONSORT extension for the reporting of randomised controlled trials conducted using cohorts and routinely collected data (CONSORT-ROUTINE): checklist with explanation and elaboration. 2021;373:n857. doi: 10.1136/bmj.n857 %J BMJ
28. Fanaroff AC, Cyr D, Neely ML, et al. Days Alive and Out of Hospital: Exploring a Patient-Centered, Pragmatic Outcome in a Clinical Trial of Patients With Acute Coronary Syndromes. *Circ Cardiovasc Qual Outcomes* 2018;11(12):e004755. doi: 10.1161/CIRCOUTCOMES.118.004755 [published Online First: 2018/12/19]
29. Mafham MM, Spata E, Goldacre R, et al. COVID-19 pandemic and admission rates for and management of acute coronary syndromes in England. *Lancet (London, England)* 2020;396(10248):381-89. doi: 10.1016/s0140-6736(20)31356-8 [published Online First: 2020/07/18]
30. Shoaib A, Van Spall HGC, Wu J, et al. Substantial decline in hospital admissions for heart failure accompanied by increased community mortality during COVID-19 pandemic.


For peer review only - http://bmjopen.bmj.com/site/about/guidelines.xhtml

*European Heart Journal - Quality of Care and Clinical Outcomes* 2021 doi: 10.1093/ehjqcco/qcab040

For peer review only


For peer review only - http://bmjopen.bmj.com/site/about/guidelines.xhtml

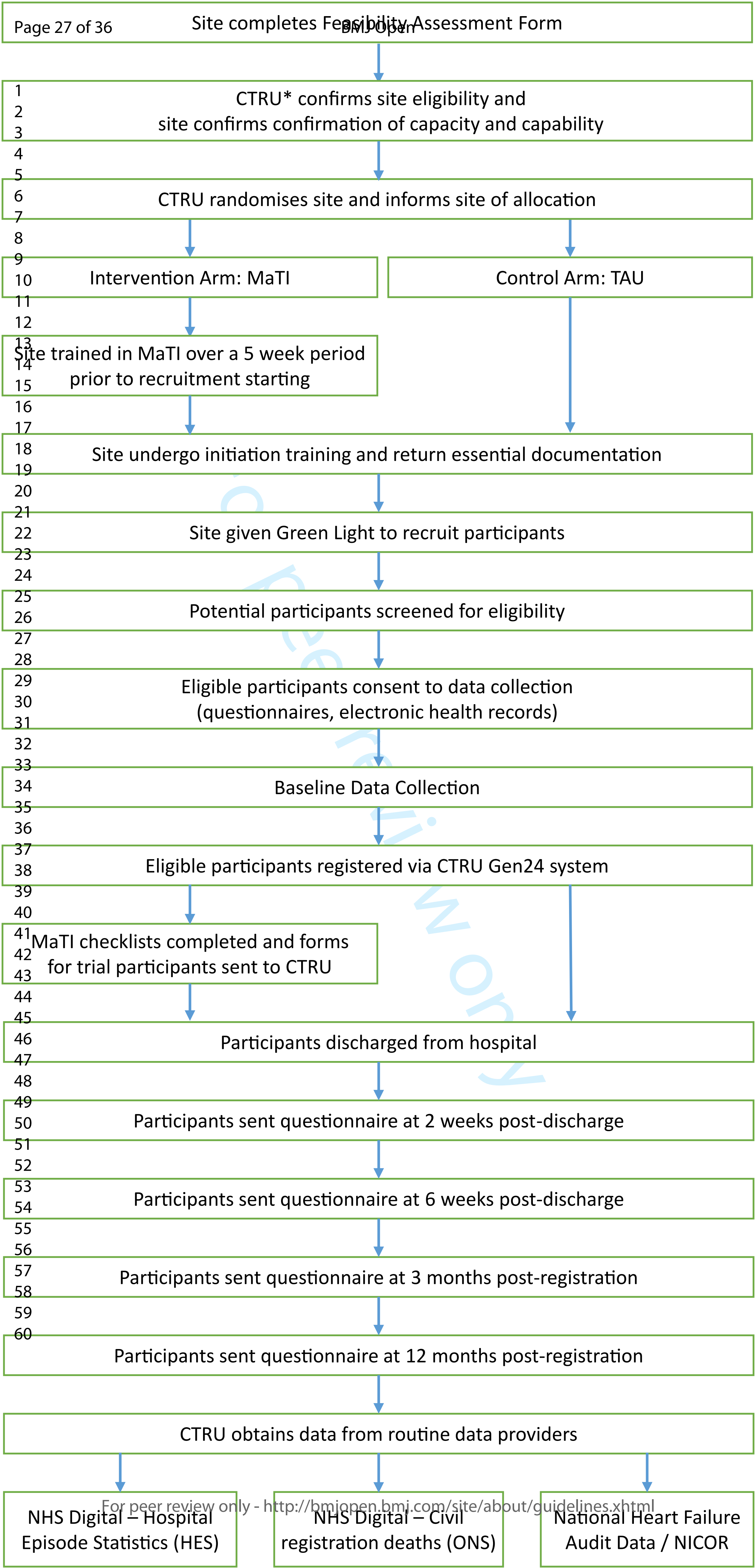
Site completes Feasibility Assessment Form
CTRU* confirms site eligibility and
site confirms confirmation of capacity and capability
CTRU randomises site and informs site of allocation
Intervention Arm: MaTI
Control Arm: TAU
Site trained in MaTI over a 5 week period
prior to recruitment starting
Site undergo initiation training and return essential documentation
Site given Green Light to recruit participants
Potential participants screened for eligibility
Eligible participants consent to data collection
(questionnaires, electronic health records)
Baseline Data Collection
Eligible participants registered via CTRU Gen24 system
MaTI checklists completed and forms
for trial participants sent to CTRU
Participants discharged from hospital
Participants sent questionnaire at 2 weeks post-discharge
Participants sent questionnaire at 6 weeks post-discharge
Participants sent questionnaire at 3 months post-registration
Participants sent questionnaire at 12 months post-registration
CTRU obtains data from routine data providers
NHS Digital – Hospital
Episode Statistics (HES)
NHS Digital – Civil
registration deaths (ONS)
National Heart Failure
Audit Data / NICOR

# Reporting checklist for protocol of a clinical trial.

Based on the SPIRIT guidelines.

## Instructions to authors

Complete this checklist by entering the page numbers from your manuscript where readers will find each of the items listed below.

Your article may not currently address all the items on the checklist. Please modify your text to include the missing information. If you are certain that an item does not apply, please write "n/a" and provide a short explanation.

Upload your completed checklist as an extra file when you submit to a journal.

In your methods section, say that you used the SPIRITreporting guidelines, and cite them as:

Chan A-W, Tetzlaff JM, Gøtzsche PC, Altman DG, Mann H, Berlin J, Dickersin K, Hróbjartsson A, Schulz KF, Parulekar WR, Krleža-Jerić K, Laupacis A, Moher D. SPIRIT 2013 Explanation and Elaboration: Guidance for protocols of clinical trials. BMJ. 2013;346:e7586

| | | Reporting Item | Page Number |
|---|---|---|---|
| **Administrative information** | | | |
| Title | #1 | Descriptive title identifying the study design, population, interventions, and, if applicable, trial acronym | 1 |
| Trial registration | #2a | Trial identifier and registry name. If not yet registered, | 3 |

For peer review only

| | | | |
|---|---|---|---|
| | | name of intended registry | |
| Trial registration: data set | #2b | All items from the World Health Organization Trial Registration Data Set | N/A |
| Protocol version | #3 | Date and version identifier | 6 |
| Funding | #4 | Sources and types of financial, material, and other support | 22 |
| Roles and responsibilities: contributorship | #5a | Names, affiliations, and roles of protocol contributors | 1-2; 21-2 |
| Roles and responsibilities: sponsor contact information | #5b | Name and contact information for the trial sponsor | 22 |
| Roles and responsibilities: sponsor and funder | #5c | Role of study sponsor and funders, if any, in study design; collection, management, analysis, and interpretation of data; writing of the report; and the decision to submit the report for publication, including whether they will have ultimate authority over any of these activities | 22 |
| Roles and responsibilities: committees | #5d | Composition, roles, and responsibilities of the coordinating centre, steering committee, endpoint adjudication committee, data management team, and other individuals or groups overseeing the trial, if applicable (see Item 21a for data monitoring committee) | 20 |

**Introduction**

For peer review only

| | | | |
|---|---|---|---|
| Background and rationale | #6a | Description of research question and justification for undertaking the trial, including summary of relevant studies (published and unpublished) examining benefits and harms for each intervention | 5 |
| Background and rationale: choice of comparators | #6b | Explanation for choice of comparators | 11 |
| Objectives | #7 | Specific objectives or hypotheses | 5 |
| Trial design | #8 | Description of trial design including type of trial (eg, parallel group, crossover, factorial, single group), allocation ratio, and framework (eg, superiority, equivalence, non-inferiority, exploratory) | |
| **Methods: Participants, interventions, and outcomes** | | | |
| Study setting | #9 | Description of study settings (eg, community clinic, academic hospital) and list of countries where data will be collected. Reference to where list of study sites can be obtained | 9 |
| Eligibility criteria | #10 | Inclusion and exclusion criteria for participants. If applicable, eligibility criteria for study centres and individuals who will perform the interventions (eg, | 10 |

For peer review only

| | | | |
|---|---|---|---|
| | | surgeons, psychotherapists) | |
| Interventions: description | #11a | Interventions for each group with sufficient detail to allow replication, including how and when they will be administered | 11 |
| Interventions: modifications | #11b | Criteria for discontinuing or modifying allocated interventions for a given trial participant (eg, drug dose change in response to harms, participant request, or improving / worsening disease) | N/A |
| Interventions: adherance | #11c | Strategies to improve adherence to intervention protocols, and any procedures for monitoring adherence (eg, drug tablet return; laboratory tests) | 12 |
| Interventions: concomitant care | #11d | Relevant concomitant care and interventions that are permitted or prohibited during the trial | N/A |
| Outcomes | #12 | Primary, secondary, and other outcomes, including the specific measurement variable (eg, systolic blood pressure), analysis metric (eg, change from baseline, final value, time to event), method of aggregation (eg, median, proportion), and time point for each outcome. Explanation of the clinical relevance of chosen efficacy and harm outcomes is strongly recommended | 5, 18 |
| Participant timeline | #13 | Time schedule of enrolment, interventions (including any run-ins and washouts), assessments, and visits for participants. A schematic diagram is highly recommended (see Figure) | 8 |

For peer review only

| | | | |
|---|---|---|---|
| Sample size | #14 | Estimated number of participants needed to achieve study objectives and how it was determined, including clinical and statistical assumptions supporting any sample size calculations | 6 |
| Recruitment | #15 | Strategies for achieving adequate participant enrolment to reach target sample size | |
| **Methods: Assignment of interventions (for controlled trials)** | | | |
| Allocation: sequence generation | #16a | Method of generating the allocation sequence (eg, computer-generated random numbers), and list of any factors for stratification. To reduce predictability of a random sequence, details of any planned restriction (eg, blocking) should be provided in a separate document that is unavailable to those who enrol participants or assign interventions | 9 |
| Allocation concealment mechanism | #16b | Mechanism of implementing the allocation sequence (eg, central telephone; sequentially numbered, opaque, sealed envelopes), describing any steps to conceal the sequence until interventions are assigned | 9 |
| Allocation: implementation | #16c | Who will generate the allocation sequence, who will enrol participants, and who will assign participants to interventions | 9 |

For peer review only

| | | | |
|---|---|---|---|
| Blinding (masking) | #17a | Who will be blinded after assignment to interventions (eg, trial participants, care providers, outcome assessors, data analysts), and how | 9-10 |
| Blinding (masking): emergency unblinding | #17b | If blinded, circumstances under which unblinding is permissible, and procedure for revealing a participant’s allocated intervention during the trial | 9-10 |
| **Methods: Data collection, management, and analysis** | | | |
| Data collection plan | #18a | Plans for assessment and collection of outcome, baseline, and other trial data, including any related processes to promote data quality (eg, duplicate measurements, training of assessors) and a description of study instruments (eg, questionnaires, laboratory tests) along with their reliability and validity, if known. Reference to where data collection forms can be found, if not in the protocol | 15 |
| Data collection plan: retention | #18b | Plans to promote participant retention and complete follow-up, including list of any outcome data to be collected for participants who discontinue or deviate from intervention protocols | 15 |
| Data management | #19 | Plans for data entry, coding, security, and storage, including any related processes to promote data quality (eg, double data entry; range checks for data values). | 20 |

For peer review only

| | | | |
|---|---|---|---|
| | | Reference to where details of data management procedures can be found, if not in the protocol | |
| Statistics: outcomes | #20a | Statistical methods for analysing primary and secondary outcomes. Reference to where other details of the statistical analysis plan can be found, if not in the protocol | 17 |
| Statistics: additional analyses | #20b | Methods for any additional analyses (eg, subgroup and adjusted analyses) | 17 |
| Statistics: analysis population and missing data | #20c | Definition of analysis population relating to protocol non-adherence (eg, as randomised analysis), and any statistical methods to handle missing data (eg, multiple imputation) | 17, 20 |
| **Methods: Monitoring** | | | |
| Data monitoring: formal committee | #21a | Composition of data monitoring committee (DMC); summary of its role and reporting structure; statement of whether it is independent from the sponsor and competing interests; and reference to where further details about its charter can be found, if not in the protocol. Alternatively, an explanation of why a DMC is not needed | N/A |
| Data monitoring: interim analysis | #21b | Description of any interim analyses and stopping guidelines, including who will have access to these interim results and make the final decision to terminate the trial | 18 |
| Harms | #22 | Plans for collecting, assessing, reporting, and managing solicited and spontaneously reported adverse events and other unintended effects of trial interventions or trial | 14 |

For peer review only

| | | | |
|---|---|---|---|
| | | conduct | |
| Auditing | #23 | Frequency and procedures for auditing trial conduct, if any, and whether the process will be independent from investigators and the sponsor | 20 |
| **Ethics and dissemination** | | | |
| Research ethics approval | #24 | Plans for seeking research ethics committee / institutional review board (REC / IRB) approval | 4, 20 |
| Protocol amendments | #25 | Plans for communicating important protocol modifications (eg, changes to eligibility criteria, outcomes, analyses) to relevant parties (eg, investigators, REC / IRBs, trial participants, trial registries, journals, regulators) | 4 |
| Consent or assent | #26a | Who will obtain informed consent or assent from potential trial participants or authorised surrogates, and how (see Item 32) | 9 |
| Consent or assent: ancillary studies | #26b | Additional consent provisions for collection and use of participant data and biological specimens in ancillary studies, if applicable | N/A |
| Confidentiality | #27 | How personal information about potential and enrolled participants will be collected, shared, and maintained in order to protect confidentiality before, during, and after the trial | 14 |
| Declaration of | #28 | Financial and other competing interests for principal | 22 |

For peer review only

| | | | |
|---|---|---|---|
| interests | | investigators for the overall trial and each study site | |
| Data access | #29 | Statement of who will have access to the final trial dataset, and disclosure of contractual agreements that limit such access for investigators | 22 |
| Ancillary and post trial care | #30 | Provisions, if any, for ancillary and post-trial care, and for compensation to those who suffer harm from trial participation | N/A |
| Dissemination policy: trial results | #31a | Plans for investigators and sponsor to communicate trial results to participants, healthcare professionals, the public, and other relevant groups (eg, via publication, reporting in results databases, or other data sharing arrangements), including any publication restrictions | 4 |
| Dissemination policy: authorship | #31b | Authorship eligibility guidelines and any intended use of professional writers | 4 |
| Dissemination policy: reproducible research | #31c | Plans, if any, for granting public access to the full protocol, participant-level dataset, and statistical code | 22 |

**Appendices**

| | | | |
|---|---|---|---|
| Informed consent materials | #32 | Model consent form and other related documentation given to participants and authorised surrogates | |
| Biological specimens | #33 | Plans for collection, laboratory evaluation, and storage of biological specimens for genetic or molecular analysis in the current trial and for future use in ancillary studies, if applicable | N/A |

For peer review only

None The SPIRIT Explanation and Elaboration paper is distributed under the terms of the Creative Commons Attribution License CC-BY-NC. This checklist can be completed online using https://www.goodreports.org/, a tool made by the EQUATOR Network in collaboration with Penelope.ai

For peer review only

BMJ Open

# Using Routine Healthcare Data to evaluate the impact of the Medicines at Transitions Intervention (MaTI) on clinical outcomes of patients hospitalised with heart failure: protocol for the Improving the Safety and Continuity Of Medicines management at Transitions of care (ISCOMAT) cluster randomised controlled trial with embedded process evaluation, health economics evaluation and internal pilot

| | |
|---|---|
| Journal: | *BMJ Open* |
| Manuscript ID | bmjopen-2021-054274.R1 |
| Article Type: | Protocol |
| Date Submitted by the Author: | 24-Feb-2022 |
| Complete List of Authors: | Moreau, Lauren; University of Leeds Clinical Trials Research Unit, CTRU<br>Holloway, Ivana; University of Leeds Clinical Trials Research Unit<br>Fylan, Beth; University of Bradford, School of Pharmacy and Medical Sciences; NIHR Yorkshire and Humber PSTRC, Bradford Insitiue for Health Research<br>Hartley, Suzanne; University of Leeds Clinical Trials Research Unit, Clinical Trials Research Unit<br>Cundill, Bonnie; University of Leeds Clinical Trials Research Unit<br>Fergusson, Alison; University of Leeds Clinical Trials Research Unit<br>Alderson, Sarah; University of Leeds, Academic Unit of Primary Care<br>Alldred, David; University of Leeds, School of Healthcare; NIHR Yorkshire and Humber Patient Safety Translational Research Centre, Bradford Institute for Health Research<br>Bojke, Chris; University of Leeds, Academic Unit of Health Economics<br>Breen, Liz; University of Bradford, School of Pharmacy and Medical Sciences; University of Bradford<br>Ismail, Hanif; University of Bradford, School of Pharmacy and Medical Sciences<br>Gardner, Peter; University of Bradford, School of Pharmacy and Medical Sciences; University of Bradford, Wolfson Centre for Applied Health Research<br>Mason, Ellen; University of Leeds Clinical Trials Research Unit<br>Powell, Catherine; University of Bradford Faculty of Health Studies, Centre for Applied Dementia Studies<br>Silcock, Jonathan; University of Bradford School of Pharmacy and Medical Sciences<br>Taylor, Andrew; ISCOMAT Patient Led Steering Group<br>Farrin, Amanda; University of Leeds Clinical Trials Research Unit<br>Gale, Chris; University of Leeds Leeds Institute of Cardiovascular and Metabolic Medicine; Leeds Teaching Hospitals NHS Trust Department of Cardiology |
| <b>Primary Subject Heading</b>: | Cardiovascular medicine |

| | |
|---|---|
| Secondary Subject Heading: | Research methods |
| Keywords: | Heart failure < CARDIOLOGY, Cardiology < INTERNAL MEDICINE, Clinical trials < THERAPEUTICS |
| | |


SCHOLARONE™
Manuscripts

## Title

Using Routine Healthcare Data to evaluate the impact of the Medicines at Transitions Intervention (MaTI) on clinical outcomes of patients hospitalised with heart failure: protocol for the Improving the Safety and Continuity Of Medicines management at Transitions of care (ISCOMAT) cluster randomised controlled trial with embedded process evaluation, health economics evaluation and internal pilot

## Names of protocol contributors

Lauren Moreau[1], Ivana Holloway[1], Beth Fylan[2, 3], Suzanne Hartley[1], Bonnie Cundill[1], Alison Fergusson[1], Sarah Alderson[4], David Alldred[3, 4], Chris Bojke[5], Liz Breen[2, 3], Hanif Ismail[2], Peter Gardner[2], Ellen Mason[1], Catherine Powell[2], Jon Silcock[2, 3], Andrew Taylor[6], Amanda J. Farrin[1], Chris P. Gale[7]. On behalf of the ISCOMAT Programme Management Team

[1]Leeds Institute for Clinical Trials Research, Clinical Trials Research Unit, University of Leeds, Leeds, UK LS2 9JT

[2]School of Pharmacy and Medical Sciences, Faculty of Life Sciences, University of Bradford, Bradford, UK; Wolfson Centre for Applied Health Research, Bradford, UK

[3]NIHR Yorkshire and Humber Patient Safety Translational Research Centre, Bradford, UK

[4]School of Healthcare, University of Leeds, Leeds, UK

[5]Academic Unit for Health Economics, University of Leeds, Leeds UK LS2 9JT

[6] ISCOMAT Patient Led Steering Group, Manchester UK

[7]Leeds Institute of Cardiovascular and Metabolic Medicine, University of Leeds, Leeds, UK; Leeds Institute for Data Analytics, University of Leeds, Leeds, UK; Department of Cardiology, Leeds Teaching Hospitals NHS Trust, Leeds, UK

Lauren Moreau (corresponding author), Leeds Institute for Clinical Trials Research, Clinical Trials Research Unit, University of Leeds, Leeds, UK LS2 9JT; 0113 343 7588; l.a.moreau@leeds.ac.uk

Ivana Holloway, Leeds Institute for Clinical Trials Research, Clinical Trials Research Unit, University of Leeds, Leeds, UK LS2 9JT; I.Holloway@leeds.ac.uk

Beth Fylan, School of Pharmacy and Medical Sciences, Faculty of Life Sciences, University of Bradford, Bradford, UK; Wolfson Centre for Applied Health Research, Bradford, UK; B.Fylan@bradford.ac.uk

Suzanne Hartley, Leeds Institute for Clinical Trials Research, Clinical Trials Research Unit, University of Leeds, Leeds, UK LS2 9JT; S.Hartley@leeds.ac.uk

Bonnie Cundill, Leeds Institute for Clinical Trials Research, Clinical Trials Research Unit,

For peer review only



For peer review only - http://bmjopen.bmj.com/site/about/guidelines.xhtml

University of Leeds, Leeds, UK LS2 9JT; B.E.Cundill@leeds.ac.uk
Alison Fergusson, Leeds Institute for Clinical Trials Research, Clinical Trials Research Unit, University of Leeds, Leeds, UK LS2 9JT; A.Fergusson@leeds.ac.uk
Sarah Alderson, Leeds Institute for Health Sciences, University of Leeds, Leeds, UK LS2 9JT; S.L.Alderson@leeds.ac.uk
David Alldred, Wolfson Centre for Applied Health Research, Bradford, UK; Bradford Institute for Health Research, NIHR Yorkshire and Humber Patient Safety Translational Research Centre, Bradford, UK; School of Healthcare, University of Leeds, Leeds, UK; D.P.Alldred@leeds.ac.uk
Chris Bojke, Academic Unit for Health Economics, University of Leeds, Leeds UK LS2 9JT; C.Bojke@leeds.ac.uk
Liz Breen School of Pharmacy and Medical Sciences, Faculty of Life Sciences, University of Bradford, Bradford, UK; Wolfson Centre for Applied Health Research, Bradford, UK; Bradford Institute for Health Research, NIHR Yorkshire and Humber Patient Safety Translational Research Centre, Bradford, UK; L.Breen@bradford.ac.uk
Hanif Ismail School of Pharmacy and Medical Sciences, Faculty of Life Sciences, University of Bradford, Bradford, UK; Wolfson Centre for Applied Health Research, Bradford, UK; H.Ismail11@bradford.ac.uk
Peter Gardner School of Pharmacy and Medical Sciences, Faculty of Life Sciences, University of Bradford, Bradford, UK; Wolfson Centre for Applied Health Research, Bradford, UK; P.H.Gardner@bradford.ac.uk
Ellen Mason, Leeds Institute for Clinical Trials Research, Clinical Trials Research Unit, University of Leeds, Leeds, UK LS2 9JT; E.Mason1@leeds.ac.uk
Catherine Powell School of Pharmacy and Medical Sciences, Faculty of Life Sciences, University of Bradford, Bradford, UK; Wolfson Centre for Applied Health Research, Bradford, UK; C.Powell2@bradford.ac.uk
Jon Silcock School of Pharmacy and Medical Sciences, Faculty of Life Sciences, University of Bradford, Bradford, UK; Wolfson Centre for Applied Health Research, Bradford, UK; J.Silcock@bradford.ac.uk
Andrew Taylor, ISCOMAT Patient Led Steering Group, Manchester UK; ataylor102@outlook.com
Amanda J. Farrin Leeds Institute for Clinical Trials Research, Clinical Trials Research Unit, University of Leeds, Leeds, UK LS2 9JT; A.J.Farrin@leeds.ac.uk
Chris P. Gale Leeds Institute of Cardiovascular and Metabolic Medicine, University of Leeds, Leeds, UK; Leeds Institute for Data Analytics, University of Leeds, Leeds, UK; Department of Cardiology, Leeds Teaching Hospitals NHS Trust, Leeds, UK; c.p.gale@leeds.ac.uk

**Word count: 4016**


For peer review only - http://bmjopen.bmj.com/site/about/guidelines.xhtml

## Abstract

### Introduction

Heart failure affects 26 million people globally, approximately 900 thousand people in the UK, and is increasing in incidence. Appropriate management of medicines for heart failure at the time of hospital discharge reduces readmissions, improves quality of life and increases survival. The ISCOMAT trial tests the effectiveness of the Medicines at Transition Intervention (MaTI) which aims to enhance self-care and increase community pharmacy involvement in the medicines management of heart failure patients.

### Methods and analysis

ISCOMAT is a parallel-group cluster randomised controlled trial, randomising 42 National Health Service trusts with cardiology wards in England on a 1:1 basis to implement the MaTI or treatment as usual. Around 2100 patients over the age of 18, admitted to hospital with heart failure with at least moderate left ventricular systolic dysfunction within the last 5 years, and planned discharge to the geographical area of the cluster will be recruited. The MaTI consists of training for staff, a toolkit for participants, transfer of discharge information to community pharmacies, and a medicines reconciliation/review. Treatment as usual is determined by local policy and practices. The primary outcome is a composite of all-cause mortality and heart failure-related hospitalisation at 12 months post-registration obtained from national electronic health records. The key secondary outcome is continued prescription of guideline-indicated therapies at 12-months measured via patient-reported data and Hospital Episode Statistics. The trial contains a parallel mixed-methods process evaluation and an embedded health economics study.

### Ethics and dissemination

The study obtained approval from the Yorkshire and the Humber – Bradford Leeds Research Ethics Committee; REC reference 18/YH/0017. Findings will be disseminated via academic and policy conferences, peer-reviewed publications and social media. Amendments to the protocol are disseminated to all relevant parties as required.



### Registration

ISRCTN66212970

### Key words

Heart failure; cardiology; clinical trials; cluster trial; medicines




For peer review only - http://bmjopen.bmj.com/site/about/guidelines.xhtml

## Article summary

### Strengths and limitations of this study

- ISCOMAT is a trial which evaluates a process to optimise medicines use for patients discharged from hospital with heart failure to their usual place of residence
- The trial intervention was developed through experience-based co-design with patients and health care professionals
- The trial will use nationwide patient-level health records for the primary endpoint
- Due to intervention implementation requirements, patients were recruited from designated cardiology wards only
- Clinical events cannot be centrally adjudicated as they arise from electronic health records. As such this is a pragmatic trial with results generalizable to the ‘real world’ environment.

For peer review only


For peer review only - http://bmjopen.bmj.com/site/about/guidelines.xhtml

## Background

Heart failure affects 26 million people globally,[1] approximately 900 thousand people in the UK, and is increasing in incidence.[2] People with heart failure frequently use multiple healthcare services,[3] so the economic burden of heart failure is substantial and is further compounded by high rates of hospitalisation and subsequent readmission.[4]

Heart failure may be managed through a combination of pharmacological treatments at titrated doses including angiotensin-converting enzyme (ACE) inhibitors, beta-adrenoceptor antagonists (beta-blockers), mineralocorticoid/aldosterone receptor antagonists (MRA), angiotensin receptor neprilysin inhibitors, sodium-glucose transport protein 2 (SGLT2) inhibitors and diuretics as well as implanted devices.[5] Medicines optimisation reduces hospitalisation, improves quality of life and increases survival rates.[6] However, this requires the coordinated input of several healthcare professionals, following hospitalisation, which may be subject to variation.[7] Medicines management at care transitions is problematic in healthcare systems internationally.[8] Estimated readmissions rate within three months of discharge for patients with heart failure are as high as 50%[9], indicating a need to achieve improvements in the continuity of care at the time of transfer to the community.

The Medicines at Transitions Intervention (MaTI) was designed to improve the use of prescribed medicines when patients with heart failure are discharged from hospital. It was developed through qualitative research and co-design with patients and healthcare staff.[7 10] Intervention feasibility was tested in three healthcare areas with 31 participants in northern England in 2017.[11] Following the feasibility study, the intervention was refined before progressing to the main trial. [11] Following Medical Research Council guidance for the design and evaluation of complex interventions,[12] we report the protocol of the ISCOMAT trial, a cluster randomised controlled trial to evaluate the clinical and cost-effectiveness of the MaTI among heart failure patients.

## Methods and design

## Aim and Objectives

The overall aim of ISCOMAT (ISRCTN Registration 66212970) is to evaluate the effectiveness of the MaTI on the delivery of guideline recommended care and clinical outcomes for patients who are discharged from hospital with heart failure, when compared with treatment as usual.

The primary objective is to establish whether the MaTI reduces all-cause mortality and heart failure rehospitalisation measured over 12 months from registration.

The key secondary objective is to determine whether the intervention increases the proportion of participants being prescribed the composite of guideline-indicated therapies at 12 months post-


For peer review only - http://bmjopen.bmj.com/site/about/guidelines.xhtml

registration (i.e. ACE inhibitor, angiotensin II receptor blocker (ARB) or salcubitril/valsartan, beta blocker and/or ivabradine and MRA).[13]

Other secondary objectives, measured at 12-months post-registration unless stated otherwise, are to establish if the intervention:

- Increases time to all-cause mortality
- Increases time to heart failure-related rehospitalisation
- Increases time on guideline recommended (and indicated as above) cardiovascular medicines.
- Improves patient understanding of their medicines and satisfaction with medicines-related care at 2- and 6-week post-discharge and 12-months post-registration
- Increases number of days alive and out of hospital
- Reduces hospitalisations (all cause, cardiovascular related and heart failure)
- Reduces mortality due to specific causes.
- Is cost-effective

The internal pilot objective is to assess whether the trial recruitment meets the pre-defined progression criteria thresholds.

The objectives of the mixed-methods process evaluation are to determine intervention fidelity; the relationship between implementation/fidelity and outcomes; and the barriers or facilitators to the successful implementation and roll out of the intervention.[14]

## Trial Design

This publication describes REC approved Study Protocol v4.0 and dated 08 August 2018.

ISCOMAT is a parallel-group cluster randomised controlled trial that aims to recruit 2100 participants hospitalised with heart failure from 42 National Health Service (NHS) trusts in England (approximately 50 participants per site) over a 12-month recruitment period. Clusters are defined as a distinct geographical area and consist of the NHS trust with a cardiology ward(s) and/or a Coronary Care Unit (CCU; defined as a ward with nursing staff with specific cardiac nursing expertise) together with associated community pharmacies within the Clinical Commissioning Group boundaries in that geographical area (Figure 1).

For peer review only


For peer review only - http://bmjopen.bmj.com/site/about/guidelines.xhtml

ISCOMAT was purposively designed to utilise the routine data sources available through providers such as NHS Digital, the Office for National Statistics and the National Heart Failure Audit. The use of routine health records data has enabled the trial to reduce the burden of data collection on research teams and patients and ensure a high level of accuracy with minimal data loss for the primary endpoint.

***[Insert** Figure 1: Trial process diagram here]*

For peer review only



For peer review only - http://bmjopen.bmj.com/site/about/guidelines.xhtml

**Cluster eligibility**

Acute NHS Trusts with accompanying cardiology wards that met all of the inclusion criteria were eligible to take part in the study as described in Table 1.

*Table 1: Site Eligibility Criteria*

| **Eligibility Criteria** | **Details** |
|---|---|
| Inclusion | Commitment to achieve the recruitment target |
| | Participation in the National Heart Failure Audit[1] |
| | Hospital Episode Statistics available via NHS Digital |
| | Deemed suitable to implement and deliver the intervention (as determined by the ISCOMAT team).[2] |
| Exclusion | Already providing medicines management deemed to be sufficiently similar to the MaTI |

[1] Participation in UK National Heart Failure Audit was required to facilitate acquiring the dataset from NICOR
[2] Sites were deemed suitable where there was at least one designated cardiology ward and/or CCU and had an existing mechanism to communicate with community pharmacies for all heart failure patients (or were willing to introduce one).

**Randomisation and blinding**

NHS Trusts were required to obtain all necessary local management approvals prior to randomisation and this was used to evidence consent. Randomisation was undertaken independently by the Leeds Clinical Trials Research Unit (CTRU). Clusters were allocated on a 1:1 basis either to implement the MaTI or treatment as usual (TAU) using minimisation with a random element, minimised by:

- geographical region, as defined by National Institute for Health Research Clinical Research Networks;[15]
- type of hospital (university hospital or non-university hospital as defined by membership of the University Hospital Association)[16]; and
- method of clinical handover/information transfer to community pharmacy (electronic system, e.g. PharmOutcomes;[17] or manual system, e.g. standard email, fax, letter or phone).

**Participant eligibility and registration**

Patient recruitment commenced approximately four weeks following cluster randomisation. Potential participants were screened following admission to hospital and recruited prior to discharge. Patient screening, consent, recruitment and baseline data collection was completed by research staff, who had no role in the delivery of care or treatment and, wherever possible, were


For peer review only - http://bmjopen.bmj.com/site/about/guidelines.xhtml

blinded to the cluster allocation to mitigate selection bias.[18] Site staff were asked to self-report instances of unblinding to CTRU. This was monitored at monthly Trial Management Group Meetings (TMG) and annual Trial Steering Committee (TSC) meetings. Consented participants meeting the eligibility criteria described in Table 2 Table 2 are included in the trial.

*Table 2: Participant Eligibility Criteria*

| **Eligibility Criteria** | **Details** |
|---|---|
| Inclusion | Admitted or transferred to a ward participating in the ISCOMAT trial |
| | Heart failure with evidence of at least moderate left ventricular systolic dysfunction* confirmed within the last 5 years. *Defined as: a left ventricular ejection fraction of less than or equal to 44%, quantified via echocardiogram; or the equivalent of this, if quantified by a different imaging modality.* |
| | Aged 18 years or over at hospital admission. |
| | Planned discharged from recruiting hospital to their home (defined by usual place of residence) or a care home. |
| | Planned discharge within geographical area of that cluster |
| | Capacity to provide informed consent |
| | Provide informed consent |
| Exclusion | Patients in a terminal phase of illness / end of life care pathway who were not expected to survive beyond 6 weeks post-discharge. |
| | Patients already participating in ISCOMAT (e.g. re-admissions) |

Documented reasons for ineligibility or declining participation were closely monitored by the trial team as part of a regular review of recruitment progress, to ensure generalisability of study results in accordance with CONSORT reporting guidelines, and to highlight issues in identifying or recruiting patients during the internal pilot.

Following confirmation of written informed consent and eligibility, participants were registered into the trial by an authorised member of staff at the trial research site, via a central automated 24-hour online registration system hosted at CTRU.


For peer review only - http://bmjopen.bmj.com/site/about/guidelines.xhtml
For peer review only

## Trial status

The first trial site was randomised on 16th April 2018, opened to recruitment on 1st June 2018 and the first participant was recruited on 8th June 2018. A total of 44 clusters were randomised, of which 43 opened to recruitment and registered at least one participant. Overall 1641 participants consented to take part. Recruitment was due to end on 31st December 2019 but was extended to 31st December 2020. Following discussion with the sponsor and in line with government guidance, recruitment at all sites paused on 18th March 2020 due to the COVID-19 pandemic and following consultation with the TSC/Programme Steering Committee (PSC)/funder the decision was taken to close ISCOMAT to recruitment on 28th July 2020.

## Governance

The trial is funded by a National Institute for Health Research (NIHR) Programme Grant for Applied Research (Reference: RP-PG-0514-20009. The views expressed in this paper are those of the authors and not necessarily those of the NIHR or the Department of Health and Social Care. The trial is sponsored by Bradford Teaching Hospitals NHS Foundation Trust.

## Intervention

The MaTI was designed to be implemented by differently specialised professional clinical teams, including nurses and pharmacists. The intervention was initiated by the discharge nurse (or equivalent) and continued post-discharge through involvement of the community pharmacist. MaTI comprises of:

1) Patient-held personalisable medicines toolkit containing: information about heart failure, medicines and their effects; details of the patients' healthcare team; a checklist of medicines-related activity that should occur in hospital before discharge, at discharge and once the patient has returned home; a traffic-light guide to symptoms plus actions to take if symptoms worsen. This will be delivered to patients face-to-face by a nurse of pharmacist from the cardiology ward as part of routine care at intervention sites. See supplementary files MATI form A and B for example checklists completed at site.
2) Enhanced communication between hospitals and the patients' community pharmacists through transfer of the patient's discharge letter and medicines list to community pharmacy, with a request for the community pharmacist to reconcile the patient's medicines and invite the patient for a medicines use review (MUR) or a discussion about their medicines. See supplementary file of Community pharmacy cover letter.

The intervention was delivered to all eligible patients as part of routine care. Clinical staff were not aware which patients have consented to data collection and evaluation.

For peer review only


For peer review only - http://bmjopen.bmj.com/site/about/guidelines.xhtml

**Intervention Training**

Clusters randomised to the intervention were asked to identify key members of the clinical team involved in medicines management (e.g. ward nurses, hospital pharmacists, primary care heart failure nurses) to complete intervention training online and in-person. The intervention team aimed to train around 5-7 staff members in each hospital site to enable the site coordinator and nominated staff to brief others about the MaTI. The online training was developed with the Centre for Pharmacy Postgraduate Education (www.cppe.ac.uk) to enable staff to gain an understanding of the MaTI. Face-to-face training explained the background to MaTI (importance, purpose, design, outcomes) and went through the supporting materials/ guides designed to aid facilitation of delivery. Approximately one month was allowed in each cluster for training and embedding the MaTI into routine practice prior to study recruitment commencing. Each cluster identified one member of staff to adopt the 'site coordinator' role, responsible for ensuring the intervention was implemented according to the guidance and training provided. The number and role of hospital staff trained in each cluster was dependent on the structure of cardiology/pharmacy services and available staff. After 3-4 weeks, further follow up visits were arranged to ensure adherence to the protocol.

Staff delivery of the intervention on an individual level was communicated to intervention team regularly to support ongoing implementation of the intervention. Additional support and training were available when required.

**Measuring adherence to the intervention**

The TMG monitored adherence to intervention protocols every month by reviewing delivery of key intervention components across all intervention sites. Performance feedback was provided to site teams via monthly emails. Data on adherence was also reviewed at annual TSC meetings.

**Treatment as usual**

Treatment as usual was determined by local Trust policies and practices. Variations in treatment as usual were expected across NHS cardiology services in England and documented in site feasibility assessments, during recruitment, and at the end of follow-up.

**Process Evaluation**

A parallel embedded process evaluation aims to inform the trial findings and aid potential future wider scale implementation. The process evaluation involved six purposively selected intervention sites using a mixed-methods design. Fidelity and barriers/enablers of implementation of MaTI were explored using observation, interviews of up to 20 patients and 40 healthcare professionals, staff


For peer review only - http://bmjopen.bmj.com/site/about/guidelines.xhtml
For peer review only

surveys and trial-specific data collection on MaTI adherence.[14] A parallel mixed analysis is planned. Qualitative data is thematically analysed using Framework analysis and survey data is analysed using descriptive statistics. Data is synthesised, triangulated and mapped to the Consolidated Framework for Implementation Research (CFIR)[19 20] where appropriate. A full description of methods are published in Powell et al.[14]

**Outcome measures**

The primary outcome is the binary composite endpoint of all-cause mortality and heart failure-related hospitalisation within 12 months post-registration (see Box 1 for definition of hospitalisation). Mortality data is obtained from the Civil Registration Death data from the Office for National Statistics, and hospitalisation data from Hospital Episode Statistics (HES)[21] from NHS Digital. Routine data for the analysis of primary and secondary outcomes is supported by site reported and patient reported data where appropriate.

Box 1: Definition of hospitalisation

| |
|---|
| **Definition of hospitalisation:** Hospitalisation is defined as an acute inpatient admission (regardless of length of stay). A hospitalisation will be considered heart-failure related if the primary reason / diagnosis for admission included ICD-10 codes or procedural classifications related to heart failure |

The key secondary outcome is participants still being prescribed at least one of the guideline recommended therapies at 12 months in each of the following three groups:

- ACE inhibitor (ACEI); angiotensin II receptor blocker (ARB); salcubitril/valsartan
- Beta blocker; ivabradine
- Mineralocorticoid receptor antagonist (MRA)

For patients with contraindications to any of the three groups, the endpoint will be derived with respect to groups that are indicated. Data is collected via patient-reported questionnaires collected at baseline, 3-month and 6-month post-registration. We also plan to obtain data from the NHS Business Service Authority on medicines dispensed in primary care, via NHS Digital.

The following secondary outcomes are collected over the 12-month post-registration via routine data sources:

- Individual components of the primary endpoint regarded as time to event endpoints. Time will be measured from registration to the first day of heart failure rehospitalisation or death
- Length of time on guideline recommended cardiovascular medications
- Days alive and out of hospital: defined as the number of days in the year beginning the day after registration that the patient spends alive and not in hospital


For peer review only - http://bmjopen.bmj.com/site/about/guidelines.xhtml

- Time to cardiovascular-related hospitalisation and all cause hospitalisation
- Mortality due to specific causes
- Healthcare resource use

The following secondary outcomes are measured at baseline, and 3-month and 12-month post-registration unless stated otherwise:

- Patient satisfaction with their medicines care and patient understanding of their medicines; measured via a patient experience survey at 2 and 6-weeks post-discharge, and 12-months post-registration with a 10-point Likert scale. For intervention participants data on the use of the MaTI over a 12-month post-registration period is also captured.
- EuroQol5-Dimension Health Questionnaire (3 levels) (EQ-5D-3L)[22]; measures health utility (quality of life) comprising five dimensions: mobility, self-care, usual activities, pain/discomfort and anxiety/depression. Dimension responses are combined and converted into a summary index (0 for dead, 1 for perfect health and negative values for states worse than death).
- Healthcare resource use: additional to routine datasets, we capture patient reported healthcare resource use using standard self-reported resource use questionnaires, to include hospital attendances, GP, nurse, specialist heart failure nurse and pharmacy contact.

**Data collection**

The outcome assessment schedule is outlined in Table 3**Error! Reference source not found.**. All personal information is processed in accordance with the Data Protection Act 2018[23] (and any successor legislation). Appropriate storage, restricted access and disposal arrangements for personal and clinical details are in place. Archiving is for a minimum of 10 years.

*Routine Data*

ISCOMAT will obtain data from routine providers NHS Digital (including HES and ONS data) and NICOR and the UK National Heart Failure Audit for the primary and secondary outcomes as outlined above. The trial was designed to use data from national routine sources to maximise efficiency in answering the research questions, ensure consistency across multiple sites, and minimise burden on patients and research staff at hospitals.

*Clinical Data*

Recruiting teams collected information on study eligibility and details of the participants' admission and discharge, including heart failure history, clinical details, primary aetiology for heart failure,


For peer review only - http://bmjopen.bmj.com/site/about/guidelines.xhtml

detailed medicines information, and related unexpected serious adverse events (RUSAEs). All-cause mortality and re-admission data were collected throughout the study up until 12 months post registration from hospital sites and electronic health records (NHS Digital). Consent was sought to obtain data from electronic health records – including secondary care records and NHS Digital.

*Participant reported data*

Patient self-reported baseline measures were collected in hospital inpatient setting. Patient reported outcomes at 2- and 6-week post discharge, and 3- and 12-months post registration were collected via post. When questionnaires were not returned, the CTRU sent postal reminders.

*Intervention fidelity data and quantitative and qualitative data from observations, participant and staff interviews and surveys*

Clinical teams, randomised to the intervention, returned a checklist of seven steps for each trial participant, confirming completion of intervention activity and local pharmacy information. The CTRU sought data from the participants' community pharmacy following discharge. Details about the heart failure service models and the clinical role of those staff responsible for the delivery of the MaTI were documented.

*Table 3: Schedule of enrolment, interventions and assessments for participants*

| Assessment | Type | Method of Completion | Timepoint | | | | | |
|---|---|---|---|---|---|---|---|---|
| | | | Screening | Baseline | 2 weeks post-discharge | 6 weeks post-discharge | 3 months post-registration | 12 months post-registration |
| PARTICIPANT DATA | | | | | | | | |
| Screening (demographics / assessment of eligibility) | CRF[1] | CRN[2] / local research staff | X | | | | | |
| Consent | Consent Form | Self-completion | | X | | | | |
| Eligibility | CRF | CRN / local research staff | | X | | | | |
| Demographics | CRF | CRN / local research staff | | X | | | | |
| Contact Details | CRF | CRN / local research staff | | X | | | | |
| Admission/discharge details | CRF | CRN / local research staff | | X | | | | |


For peer review only - http://bmjopen.bmj.com/site/about/guidelines.xhtml

| | | | | | | | | |
|---|---|---|---|---|---|---|---|---|
| Health Related Quality of Life (EuroQol EQ-5D-3L) | Questionnaire booklet (post) | Self-completion | | X | | | X | X |
| Healthcare Resource Use (in last 3 months) | Questionnaire booklet (post) | Self-completion | | X | | | X | X |
| Current heart failure medications | Questionnaire booklet (post) | Self-completion | | | | | X | X |
| Patient Experience Survey | Questionnaire booklet (post) | Self-completion | | | X | X | | X |
| Primary outcome (all-cause mortality and HF rehospitalisation) | Routine data / CRF | Data Access Request: Routine data providers > CTRU[3] | | DATA EXTRACTS TO BE AGREED WITH DATA PROVIDERS TO ALLOW 12 MONTH DATA COLLECTION | | | | |
| Secondary outcomes | Routine data / CRF | Data Access Request: Routine data providers > CTRU | | DATA EXTRACTS TO BE AGREED WITH DATA PROVIDERS TO ALLOW 12 MONTH DATA COLLECTION | | | | |
| Patient status | CRF | CTRU | | ONGOING | | | | |

| SITE DATA | | | | | |
|---|---|---|---|---|---|
| Assessment | Type | Method of Completion | Baseline | 6 months post-randomisation | 12 months post-implementation of intervention |
| Site questionnaire | Questionnaire Booklet | CTRU | X | X | X |

| INTERVENTION DATA | | | | |
|---|---|---|---|---|
| Assessment | Type | Method of Completion | 3 months post-registration | 6 months post-implementation of intervention |
| Adherence / fidelity data (collection of patient checklists to confirm what happened at discharge, MUR review confirmation from the pharmacy) | CRF | CTRU | ONGOING | |
| Interviews - Patients | Process Evaluation | Research Fellow | X | |
| Interviews - Staff | Process Evaluation | Research Fellow | | x |
| Survey - Staff | Process Evaluation | Research Fellow | | x |
| Observations - ward | Process Evaluation | Research Fellow | | x |

[1]Case report form (CRF)
[2]Clinical Research Network (CRN)
[3]Clinical Trials Research Unit (CTRU)

**Statistical methods**


For peer review only - http://bmjopen.bmj.com/site/about/guidelines.xhtml

## Sample size

Sample size calculations were based on previous research[24 25] indicating that the combined primary event rate is likely to be at least 20% in control sites. With 42 clusters, an estimated intracluster correlation coefficient (ICC) of 0.01, a coefficient of variation in cluster size of 0.23, and 15% loss to follow-up (based on studies in this patient group),[26] 50 patients with heart failure in each cluster (2100 in total) are required to provide at least 80% power at 5% significance level to detect a minimal clinically important difference (MCID) of 6%.[26] The MaTI is delivered to each heart failure participant by dispersed teams across the care transition (secondary-care, primary-care, community pharmacy), therefore, the anticipated ICC is likely to be relatively low.

This sample size also provides power in excess of 90% to detect a MCID of 12% in the key secondary outcome even with the most conservative assumption for the control rate.

## Internal pilot

Descriptive statistics were used to evaluate progression criteria assessing site-level recruitment rates. This analysis informed study continuation beyond the internal pilot phase. The progression criterion was assessed at 6-months after recruitment commenced, based on the following traffic light system of green (go), amber (review) and red (stop):

Green: ≥4 patients/month/NHS trust;

Amber: ≥2 but <4 patients/month/NHS trust;

Red: <2 patients/month/NHS trust.

## Data analysis

A full statistical analysis plan (SAP) will be finalised prior to any analyses. The CTRU will conduct the statistical analysis and data summaries on an intention-to-treat (ITT) population, which is defined as all clusters randomised and all participants registered regardless of non-compliance with the protocol or withdrawal from the study. The study is conducted and will be reported according to the CONSORT extensions for cluster trials[27] and CONSORT-ROUTINE[28] for trials conducted using cohorts and routinely collected data. Baseline data will be summarised by treatment group and at the participant level and/or the cluster level using descriptive statistics as appropriate.


For peer review only - http://bmjopen.bmj.com/site/about/guidelines.xhtml
For peer review only

Additionally, the SAP will describe the derivation of outcomes and detail the analyses for each outcome measure. The analyses models will reflect clustering and include fixed effects for the minimisation factors with clusters as random effects, see Table 4.

A complier average causal effect (CACE) analysis approach to estimate the treatment effect for the primary outcome amongst compliant clusters will be considered depending on levels of adherence to the intervention as intended. Definitions of adherence and pre-defined thresholds will be outlined in the SAP and made without reference to the effectiveness data.

No formal interim analyses were planned. Blinded interim reports were presented to the TSC containing descriptive information. This included data on recruitment, follow-up, adherence, safety and data quality. These were presented by randomised arm if specifically requested as such, with treatments masked. As participants were recruited following cluster randomisation, monitoring of baseline characteristics by arm are presented to assess post-randomisation selection bias. A single final analysis is planned when all follow-up data has been entered onto trial database, and the database is cleaned and locked.

In the cost-effectiveness analysis, the primary outcome is Quality Adjusted Life Years (QALYs) compared to costs from an NHS and PSS perspective. A secondary cost effectiveness analysis will look at the incremental cost per all-cause deaths prevented and re-hospitalisation prevented. Resource use associated with 'treatment as usual' and the intervention will be collected through the health resource use questionnaire administered to patients. Prescription and hospitalisation data will be collected from the data linkage outputs and supported where necessary by the patient health resource use questionnaire at 3 & 12 months. Unit costs for health service resources will be obtained from national sources (e.g. Personal Social Services Research Unit [PSSRU] British National Formulary for medicines). The intervention cost will include any enhancement of existing medicines management components and any 'new' components of the MaTI (e. g. the staff training module or necessary enhancements to existing community pharmacy medicines services). We will assess uncertainty using a within trial probabilistic sensitivity analysis undertaken using Monte Carlo simulation with results presented as Incremental Cost-Effectiveness rations (ICERs) and cost effectiveness acceptability curves.

For further detail on outcome measures, see Table 4.

*Table 4: Data analysis plan for primary and secondary outcomes and health economic analysis*

| Analysis activities | Assessed At | Statistical methods |
|---|---|---|


For peer review only - http://bmjopen.bmj.com/site/about/guidelines.xhtml

| | | |
|---|---|---|
| **Primary Endpoint – all-cause mortality and heart failure-related rehospitalisation within 12 months post-registration** | 12 months post registration | The primary endpoint will be analysed using a two-level logistic regression model with participants nested within clusters, with clusters treated as random effects. The model will be adjusted for the following fixed effects: the cluster level stratification variables (level 2): geographical region, type of hospital and method of clinical handover/information transfer to community pharmacy, and patient level covariates (level 1) and other relevant known predictors of outcome. Results will be expressed as point estimates, with corresponding 95% CIs, p-values. An estimate of the ICC and corresponding CI will also be presented. |
| **Key secondary endpoint** | 12 months post-registration | This endpoint will be analysed using a similar modelling strategy as the primary endpoint. |
| **Other secondary endpoints** | 12 months post-registration | Time-to-event analyses will be by multi-level shared frailty models with the event right-censored at either 12 months follow-up, date of death or date of withdrawal, whichever is earliest.<br><br>Days alive and out of hospital will be analysed using mixed effects Poisson regression model.[29] with data censored at either 12-months post-registration, death or withdrawal, whichever is earliest.<br><br>Length of time on guideline recommended cardiovascular medications and cause specific death endpoints will be summarized descriptively. |
| | 2 & 6 weeks post-discharge; 12 months post-registration | Patient understanding of their medicines and satisfaction with medicines-related care will be analysed using Ordinal logistic regression with clustering model to compare participant responses between treatment arms. |
| **Cost-effectiveness analysis** | 12 months post-registration | The primary outcome will be Quality Adjusted Life Years (QALYs). A secondary cost effectiveness analysis will look at the incremental cost per all-cause deaths prevented and re-hospitalisation prevented.<br><br>In order to assess the long-term cost-effectiveness of the intervention, compared to treatment as usual, a decision analytic cost-effectiveness model will be used to estimate the expected incremental cost per QALY. The model will include a Value of Information Analysis (VOIA) to assess the value of undertaking further research to reduce decision uncertainty in the model. The perspective will be the same as the within-trial analysis but the time horizon will be the lifetime of the individual to capture the full impact of any mortality differences on the long-term cost-effectiveness. |


For peer review only - http://bmjopen.bmj.com/site/about/guidelines.xhtml

| | | |
|---|---|---|
| | | Estimation of health-related quality of life will use QALYs and discounting will be at the same rate as for the within trial analysis. Parameter uncertainty will be addressed using probabilistic sensitivity analysis and running Monte Carlo simulations. Results will meet international CHEERS standards of reporting and results will be presented as Incremental Cost-Effectiveness rations (ICERs), Incremental Net Health and Monetary Benefits and Cost Effectiveness Acceptability Curves (CEACs). |

**Data monitoring**

Data is monitored for quality and completeness by the Clinical Trials Research Unit (CTRU) using established verification, validation and checking processes. The Trial Management Group (TMG) comprising the chief investigator (CI), CTRU team, other co-applicants and PPI representative will be responsible for the on-going management of the trial. The TMG is responsible for any audits for trial conduct required. The TMG reports to the Trial Steering Committee (TSC) annually. The TSC is formed with an independent chair, health economist and statisticians.

**Methods to handle missing data**

Missing data are expected and the proportion of missing data will be compared between intervention and control groups. Sensitivity analyses of the primary endpoint will be conducted to assess the impact of missing data, the choice of imputation model and assumptions around the missingness mechanism, as appropriate.

**Ethics and dissemination**

The trial received favourable REC opinion from Yorkshire and the Humber – Bradford Leeds Health Research Ethics Committee on 30/01/2018 with reference number 18/YH/0017
The trial was registered (ISRCTN66212970).

The trial is sponsored by Bradford Teaching Hospitals NHS Foundation Trust, coordinated by the CTRU at the University of Leeds and supported by a TMG and Programme Management Group (PMG) TSC. The TSC includes independent external experts who provide overall supervision for a trial on behalf of the Trial Sponsor and Trial Funder.

This longstanding and ongoing engagement with stakeholders provides a direct pathway to impact for the outputs of this research. Our Patient-Led Steering Group (PLSG) will inform our dissemination strategy and its members will play an active role in the format and content of academic papers


For peer review only - http://bmjopen.bmj.com/site/about/guidelines.xhtml

(specifically patient implications) and will present at local, regional and national conferences and meetings.

**Patient and public involvement**

ISCOMAT has a Patient-Led Steering Group that has contributed throughout the programme. Patient representatives have membership of the TMG, Programme Management Group and TSC. The PLSG has played an integral role in several aspects of the study, including intervention development, the development of data collection tools and the analysis of qualitative interview data. Members of the group have also served as co-authors in programme and trial publications.

**COVID-19 pandemic and considerations for trial closure**

Sites had been brought on gradually in waves across a two-year site set-up period. Sites also closed at various points throughout this period due to reaching or exceeding site-specific targets sample sizes or due to changes in capacity to deliver the intervention. Prior to the onset of the COVID-19 pandemic, we had needed to extendrecruitment for a minimum of one year in order to try and achieve target sample size. A full report on the recruitment rate and generalizability of results will be available in the trial results paper that will be published on completion of trial analysis.

The COVID-19 adversely affected routine NHS services, and there was a decline in admissions with cardiovascular disease.[30 31] Following national NIHR guidance, sponsor decision and TSC review, the ISCOMAT trial was paused in March 2020 and formally closed to recruitment in July 2020. Uncertainty about the post-COVID landscape raised concerns for intervention relevance and deliverability, future research capacity and participant willingness to be involved in research, number of admissions and organisation of heart failure services in sites, plus external policy changes, such as medicines use reviews (MURs). Additional concerns included the effect of site withdrawals following a restart on the trial design and increased post-COVID death rates on our findings. The decision to close the trial was supported by statistical review that concluded that closing recruitment would not have a significant impact on the statistical power or analysis of the primary and key secondary outcomes, given on the proportion of the target sample size recruited to date. Furthermore, this design maximizing routine data sources helped to minimise the impact of COVID-19 on trial analysis plans.

**Conclusion**


For peer review only - http://bmjopen.bmj.com/site/about/guidelines.xhtml

Heart failure outcomes, such as mortality and hospital readmission can be improved though the optimal management of medicines. Optimising treatment after transitions of care requires the coordinated input of different healthcare organisations and professions, and patients.

ISCOMAT is a pragmatic multi-centre cluster RCT that aims to recruit 2100 participants hospitalised with heart failure from across 42 hospitals, approximately 50 participants per site, over a 12-month recruitment period. The ISCOMAT trial will establish if a complex intervention comprising enhanced and personalised communication for patients about their medicines and the transfer of discharge information to the patients' community pharmacy will reduce hospital admissions and mortality over a 12-month period. As such, ISOMAT has the potential to improve the safety and continuity of medicines at care transitions for hospitalised patients with heart failure, and reduce mortality and subsequent hospitalisations.

**Acknowledgements**

We would like to thank Alison Blenkinsopp, Gerry Armitage, Theo Rayner, Ian Kellar, Jan Speechley, the ISCOMAT Patient-led Steering Group, the ISCOMAT Trial Management Group, Trial Steering Committee and Programme Steering Committee.

**Author contributions**

IH, BF, SH, BC, AF, SA, DA, CB, LB, HI, EM, PG, CP, JS, AT, AJF, CPG developed the methodology and detail of the ISCOMAT trial protocol. LM drafted the manuscript, with all authors reviewing the draft critically for intellectual content. All authors approved the final version submitted for publication.

**Funding Statement**

The study was funded as part of a National Institute for Health Research Programme Grant for Applied Research (RP-PG-0514-20009). The views expressed in this paper are those of the authors and not necessarily those of the NHS, the NIHR or the Department of Health.

**Trial Sponsor**

The trial is sponsored by Bradford Teaching Hospitals NHS Foundation Trust.

**Data statement**

For peer review only



For peer review only - http://bmjopen.bmj.com/site/about/guidelines.xhtml

All data requests should be submitted to the corresponding author for consideration. Access to anonymised data may be granted following review.

**Disclaimer**

The views expressed are those of the authors and not necessarily those of the NIHR.

**Competing interests Statement.**

None declared.

For peer review only


For peer review only - http://bmjopen.bmj.com/site/about/guidelines.xhtml

## References

1. Ambrosy AP, Fonarow GC, Butler J, et al. The Global Health and Economic Burden of Hospitalizations for Heart Failure: Lessons Learned From Hospitalized Heart Failure Registries. *Journal of the American College of Cardiology* 2014;63(12):1123-33. doi: https://doi.org/10.1016/j.jacc.2013.11.053
2. Conrad N, Judge A, Tran J, et al. Temporal trends and patterns in heart failure incidence: a population-based study of 4 million individuals. *The Lancet* 2018;391(10120):572-80. doi: 10.1016/S0140-6736(17)32520-5
3. Kim D, Hayhoe B, Aylin P, et al. Health service use by patients with heart failure living in a community setting: a cross-sectional analysis in North West London. *British Journal of General Practice* 2020;70(697):e563. doi: 10.3399/bjgp20X711749
4. Shafie AA, Tan YP, Ng CH. Systematic review of economic burden of heart failure. *Heart Failure Reviews* 2018;23(1):131-45. doi: 10.1007/s10741-017-9661-0
5. Ponikowski P, Voors AA, Anker SD, et al. 2016 ESC Guidelines for the diagnosis and treatment of acute and chronic heart failure: The Task Force for the diagnosis and treatment of acute and chronic heart failure of the European Society of Cardiology (ESC)Developed with the special contribution of the Heart Failure Association (HFA) of the ESC. *European Heart Journal* 2016;37(27):2129-200. doi: 10.1093/eurheartj/ehw128
6. McMurray JJV, Adamopoulos S, Anker SD, et al. ESC Guidelines for the diagnosis and treatment of acute and chronic heart failure 2012 The Task Force for the Diagnosis and Treatment of Acute and Chronic Heart Failure 2012 of the European Society of Cardiology. Developed in collaboration with the Heart Failure Association (HFA) of the ESC. *Eur J Heart Fail* 2012;14(8):803-69. doi: 10.1093/eurjhf/hfs105
7. Fylan B, Marques I, Ismail H, et al. Gaps, traps, bridges and props: a mixed-methods study of resilience in the medicines management system for patients with heart failure at hospital discharge. *Bmj Open* 2019;9(2) doi: ARTN e023440
10.1136/bmjopen-2018-023440
8. (WHO) WHO. Medication safety in transition of care: World Health Organisation; 2019 [Available from: https://www.who.int/publications/i/item/WHO-UHC-SDS-2019.9 accessed 16/02 2022.
9. Simms AD, Baxter PD, Cattle BA, et al. An assessment of composite measures of hospital performance and associated mortality for patients with acute myocardial infarction. Analysis of individual hospital performance and outcome for the National Institute for Cardiovascular Outcomes Research (NICOR). *European Heart Journal: Acute Cardiovascular Care* 2013;2(1):9-18.
10. Raynor DK, Ismail H, Blenkinsopp A, et al. Experience-based co-design-Adapting the method for a researcher-initiated study in a multi-site setting. *Health Expect* 2020 doi: 10.1111/hex.13028 [published Online First: 2020/02/12]
11. Fylan B, Ismail H, Hartley S, et al. A non-randomised feasibility study of an intervention to optimise medicines at transitions of care for patients with heart failure. *Pilot and Feasibility Studies* 2021;7(1):85. doi: 10.1186/s40814-021-00819-x
12. Council MR. Developing and evaluating complex interventions  [Available from: https://mrc.ukri.org/documents/pdf/complex-interventions-guidance/.
13. National Institute for Health & Care Excellence (NICE). Nice guideline [NG106}. Chronic heart failure in adults: diagnosis and management 2018 [Available from: https://www.nice.org.uk/guidance/ng106.

For peer review only



For peer review only - http://bmjopen.bmj.com/site/about/guidelines.xhtml

14. Powell C, Breen L, Fylan B, et al. Improving the Safety and Continuity Of Medicines management at Transitions of care (ISCOMAT): protocol for a process evaluation of a cluster randomised control trial. 2020;10(11):e040493. doi: 10.1136/bmjopen-2020-040493 %J BMJ Open
15. National Institute for Health Research. Clinical Research Network [Available from: https://www.nihr.ac.uk/explore-nihr/support/clinical-research-network.htm accessed 18/01/2021 2021.
16. AUKUH Member Organisations. https://www.universityhospitals.org.uk/ [
17. Pharmoutcomes [program].
18. Puffer S, Torgerson D, Watson J. Evidence for risk of bias in cluster randomised trials: review of recent trials published in three general medical journals. *BMJ* 2003;327(7418):785-9. doi: 10.1136/bmj.327.7418.785 [published Online First: 2003/10/04]
19. Gale NK, Heath G, Cameron E, et al. Using the framework method for the analysis of qualitative data in multi-disciplinary health research. *BMC medical research methodology* 2013;13(1):117.
20. Damschroder LJ, Aron DC, Keith RE, et al. Fostering implementation of health services research findings into practice: a consolidated framework for advancing implementation science. *Implementation science* 2009;4(1):50.
21. NHS Digital. Hospital Episode Statistics (HES) Data [Available from: https://digital.nhs.uk/data-and-information/data-tools-and-services/data-services/hospital-episode-statistics accessed 18/01/2021 2021.
22. Williams A. Euroqol - a New Facility for the Measurement of Health-Related Quality-of-Life. *Health Policy* 1990;16(3):199-208.
23. Data Protection Act 2018, 2018.
24. Shah RV, McNulty S, O'connor CM, et al. Effect of admission oral diuretic dose on response to continuous versus bolus intravenous diuretics in acute heart failure: an analysis from diuretic optimization strategies in acute heart failure. *American heart journal* 2012;164(6):862-68.
25. Palazzuoli A, Pellegrini M, Ruocco G, et al. Continuous versus bolus intermittent loop diuretic infusion in acutely decompensated heart failure: a prospective randomized trial. *Critical care* 2014;18(3):R134.
26. McMurray J, Packer M, Desai A, et al. PARADIGM-HF Investigators and Committees. *Angiotensin-neprilysin inhibition versus enalapril in heart failure N Engl J Med* 2014;371(11):993-1004.
27. Campbell MK, Piaggio G, Elbourne DR, et al. Consort 2010 statement: extension to cluster randomised trials. *Bmj* 2012;345:e5661. doi: 10.1136/bmj.e5661 [published Online First: 2012/09/07]
28. Kwakkenbos L, Imran M, McCall SJ, et al. CONSORT extension for the reporting of randomised controlled trials conducted using cohorts and routinely collected data (CONSORT-ROUTINE): checklist with explanation and elaboration. 2021;373:n857. doi: 10.1136/bmj.n857 %J BMJ
29. Fanaroff AC, Cyr D, Neely ML, et al. Days Alive and Out of Hospital: Exploring a Patient-Centered, Pragmatic Outcome in a Clinical Trial of Patients With Acute Coronary Syndromes. *Circ Cardiovasc Qual Outcomes* 2018;11(12):e004755. doi: 10.1161/CIRCOUTCOMES.118.004755 [published Online First: 2018/12/19]
30. Mafham MM, Spata E, Goldacre R, et al. COVID-19 pandemic and admission rates for and management of acute coronary syndromes in England. *Lancet (London, England)* 2020;396(10248):381-89. doi: 10.1016/s0140-6736(20)31356-8 [published Online First: 2020/07/18]

For peer review only


For peer review only - http://bmjopen.bmj.com/site/about/guidelines.xhtml

31. Shoaib A, Van Spall HGC, Wu J, et al. Substantial decline in hospital admissions for heart failure accompanied by increased community mortality during COVID-19 pandemic. *European Heart Journal - Quality of Care and Clinical Outcomes* 2021 doi: 10.1093/ehjqcco/qcab040

For peer review only



For peer review only - http://bmjopen.bmj.com/site/about/guidelines.xhtml

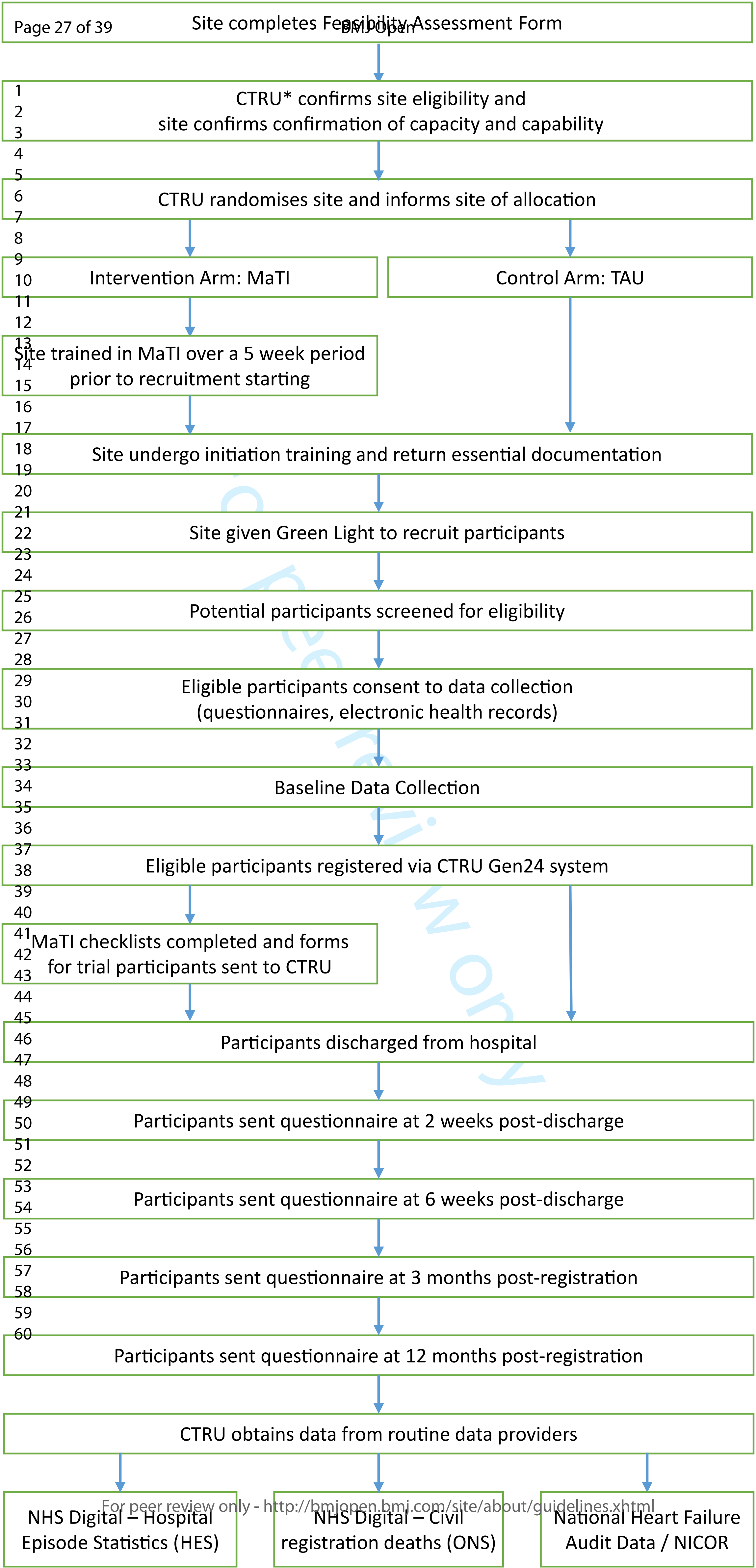
Site completes Feasibility Assessment Form
CTRU* confirms site eligibility and
site confirms confirmation of capacity and capability
CTRU randomises site and informs site of allocation
Intervention Arm: MaTI
Control Arm: TAU
Site trained in MaTI over a 5 week period
prior to recruitment starting
Site undergo initiation training and return essential documentation
Site given Green Light to recruit participants
Potential participants screened for eligibility
Eligible participants consent to data collection
(questionnaires, electronic health records)
Baseline Data Collection
Eligible participants registered via CTRU Gen24 system
MaTI checklists completed and forms
for trial participants sent to CTRU
Participants discharged from hospital
Participants sent questionnaire at 2 weeks post-discharge
Participants sent questionnaire at 6 weeks post-discharge
Participants sent questionnaire at 3 months post-registration
Participants sent questionnaire at 12 months post-registration
CTRU obtains data from routine data providers
NHS Digital – Hospital
Episode Statistics (HES)
NHS Digital – Civil
registration deaths (ONS)
National Heart Failure
Audit Data / NICOR

# MaTI Form A

## Checklist for MaTI patients

Keep this checklist in the MaTI box file in the agreed place on the ward

Patient name: ______________________________

DoB: ____________________ NHS number: ____________________

# MaTI Form A

## Checklist for MaTI patients

| *FOR STUDY USE ONLY* | | | | |
|---|---|---|---|---|
| INITIALS | | DOB | | TRIAL No. |
| SITE NAME | | | | SITE No. |

| Step | Description and tool used | When? | Date completed | Who? (role, initials) | Unable to complete (reason) |
|---|---|---|---|---|---|
| 1 | MaTI checklist in patient's notes | During admission | | | |
| 2a | Identify community pharmacist (Pharmacy form to include in notes) | During admission/ medicines reconciliation | | | |
| 2b | Contact the pharmacist to let them about the patient and discharge information | | | | |
| 3 | Patient introduced to the My Medicines Toolkit booklet (MaTI booklet) | Before the day of discharge | | | |
| 4 | Discharge heart failure medicines log completed (foldout in MaTI toolkit) | On day of discharge | | | |
| 5 | Patient informed about being referred to their community pharmacy for a follow up and a medicines use review / discussion about medicines | On day of discharge | | | |
| 6 | Transfer the patient's discharge advice letter and medicines list to the community pharmacy (MaTI community pharmacy cover letter) | At / soon after discharge | | | |
| 7 | Information has been received by the community pharmacist? (by telephone) | At / soon after discharge | Y/ N | | |

## MaTI Form B
**Community pharmacy record**

Date: ______________________

Patient name: ______________________

DoB: ______________________

## MaTI Form B
**Community pharmacy record**

| | | | | | |
|---|---|---|---|---|---|
| *FOR STUDY USE ONLY* | INITIALS | | DOB | TRIAL No. | |
| | SITE NAME | | | SITE No. | |

Date: ______________________

Community pharmacy name: ______________________

Community pharmacy address: ______________________

______________________

______________________

______________________

Phone: ______________________

Fax: ______________________

Secure NHS email: ______________________

# Community Pharmacy Cover Letter

**Ward Contact Details** Name ______________________ Telephone ______________________

**Handover of patient medicines care to community pharmacy**

Patient name: ______________________________________________

D.O.B: ______________________________________________

To the Pharmacist in charge

**A patient has now been discharged from hospital and action is required by you**

## What is this about?

We are writing to you because we have introduced improved discharge communication between our hospital and community pharmacy for patients with heart failure. It is part of a study called ISCOMAT/MaTI. For more information please see **https://www.brad.ac.uk/life-sciences/pharmacy-medical-sciences/morg/iscomat/**

## What do I need to do?

We have attached a copy of the patient's discharge advice note / letter and this includes a copy of the patient's discharge medicines list. You now need to:

- Reconcile the patient's medicines (resolve any discrepancies between the medicines in the discharge list and the patient's next repeat prescription).
- Invite the patient for a post-discharge Medicines Use Review (MUR), or a discussion about their medicines (to answer any questions they may have about their medicines, to resolve any discrepancies and to provide support as necessary).
- Complete the checklist included and keep it with your records.

If the patient wishes their MUR to take place by telephone you can apply for permission to do this using the form on the PSNC website:
**http://psnc.org.uk/services-commissioning/advanced-services/murs/conducting-murs-off-the-pharmacy-premises/**

## Free online CPD for you and your staff

If you and/or a member of your team would like to you can complete a short e-learning CPD module for staff involved with patients' medicines - to help them find out more about the risks that patients face when their care is transferred between providers. This training, which will take approximately one hour, is available free of charge to you and your team. We hope you will encourage your staff to undertake it. It has been developed by the Centre for Pharmacy Postgraduate Education (CPPE). It can be accessed here:
**www.cppe.ac.uk/canvas/iscomat**

ISCOMAT is a national study and has Health Research Authority (HRA) and Research Ethics Committee approval.

**If you require any further information about the patient, please contact staff on the ward using the contact details at the top of this letter.**

# Reporting checklist for protocol of a clinical trial.

Based on the SPIRIT guidelines.

## Instructions to authors

Complete this checklist by entering the page numbers from your manuscript where readers will find each of the items listed below.

Your article may not currently address all the items on the checklist. Please modify your text to include the missing information. If you are certain that an item does not apply, please write "n/a" and provide a short explanation.

Upload your completed checklist as an extra file when you submit to a journal.

In your methods section, say that you used the SPIRITreporting guidelines, and cite them as:

Chan A-W, Tetzlaff JM, Gøtzsche PC, Altman DG, Mann H, Berlin J, Dickersin K, Hróbjartsson A, Schulz KF, Parulekar WR, Krleža-Jerić K, Laupacis A, Moher D. SPIRIT 2013 Explanation and Elaboration: Guidance for protocols of clinical trials. BMJ. 2013;346:e7586

| | | Reporting Item | Page Number |
|---|---|---|---|
| **Administrative information** | | | |
| Title | #1 | Descriptive title identifying the study design, population, interventions, and, if applicable, trial acronym | 1 |
| Trial registration | #2a | Trial identifier and registry name. If not yet registered, | 3 |

For peer review only

| | | | |
|---|---|---|---|
| | | name of intended registry | |
| Trial registration: data set | #2b | All items from the World Health Organization Trial Registration Data Set | N/A |
| Protocol version | #3 | Date and version identifier | 6 |
| Funding | #4 | Sources and types of financial, material, and other support | 22 |
| Roles and responsibilities: contributorship | #5a | Names, affiliations, and roles of protocol contributors | 1-2; 21-2 |
| Roles and responsibilities: sponsor contact information | #5b | Name and contact information for the trial sponsor | 22 |
| Roles and responsibilities: sponsor and funder | #5c | Role of study sponsor and funders, if any, in study design; collection, management, analysis, and interpretation of data; writing of the report; and the decision to submit the report for publication, including whether they will have ultimate authority over any of these activities | 22 |
| Roles and responsibilities: committees | #5d | Composition, roles, and responsibilities of the coordinating centre, steering committee, endpoint adjudication committee, data management team, and other individuals or groups overseeing the trial, if applicable (see Item 21a for data monitoring committee) | 20 |

**Introduction**

For peer review only

| | | | |
|---|---|---|---|
| Background and rationale | #6a | Description of research question and justification for undertaking the trial, including summary of relevant studies (published and unpublished) examining benefits and harms for each intervention | 5 |
| Background and rationale: choice of comparators | #6b | Explanation for choice of comparators | 11 |
| Objectives | #7 | Specific objectives or hypotheses | 5 |
| Trial design | #8 | Description of trial design including type of trial (eg, parallel group, crossover, factorial, single group), allocation ratio, and framework (eg, superiority, equivalence, non-inferiority, exploratory) | |
| **Methods: Participants, interventions, and outcomes** | | | |
| Study setting | #9 | Description of study settings (eg, community clinic, academic hospital) and list of countries where data will be collected. Reference to where list of study sites can be obtained | 9 |
| Eligibility criteria | #10 | Inclusion and exclusion criteria for participants. If applicable, eligibility criteria for study centres and individuals who will perform the interventions (eg, | 10 |

For peer review only

| | | | |
|---|---|---|---|
| | | surgeons, psychotherapists) | |
| Interventions: description | #11a | Interventions for each group with sufficient detail to allow replication, including how and when they will be administered | 11 |
| Interventions: modifications | #11b | Criteria for discontinuing or modifying allocated interventions for a given trial participant (eg, drug dose change in response to harms, participant request, or improving / worsening disease) | N/A |
| Interventions: adherance | #11c | Strategies to improve adherence to intervention protocols, and any procedures for monitoring adherence (eg, drug tablet return; laboratory tests) | 12 |
| Interventions: concomitant care | #11d | Relevant concomitant care and interventions that are permitted or prohibited during the trial | N/A |
| Outcomes | #12 | Primary, secondary, and other outcomes, including the specific measurement variable (eg, systolic blood pressure), analysis metric (eg, change from baseline, final value, time to event), method of aggregation (eg, median, proportion), and time point for each outcome. Explanation of the clinical relevance of chosen efficacy and harm outcomes is strongly recommended | 5, 18 |
| Participant timeline | #13 | Time schedule of enrolment, interventions (including any run-ins and washouts), assessments, and visits for participants. A schematic diagram is highly recommended (see Figure) | 8 |

For peer review only

| | | | |
|---|---|---|---|
| Sample size | #14 | Estimated number of participants needed to achieve study objectives and how it was determined, including clinical and statistical assumptions supporting any sample size calculations | 6 |
| Recruitment | #15 | Strategies for achieving adequate participant enrolment to reach target sample size | |
| **Methods: Assignment of interventions (for controlled trials)** | | | |
| Allocation: sequence generation | #16a | Method of generating the allocation sequence (eg, computer-generated random numbers), and list of any factors for stratification. To reduce predictability of a random sequence, details of any planned restriction (eg, blocking) should be provided in a separate document that is unavailable to those who enrol participants or assign interventions | 9 |
| Allocation concealment mechanism | #16b | Mechanism of implementing the allocation sequence (eg, central telephone; sequentially numbered, opaque, sealed envelopes), describing any steps to conceal the sequence until interventions are assigned | 9 |
| Allocation: implementation | #16c | Who will generate the allocation sequence, who will enrol participants, and who will assign participants to interventions | 9 |

For peer review only

| | | | |
|---|---|---|---|
| Blinding (masking) | #17a | Who will be blinded after assignment to interventions (eg, trial participants, care providers, outcome assessors, data analysts), and how | 9-10 |
| Blinding (masking): emergency unblinding | #17b | If blinded, circumstances under which unblinding is permissible, and procedure for revealing a participant's allocated intervention during the trial | 9-10 |
| **Methods: Data collection, management, and analysis** | | | |
| Data collection plan | #18a | Plans for assessment and collection of outcome, baseline, and other trial data, including any related processes to promote data quality (eg, duplicate measurements, training of assessors) and a description of study instruments (eg, questionnaires, laboratory tests) along with their reliability and validity, if known. Reference to where data collection forms can be found, if not in the protocol | 15 |
| Data collection plan: retention | #18b | Plans to promote participant retention and complete follow-up, including list of any outcome data to be collected for participants who discontinue or deviate from intervention protocols | 15 |
| Data management | #19 | Plans for data entry, coding, security, and storage, including any related processes to promote data quality (eg, double data entry; range checks for data values). | 20 |

For peer review only

| | | | |
|---|---|---|---|
| | | Reference to where details of data management procedures can be found, if not in the protocol | |
| Statistics: outcomes | #20a | Statistical methods for analysing primary and secondary outcomes. Reference to where other details of the statistical analysis plan can be found, if not in the protocol | 17 |
| Statistics: additional analyses | #20b | Methods for any additional analyses (eg, subgroup and adjusted analyses) | 17 |
| Statistics: analysis population and missing data | #20c | Definition of analysis population relating to protocol non-adherence (eg, as randomised analysis), and any statistical methods to handle missing data (eg, multiple imputation) | 17, 20 |
| **Methods: Monitoring** | | | |
| Data monitoring: formal committee | #21a | Composition of data monitoring committee (DMC); summary of its role and reporting structure; statement of whether it is independent from the sponsor and competing interests; and reference to where further details about its charter can be found, if not in the protocol. Alternatively, an explanation of why a DMC is not needed | N/A |
| Data monitoring: interim analysis | #21b | Description of any interim analyses and stopping guidelines, including who will have access to these interim results and make the final decision to terminate the trial | 18 |
| Harms | #22 | Plans for collecting, assessing, reporting, and managing solicited and spontaneously reported adverse events and other unintended effects of trial interventions or trial | 14 |

For peer review only

| | | | |
|---|---|---|---|
| | | conduct | |
| Auditing | #23 | Frequency and procedures for auditing trial conduct, if any, and whether the process will be independent from investigators and the sponsor | 20 |
| **Ethics and dissemination** | | | |
| Research ethics approval | #24 | Plans for seeking research ethics committee / institutional review board (REC / IRB) approval | 4, 20 |
| Protocol amendments | #25 | Plans for communicating important protocol modifications (eg, changes to eligibility criteria, outcomes, analyses) to relevant parties (eg, investigators, REC / IRBs, trial participants, trial registries, journals, regulators) | 4 |
| Consent or assent | #26a | Who will obtain informed consent or assent from potential trial participants or authorised surrogates, and how (see Item 32) | 9 |
| Consent or assent: ancillary studies | #26b | Additional consent provisions for collection and use of participant data and biological specimens in ancillary studies, if applicable | N/A |
| Confidentiality | #27 | How personal information about potential and enrolled participants will be collected, shared, and maintained in order to protect confidentiality before, during, and after the trial | 14 |
| Declaration of | #28 | Financial and other competing interests for principal | 22 |

For peer review only

| | | | |
|---|---|---|---|
| interests | | investigators for the overall trial and each study site | |
| Data access | #29 | Statement of who will have access to the final trial dataset, and disclosure of contractual agreements that limit such access for investigators | 22 |
| Ancillary and post trial care | #30 | Provisions, if any, for ancillary and post-trial care, and for compensation to those who suffer harm from trial participation | N/A |
| Dissemination policy: trial results | #31a | Plans for investigators and sponsor to communicate trial results to participants, healthcare professionals, the public, and other relevant groups (eg, via publication, reporting in results databases, or other data sharing arrangements), including any publication restrictions | 4 |
| Dissemination policy: authorship | #31b | Authorship eligibility guidelines and any intended use of professional writers | 4 |
| Dissemination policy: reproducible research | #31c | Plans, if any, for granting public access to the full protocol, participant-level dataset, and statistical code | 22 |
| **Appendices** | | | |
| Informed consent materials | #32 | Model consent form and other related documentation given to participants and authorised surrogates | |
| Biological specimens | #33 | Plans for collection, laboratory evaluation, and storage of biological specimens for genetic or molecular analysis in the current trial and for future use in ancillary studies, if applicable | N/A |

For peer review only

None The SPIRIT Explanation and Elaboration paper is distributed under the terms of the Creative Commons Attribution License CC-BY-NC. This checklist can be completed online using https://www.goodreports.org/, a tool made by the EQUATOR Network in collaboration with Penelope.ai

For peer review only

BMJ Open

# Using Routine Healthcare Data to evaluate the impact of the Medicines at Transitions Intervention (MaTI) on clinical outcomes of patients hospitalised with heart failure: protocol for the Improving the Safety and Continuity Of Medicines management at Transitions of care (ISCOMAT) cluster randomised controlled trial with embedded process evaluation, health economics evaluation and internal pilot

| | |
|---|---|
| Journal: | *BMJ Open* |
| Manuscript ID | bmjopen-2021-054274.R2 |
| Article Type: | Protocol |
| Date Submitted by the Author: | 15-Mar-2022 |
| Complete List of Authors: | Moreau, Lauren; University of Leeds Clinical Trials Research Unit, CTRU<br>Holloway, Ivana; University of Leeds Clinical Trials Research Unit<br>Fylan, Beth; University of Bradford, School of Pharmacy and Medical Sciences; NIHR Yorkshire and Humber PSTRC, Bradford Insitiue for Health Research<br>Hartley, Suzanne; University of Leeds Clinical Trials Research Unit, Clinical Trials Research Unit<br>Cundill, Bonnie; University of Leeds Clinical Trials Research Unit<br>Fergusson, Alison; University of Leeds Clinical Trials Research Unit<br>Alderson, Sarah; University of Leeds, Academic Unit of Primary Care<br>Alldred, David; University of Leeds, School of Healthcare; NIHR Yorkshire and Humber Patient Safety Translational Research Centre, Bradford Institute for Health Research<br>Bojke, Chris; University of Leeds, Academic Unit of Health Economics<br>Breen, Liz; University of Bradford, School of Pharmacy and Medical Sciences; University of Bradford<br>Ismail, Hanif; University of Bradford, School of Pharmacy and Medical Sciences<br>Gardner, Peter; University of Bradford, School of Pharmacy and Medical Sciences; University of Bradford, Wolfson Centre for Applied Health Research<br>Mason, Ellen; University of Leeds Clinical Trials Research Unit<br>Powell, Catherine; University of Bradford Faculty of Health Studies, Centre for Applied Dementia Studies<br>Silcock, Jonathan; University of Bradford School of Pharmacy and Medical Sciences<br>Taylor, Andrew; ISCOMAT Patient Led Steering Group<br>Farrin, Amanda; University of Leeds Clinical Trials Research Unit<br>Gale, Chris; University of Leeds Leeds Institute of Cardiovascular and Metabolic Medicine; Leeds Teaching Hospitals NHS Trust Department of Cardiology |
| <b>Primary Subject Heading</b>: | Cardiovascular medicine |

For peer review only - http://bmjopen.bmj.com/site/about/guidelines.xhtml

| | |
|---|---|
| Secondary Subject Heading: | Research methods |
| Keywords: | Heart failure < CARDIOLOGY, Cardiology < INTERNAL MEDICINE, Clinical trials < THERAPEUTICS |
| | |

SCHOLARONE™
Manuscripts

**Title**

Using Routine Healthcare Data to evaluate the impact of the Medicines at Transitions Intervention (MaTI) on clinical outcomes of patients hospitalised with heart failure: protocol for the Improving the Safety and Continuity Of Medicines management at Transitions of care (ISCOMAT) cluster randomised controlled trial with embedded process evaluation, health economics evaluation and internal pilot

**Names of protocol contributors**

Lauren Moreau[1], Ivana Holloway[1], Beth Fylan[2, 3], Suzanne Hartley[1], Bonnie Cundill[1], Alison Fergusson[1], Sarah Alderson[4], David Alldred[3, 4], Chris Bojke[5], Liz Breen[2, 3], Hanif Ismail[2], Peter Gardner[2], Ellen Mason[1], Catherine Powell[2], Jon Silcock[2, 3], Andrew Taylor[6], Amanda J. Farrin[1], Chris P. Gale[7]. On behalf of the ISCOMAT Programme Management Team

[1]Leeds Institute for Clinical Trials Research, Clinical Trials Research Unit, University of Leeds, Leeds, UK LS2 9JT

[2]School of Pharmacy and Medical Sciences, Faculty of Life Sciences, University of Bradford, Bradford, UK; Wolfson Centre for Applied Health Research, Bradford, UK

[3]NIHR Yorkshire and Humber Patient Safety Translational Research Centre, Bradford, UK

[4]School of Healthcare, University of Leeds, Leeds, UK

[5]Academic Unit for Health Economics, University of Leeds, Leeds UK LS2 9JT

[6] ISCOMAT Patient Led Steering Group, Manchester UK

[7]Leeds Institute of Cardiovascular and Metabolic Medicine, University of Leeds, Leeds, UK; Leeds Institute for Data Analytics, University of Leeds, Leeds, UK; Department of Cardiology, Leeds Teaching Hospitals NHS Trust, Leeds, UK

Lauren Moreau (corresponding author), Leeds Institute for Clinical Trials Research, Clinical Trials Research Unit, University of Leeds, Leeds, UK LS2 9JT; 0113 343 7588; l.a.moreau@leeds.ac.uk

Ivana Holloway, Leeds Institute for Clinical Trials Research, Clinical Trials Research Unit, University of Leeds, Leeds, UK LS2 9JT; I.Holloway@leeds.ac.uk

Beth Fylan, School of Pharmacy and Medical Sciences, Faculty of Life Sciences, University of Bradford, Bradford, UK; Wolfson Centre for Applied Health Research, Bradford, UK; B.Fylan@bradford.ac.uk

Suzanne Hartley, Leeds Institute for Clinical Trials Research, Clinical Trials Research Unit, University of Leeds, Leeds, UK LS2 9JT; S.Hartley@leeds.ac.uk

Bonnie Cundill, Leeds Institute for Clinical Trials Research, Clinical Trials Research Unit,

For peer review only


For peer review only - http://bmjopen.bmj.com/site/about/guidelines.xhtml

University of Leeds, Leeds, UK LS2 9JT; B.E.Cundill@leeds.ac.uk

Alison Fergusson, Leeds Institute for Clinical Trials Research, Clinical Trials Research Unit, University of Leeds, Leeds, UK LS2 9JT; A.Fergusson@leeds.ac.uk

Sarah Alderson, Leeds Institute for Health Sciences, University of Leeds, Leeds, UK LS2 9JT; S.L.Alderson@leeds.ac.uk

David Alldred, Wolfson Centre for Applied Health Research, Bradford, UK; Bradford Institute for Health Research, NIHR Yorkshire and Humber Patient Safety Translational Research Centre, Bradford, UK; School of Healthcare, University of Leeds, Leeds, UK; D.P.Alldred@leeds.ac.uk

Chris Bojke, Academic Unit for Health Economics, University of Leeds, Leeds UK LS2 9JT; C.Bojke@leeds.ac.uk

Liz Breen School of Pharmacy and Medical Sciences, Faculty of Life Sciences, University of Bradford, Bradford, UK; Wolfson Centre for Applied Health Research, Bradford, UK; Bradford Institute for Health Research, NIHR Yorkshire and Humber Patient Safety Translational Research Centre, Bradford, UK; L.Breen@bradford.ac.uk

Hanif Ismail School of Pharmacy and Medical Sciences, Faculty of Life Sciences, University of Bradford, Bradford, UK; Wolfson Centre for Applied Health Research, Bradford, UK; H.Ismail11@bradford.ac.uk

Peter Gardner School of Pharmacy and Medical Sciences, Faculty of Life Sciences, University of Bradford, Bradford, UK; Wolfson Centre for Applied Health Research, Bradford, UK; P.H.Gardner@bradford.ac.uk

Ellen Mason, Leeds Institute for Clinical Trials Research, Clinical Trials Research Unit, University of Leeds, Leeds, UK LS2 9JT; E.Mason1@leeds.ac.uk

Catherine Powell School of Pharmacy and Medical Sciences, Faculty of Life Sciences, University of Bradford, Bradford, UK; Wolfson Centre for Applied Health Research, Bradford, UK; C.Powell2@bradford.ac.uk

Jon Silcock School of Pharmacy and Medical Sciences, Faculty of Life Sciences, University of Bradford, Bradford, UK; Wolfson Centre for Applied Health Research, Bradford, UK; J.Silcock@bradford.ac.uk

Andrew Taylor, ISCOMAT Patient Led Steering Group, Manchester UK; ataylor102@outlook.com

Amanda J. Farrin Leeds Institute for Clinical Trials Research, Clinical Trials Research Unit, University of Leeds, Leeds, UK LS2 9JT; A.J.Farrin@leeds.ac.uk

Chris P. Gale Leeds Institute of Cardiovascular and Metabolic Medicine, University of Leeds, Leeds, UK; Leeds Institute for Data Analytics, University of Leeds, Leeds, UK; Department of Cardiology, Leeds Teaching Hospitals NHS Trust, Leeds, UK; c.p.gale@leeds.ac.uk

**Word count: 4016**


For peer review only - http://bmjopen.bmj.com/site/about/guidelines.xhtml

## Abstract

### Introduction

Heart failure affects 26 million people globally, approximately 900 thousand people in the UK, and is increasing in incidence. Appropriate management of medicines for heart failure at the time of hospital discharge reduces readmissions, improves quality of life and increases survival. The ISCOMAT trial tests the effectiveness of the Medicines at Transition Intervention (MaTI) which aims to enhance self-care and increase community pharmacy involvement in the medicines management of heart failure patients.

### Methods and analysis

ISCOMAT is a parallel-group cluster randomised controlled trial, randomising 42 National Health Service trusts with cardiology wards in England on a 1:1 basis to implement the MaTI or treatment as usual. Around 2100 patients over the age of 18, admitted to hospital with heart failure with at least moderate left ventricular systolic dysfunction within the last 5 years, and planned discharge to the geographical area of the cluster will be recruited. The MaTI consists of training for staff, a toolkit for participants, transfer of discharge information to community pharmacies, and a medicines reconciliation/review. Treatment as usual is determined by local policy and practices. The primary outcome is a composite of all-cause mortality and heart failure-related hospitalisation at 12 months post-registration obtained from national electronic health records. The key secondary outcome is continued prescription of guideline-indicated therapies at 12-months measured via patient-reported data and Hospital Episode Statistics. The trial contains a parallel mixed-methods process evaluation and an embedded health economics study.

### Ethics and dissemination

The study obtained approval from the Yorkshire and the Humber – Bradford Leeds Research Ethics Committee; REC reference 18/YH/0017. Findings will be disseminated via academic and policy conferences, peer-reviewed publications and social media. Amendments to the protocol are disseminated to all relevant parties as required.



### Registration

ISRCTN66212970

### Key words

Heart failure; cardiology; clinical trials; cluster trial; medicines




For peer review only - http://bmjopen.bmj.com/site/about/guidelines.xhtml

## Article summary

### Strengths and limitations of this study

- ISCOMAT is a trial which evaluates a process to optimise medicines use for patients discharged from hospital with heart failure to their usual place of residence
- The trial intervention was developed through experience-based co-design with patients and health care professionals
- The trial will use nationwide patient-level health records for the primary endpoint
- Due to intervention implementation requirements, patients were recruited from designated cardiology wards only
- Clinical events cannot be centrally adjudicated as they arise from electronic health records. As such this is a pragmatic trial with results generalizable to the 'real world' environment.

For peer review only


For peer review only - http://bmjopen.bmj.com/site/about/guidelines.xhtml

**Background**

Heart failure affects 26 million people globally,[1] approximately 900 thousand people in the UK, and is increasing in incidence.[2] People with heart failure frequently use multiple healthcare services,[3] so the economic burden of heart failure is substantial and is further compounded by high rates of hospitalisation and subsequent readmission.[4]

Heart failure may be managed through a combination of pharmacological treatments at titrated doses including angiotensin-converting enzyme (ACE) inhibitors, beta-adrenoceptor antagonists (beta-blockers), mineralocorticoid/aldosterone receptor antagonists (MRA), angiotensin receptor neprilysin inhibitors, sodium-glucose transport protein 2 (SGLT2) inhibitors and diuretics as well as implanted devices.[5] Medicines optimisation reduces hospitalisation, improves quality of life and increases survival rates.[6] However, this requires the coordinated input of several healthcare professionals, following hospitalisation, which may be subject to variation.[7] Medicines management at care transitions is problematic in healthcare systems internationally.[8] Estimated readmissions rate within three months of discharge for patients with heart failure are as high as 50%[9], indicating a need to achieve improvements in the continuity of care at the time of transfer to the community.

The Medicines at Transitions Intervention (MaTI) was designed to improve the use of prescribed medicines when patients with heart failure are discharged from hospital. It was developed through qualitative research and co-design with patients and healthcare staff.[7 10] Intervention feasibility was tested in three healthcare areas with 31 participants in northern England in 2017.[11] Following the feasibility study, the intervention was refined before progressing to the main trial. [11] Following Medical Research Council guidance for the design and evaluation of complex interventions,[12] we report the protocol of the ISCOMAT trial, a cluster randomised controlled trial to evaluate the clinical and cost-effectiveness of the MaTI among heart failure patients.

**Methods and design**

## Aim and Objectives

The overall aim of ISCOMAT (ISRCTN Registration 66212970) is to evaluate the effectiveness of the MaTI on the delivery of guideline recommended care and clinical outcomes for patients who are discharged from hospital with heart failure, when compared with treatment as usual.

The primary objective is to establish whether the MaTI reduces all-cause mortality and heart failure rehospitalisation measured over 12 months from registration.

The key secondary objective is to determine whether the intervention increases the proportion of participants being prescribed the composite of guideline-indicated therapies at 12 months post-


For peer review only - http://bmjopen.bmj.com/site/about/guidelines.xhtml

registration (i.e. ACE inhibitor, angiotensin II receptor blocker (ARB) or salcubitril/valsartan, beta blocker and/or ivabradine and MRA).[13]

Other secondary objectives, measured at 12-months post-registration unless stated otherwise, are to establish if the intervention:

- Increases time to all-cause mortality
- Increases time to heart failure-related rehospitalisation
- Increases time on guideline recommended (and indicated as above) cardiovascular medicines.
- Improves patient understanding of their medicines and satisfaction with medicines-related care at 2- and 6-week post-discharge and 12-months post-registration
- Increases number of days alive and out of hospital
- Reduces hospitalisations (all cause, cardiovascular related and heart failure)
- Reduces mortality due to specific causes.
- Is cost-effective

The internal pilot objective is to assess whether the trial recruitment meets the pre-defined progression criteria thresholds.

The objectives of the mixed-methods process evaluation are to determine intervention fidelity; the relationship between implementation/fidelity and outcomes; and the barriers or facilitators to the successful implementation and roll out of the intervention.[14]

**Trial Design**

This publication describes REC approved Study Protocol v4.0 and dated 08 August 2018.

ISCOMAT is a parallel-group cluster randomised controlled trial that aims to recruit 2100 participants hospitalised with heart failure from 42 National Health Service (NHS) trusts in England (approximately 50 participants per site) over a 12-month recruitment period. Clusters are defined as a distinct geographical area and consist of the NHS trust with a cardiology ward(s) and/or a Coronary Care Unit (CCU; defined as a ward with nursing staff with specific cardiac nursing expertise) together with associated community pharmacies within the Clinical Commissioning Group boundaries in that geographical area (Figure 1).

For peer review only


For peer review only - http://bmjopen.bmj.com/site/about/guidelines.xhtml

ISCOMAT was purposively designed to utilise the routine data sources available through providers such as NHS Digital, the Office for National Statistics and the National Heart Failure Audit. The use of routine health records data has enabled the trial to reduce the burden of data collection on research teams and patients and ensure a high level of accuracy with minimal data loss for the primary endpoint.

***[Insert** Figure 1: Trial process diagram here]*

For peer review only


For peer review only - http://bmjopen.bmj.com/site/about/guidelines.xhtml

### Cluster eligibility

Acute NHS Trusts with accompanying cardiology wards that met all of the inclusion criteria were eligible to take part in the study as described in Table 1.

*Table 1: Site Eligibility Criteria*

| Eligibility Criteria | Details |
|---|---|
| Inclusion | Commitment to achieve the recruitment target |
| | Participation in the National Heart Failure Audit[1] |
| | Hospital Episode Statistics available via NHS Digital |
| | Deemed suitable to implement and deliver the intervention (as determined by the ISCOMAT team).[2] |
| Exclusion | Already providing medicines management deemed to be sufficiently similar to the MaTI |

[1] Participation in UK National Heart Failure Audit was required to facilitate acquiring the dataset from NICOR
[2] Sites were deemed suitable where there was at least one designated cardiology ward and/or CCU and had an existing mechanism to communicate with community pharmacies for all heart failure patients (or were willing to introduce one).

### Randomisation and blinding

NHS Trusts were required to obtain all necessary local management approvals prior to randomisation and this was used to evidence consent. Randomisation was undertaken independently by the Leeds Clinical Trials Research Unit (CTRU). Clusters were allocated on a 1:1 basis either to implement the MaTI or treatment as usual (TAU) using minimisation with a random element, minimised by:

- geographical region, as defined by National Institute for Health Research Clinical Research Networks;[15]
- type of hospital (university hospital or non-university hospital as defined by membership of the University Hospital Association)[16]; and
- method of clinical handover/information transfer to community pharmacy (electronic system, e.g. PharmOutcomes;[17] or manual system, e.g. standard email, fax, letter or phone).

### Participant eligibility and registration

Patient recruitment commenced approximately four weeks following cluster randomisation. Potential participants were screened following admission to hospital and recruited prior to discharge. Patient screening, consent, recruitment and baseline data collection was completed by research staff, who had no role in the delivery of care or treatment and, wherever possible, were


For peer review only - http://bmjopen.bmj.com/site/about/guidelines.xhtml

blinded to the cluster allocation to mitigate selection bias.[18] Site staff were asked to self-report instances of unblinding to CTRU. This was monitored at monthly Trial Management Group Meetings (TMG) and annual Trial Steering Committee (TSC) meetings. Consented participants meeting the eligibility criteria described in Table 2 Table 2 are included in the trial.

*Table 2: Participant Eligibility Criteria*

| **Eligibility Criteria** | **Details** |
|---|---|
| Inclusion | Admitted or transferred to a ward participating in the ISCOMAT trial |
| | Heart failure with evidence of at least moderate left ventricular systolic dysfunction* confirmed within the last 5 years.<br>**Defined as: a left ventricular ejection fraction of less than or equal to 44%, quantified via echocardiogram; or the equivalent of this, if quantified by a different imaging modality.* |
| | Aged 18 years or over at hospital admission. |
| | Planned discharged from recruiting hospital to their home (defined by usual place of residence) or a care home. |
| | Planned discharge within geographical area of that cluster |
| | Capacity to provide informed consent |
| | Provide informed consent |
| Exclusion | Patients in a terminal phase of illness / end of life care pathway who were not expected to survive beyond 6 weeks post-discharge. |
| | Patients already participating in ISCOMAT (e.g. re-admissions) |

For peer review only

Documented reasons for ineligibility or declining participation were closely monitored by the trial team as part of a regular review of recruitment progress, to ensure generalisability of study results in accordance with CONSORT reporting guidelines, and to highlight issues in identifying or recruiting patients during the internal pilot.

Following confirmation of written informed consent and eligibility, participants were registered into the trial by an authorised member of staff at the trial research site, via a central automated 24-hour online registration system hosted at CTRU.


For peer review only - http://bmjopen.bmj.com/site/about/guidelines.xhtml

## Trial status

The first trial site was randomised on 16th April 2018, opened to recruitment on 1st June 2018 and the first participant was recruited on 8th June 2018. A total of 44 clusters were randomised, of which 43 opened to recruitment and registered at least one participant. Overall 1641 participants consented to take part. Recruitment was due to end on 31st December 2019 but was extended to 31st December 2020. Following discussion with the sponsor and in line with government guidance, recruitment at all sites paused on 18th March 2020 due to the COVID-19 pandemic and following consultation with the TSC/Programme Steering Committee (PSC)/funder the decision was taken to close ISCOMAT to recruitment on 28th July 2020.

## Governance

The trial is funded by a National Institute for Health Research (NIHR) Programme Grant for Applied Research (Reference: RP-PG-0514-20009. The views expressed in this paper are those of the authors and not necessarily those of the NIHR or the Department of Health and Social Care. The trial is sponsored by Bradford Teaching Hospitals NHS Foundation Trust.

## Intervention

The MaTI was designed to be implemented by differently specialised professional clinical teams, including nurses and pharmacists. The intervention was initiated by the discharge nurse (or equivalent) and continued post-discharge through involvement of the community pharmacist. MaTI comprises of:

1) Patient-held personalisable medicines toolkit containing: information about heart failure, medicines and their effects; details of the patients' healthcare team; a checklist of medicines-related activity that should occur in hospital before discharge, at discharge and once the patient has returned home; a traffic-light guide to symptoms plus actions to take if symptoms worsen. This will be delivered to patients face-to-face by a nurse of pharmacist from the cardiology ward as part of routine care at intervention sites. See supplementary files MATI form A and B for example checklists completed at site.
2) Enhanced communication between hospitals and the patients' community pharmacists through transfer of the patient's discharge letter and medicines list to community pharmacy, with a request for the community pharmacist to reconcile the patient's medicines and invite the patient for a medicines use review (MUR) or a discussion about their medicines. See supplementary file of Community pharmacy cover letter.

The intervention was delivered to all eligible patients as part of routine care. Clinical staff were not aware which patients have consented to data collection and evaluation.



For peer review only - http://bmjopen.bmj.com/site/about/guidelines.xhtml

### Intervention Training

Clusters randomised to the intervention were asked to identify key members of the clinical team involved in medicines management (e.g. ward nurses, hospital pharmacists, primary care heart failure nurses) to complete intervention training online and in-person. The intervention team aimed to train around 5-7 staff members in each hospital site to enable the site coordinator and nominated staff to brief others about the MaTI. The online training was developed with the Centre for Pharmacy Postgraduate Education (www.cppe.ac.uk) to enable staff to gain an understanding of the MaTI. Face-to-face training explained the background to MaTI (importance, purpose, design, outcomes) and went through the supporting materials/ guides designed to aid facilitation of delivery. Approximately one month was allowed in each cluster for training and embedding the MaTI into routine practice prior to study recruitment commencing. Each cluster identified one member of staff to adopt the 'site coordinator' role, responsible for ensuring the intervention was implemented according to the guidance and training provided. The number and role of hospital staff trained in each cluster was dependent on the structure of cardiology/pharmacy services and available staff.  After 3-4 weeks, further follow up visits were arranged to ensure adherence to the protocol.

Staff delivery of the intervention on an individual level was communicated to intervention team regularly to support ongoing implementation of the intervention. Additional support and training were available when required.

### Measuring adherence to the intervention

The TMG monitored adherence to intervention protocols every month by reviewing delivery of key intervention components across all intervention sites. Performance feedback was provided to site teams via monthly emails. Data on adherence was also reviewed at annual TSC meetings.

### Treatment as usual

Treatment as usual was determined by local Trust policies and practices. Variations in treatment as usual were expected across NHS cardiology services in England and documented in site feasibility assessments, during recruitment, and at the end of follow-up.

### Process Evaluation

A parallel embedded process evaluation aims to inform the trial findings and aid potential future wider scale implementation. The process evaluation involved six purposively selected intervention sites using a mixed-methods design. Fidelity and barriers/enablers of implementation of MaTI were explored using observation, interviews of up to 20 patients and 40 healthcare professionals, staff


For peer review only - http://bmjopen.bmj.com/site/about/guidelines.xhtml
For peer review only

surveys and trial-specific data collection on MaTI adherence.[14] A parallel mixed analysis is planned. Qualitative data is thematically analysed using Framework analysis and survey data is analysed using descriptive statistics. Data is synthesised, triangulated and mapped to the Consolidated Framework for Implementation Research (CFIR)[19 20] where appropriate. A full description of methods are published in Powell et al.[14]

**Outcome measures**

The primary outcome is the binary composite endpoint of all-cause mortality and heart failure-related hospitalisation within 12 months post-registration (see Box 1 for definition of hospitalisation). Mortality data is obtained from the Civil Registration Death data from the Office for National Statistics, and hospitalisation data from Hospital Episode Statistics (HES)[21] from NHS Digital. Routine data for the analysis of primary and secondary outcomes is supported by site reported and patient reported data where appropriate.

Box 1: Definition of hospitalisation

| |
|---|
| **Definition of hospitalisation:** Hospitalisation is defined as an acute inpatient admission (regardless of length of stay). A hospitalisation will be considered heart-failure related if the primary reason / diagnosis for admission included ICD-10 codes or procedural classifications related to heart failure |

The key secondary outcome is participants still being prescribed at least one of the guideline recommended therapies at 12 months in each of the following three groups:

- ACE inhibitor (ACEI); angiotensin II receptor blocker (ARB); salcubitril/valsartan
- Beta blocker; ivabradine
- Mineralocorticoid receptor antagonist (MRA)

For patients with contraindications to any of the three groups, the endpoint will be derived with respect to groups that are indicated. Data is collected via patient-reported questionnaires collected at baseline, 3-month and 6-month post-registration. We also plan to obtain data from the NHS Business Service Authority on medicines dispensed in primary care, via NHS Digital.

The following secondary outcomes are collected over the 12-month post-registration via routine data sources:

- Individual components of the primary endpoint regarded as time to event endpoints. Time will be measured from registration to the first day of heart failure rehospitalisation or death
- Length of time on guideline recommended cardiovascular medications
- Days alive and out of hospital: defined as the number of days in the year beginning the day after registration that the patient spends alive and not in hospital


For peer review only - http://bmjopen.bmj.com/site/about/guidelines.xhtml
For peer review only

- Time to cardiovascular-related hospitalisation and all cause hospitalisation
- Mortality due to specific causes
- Healthcare resource use

The following secondary outcomes are measured at baseline, and 3-month and 12-month post-registration unless stated otherwise:

- Patient satisfaction with their medicines care and patient understanding of their medicines; measured via a patient experience survey at 2 and 6-weeks post-discharge, and 12-months post-registration with a 10-point Likert scale. For intervention participants data on the use of the MaTI over a 12-month post-registration period is also captured.
- EuroQol5-Dimension Health Questionnaire (3 levels) (EQ-5D-3L)[22]; measures health utility (quality of life) comprising five dimensions: mobility, self-care, usual activities, pain/discomfort and anxiety/depression. Dimension responses are combined and converted into a summary index (0 for dead, 1 for perfect health and negative values for states worse than death).
- Healthcare resource use: additional to routine datasets, we capture patient reported healthcare resource use using standard self-reported resource use questionnaires, to include hospital attendances, GP, nurse, specialist heart failure nurse and pharmacy contact.

## Data collection

The outcome assessment schedule is outlined in Table 3**Error! Reference source not found.**. All personal information is processed in accordance with the Data Protection Act 2018[23] (and any successor legislation). Appropriate storage, restricted access and disposal arrangements for personal and clinical details are in place. Archiving is for a minimum of 10 years.

*Routine Data*

ISCOMAT will obtain data from routine providers NHS Digital (including HES and ONS data) and NICOR and the UK National Heart Failure Audit for the primary and secondary outcomes as outlined above. The trial was designed to use data from national routine sources to maximise efficiency in answering the research questions, ensure consistency across multiple sites, and minimise burden on patients and research staff at hospitals.

*Clinical Data*

Recruiting teams collected information on study eligibility and details of the participants' admission and discharge, including heart failure history, clinical details, primary aetiology for heart failure,


For peer review only - http://bmjopen.bmj.com/site/about/guidelines.xhtml
For peer review only

detailed medicines information, and related unexpected serious adverse events (RUSAEs). All-cause mortality and re-admission data were collected throughout the study up until 12 months post registration from hospital sites and electronic health records (NHS Digital). Consent was sought to obtain data from electronic health records – including secondary care records and NHS Digital.

*Participant reported data*

Patient self-reported baseline measures were collected in hospital inpatient setting. Patient reported outcomes at 2- and 6-week post discharge, and 3- and 12-months post registration were collected via post. When questionnaires were not returned, the CTRU sent postal reminders.

*Intervention fidelity data and quantitative and qualitative data from observations, participant and staff interviews and surveys*

Clinical teams, randomised to the intervention, returned a checklist of seven steps for each trial participant, confirming completion of intervention activity and local pharmacy information. The CTRU sought data from the participants' community pharmacy following discharge. Details about the heart failure service models and the clinical role of those staff responsible for the delivery of the MaTI were documented.

*Table 3: Schedule of enrolment, interventions and assessments for participants*

| Assessment | Type | Method of Completion | Timepoint | | | | | |
|---|---|---|---|---|---|---|---|---|
| | | | Screening | Baseline | 2 weeks post-discharge | 6 weeks post-discharge | 3 months post-registration | 12 months post-registration |
| PARTICIPANT DATA | | | | | | | | |
| Screening (demographics / assessment of eligibility) | CRF[1] | CRN[2] / local research staff | X | | | | | |
| Consent | Consent Form | Self-completion | | X | | | | |
| Eligibility | CRF | CRN / local research staff | | X | | | | |
| Demographics | CRF | CRN / local research staff | | X | | | | |
| Contact Details | CRF | CRN / local research staff | | X | | | | |
| Admission/discharge details | CRF | CRN / local research staff | | X | | | | |


For peer review only - http://bmjopen.bmj.com/site/about/guidelines.xhtml

| | | | | | | | | |
|---|---|---|---|---|---|---|---|---|
| Health Related Quality of Life (EuroQol EQ-5D-3L) | Questionnaire booklet (post) | Self-completion | | X | | | X | X |
| Healthcare Resource Use (in last 3 months) | Questionnaire booklet (post) | Self-completion | | X | | | X | X |
| Current heart failure medications | Questionnaire booklet (post) | Self-completion | | | | | X | X |
| Patient Experience Survey | Questionnaire booklet (post) | Self-completion | | | X | X | | X |
| Primary outcome (all-cause mortality and HF rehospitalisation) | Routine data / CRF | Data Access Request: Routine data providers > CTRU[3] | | DATA EXTRACTS TO BE AGREED WITH DATA PROVIDERS TO ALLOW 12 MONTH DATA COLLECTION | | | | |
| Secondary outcomes | Routine data / CRF | Data Access Request: Routine data providers > CTRU | | DATA EXTRACTS TO BE AGREED WITH DATA PROVIDERS TO ALLOW 12 MONTH DATA COLLECTION | | | | |
| Patient status | CRF | CTRU | | ONGOING | | | | |

| SITE DATA | | | | | |
|---|---|---|---|---|---|
| Assessment | Type | Method of Completion | Baseline | 6 months post-randomisation | 12 months post-implementation of intervention |
| Site questionnaire | Questionnaire Booklet | CTRU | X | X | X |

| INTERVENTION DATA | | | | |
|---|---|---|---|---|
| Assessment | Type | Method of Completion | 3 months post-registration | 6 months post-implementation of intervention |
| Adherence / fidelity data (collection of patient checklists to confirm what happened at discharge, MUR review confirmation from the pharmacy) | CRF | CTRU | ONGOING | |
| Interviews - Patients | Process Evaluation | Research Fellow | X | |
| Interviews - Staff | Process Evaluation | Research Fellow | | x |
| Survey - Staff | Process Evaluation | Research Fellow | | x |
| Observations - ward | Process Evaluation | Research Fellow | | x |

[1]Case report form (CRF)
[2]Clinical Research Network (CRN)
[3]Clinical Trials Research Unit (CTRU)

**Statistical methods**


For peer review only - http://bmjopen.bmj.com/site/about/guidelines.xhtml

## Sample size

Sample size calculations were based on previous research[24 25] indicating that the combined primary event rate is likely to be at least 20% in control sites. With 42 clusters, an estimated intracluster correlation coefficient (ICC) of 0.01, a coefficient of variation in cluster size of 0.23, and 15% loss to follow-up (based on studies in this patient group),[26] 50 patients with heart failure in each cluster (2100 in total) are required to provide at least 80% power at 5% significance level to detect a minimal clinically important difference (MCID) of 6%.[26] The MaTI is delivered to each heart failure participant by dispersed teams across the care transition (secondary-care, primary-care, community pharmacy), therefore, the anticipated ICC is likely to be relatively low.

This sample size also provides power in excess of 90% to detect a MCID of 12% in the key secondary outcome even with the most conservative assumption for the control rate.

## Internal pilot

Descriptive statistics were used to evaluate progression criteria assessing site-level recruitment rates. This analysis informed study continuation beyond the internal pilot phase. The progression criterion was assessed at 6-months after recruitment commenced, based on the following traffic light system of green (go), amber (review) and red (stop):

Green: ≥4 patients/month/NHS trust;

Amber: ≥2 but <4 patients/month/NHS trust;

Red: <2 patients/month/NHS trust.

## Data analysis

A full statistical analysis plan (SAP) will be finalised prior to any analyses. The CTRU will conduct the statistical analysis and data summaries on an intention-to-treat (ITT) population, which is defined as all clusters randomised and all participants registered regardless of non-compliance with the protocol or withdrawal from the study. The study is conducted and will be reported according to the CONSORT extensions for cluster trials[27] and CONSORT-ROUTINE[28] for trials conducted using cohorts and routinely collected data. Baseline data will be summarised by treatment group and at the participant level and/or the cluster level using descriptive statistics as appropriate.


For peer review only - http://bmjopen.bmj.com/site/about/guidelines.xhtml

Additionally, the SAP will describe the derivation of outcomes and detail the analyses for each outcome measure. The analyses models will reflect clustering and include fixed effects for the minimisation factors with clusters as random effects, see Table 4.

A complier average causal effect (CACE) analysis approach to estimate the treatment effect for the primary outcome amongst compliant clusters will be considered depending on levels of adherence to the intervention as intended. Definitions of adherence and pre-defined thresholds will be outlined in the SAP and made without reference to the effectiveness data.

No formal interim analyses were planned. Blinded interim reports were presented to the TSC containing descriptive information. This included data on recruitment, follow-up, adherence, safety and data quality. These were presented by randomised arm if specifically requested as such, with treatments masked. As participants were recruited following cluster randomisation, monitoring of baseline characteristics by arm are presented to assess post-randomisation selection bias. A single final analysis is planned when all follow-up data has been entered onto trial database, and the database is cleaned and locked.

In the cost-effectiveness analysis, the primary outcome is Quality Adjusted Life Years (QALYs) compared to costs from an NHS and PSS perspective. A secondary cost effectiveness analysis will look at the incremental cost per all-cause deaths prevented and re-hospitalisation prevented. Resource use associated with 'treatment as usual' and the intervention will be collected through the health resource use questionnaire administered to patients. Prescription and hospitalisation data will be collected from the data linkage outputs and supported where necessary by the patient health resource use questionnaire at 3 & 12 months. Unit costs for health service resources will be obtained from national sources (e.g. Personal Social Services Research Unit [PSSRU] British National Formulary for medicines). The intervention cost will include any enhancement of existing medicines management components and any 'new' components of the MaTI (e. g. the staff training module or necessary enhancements to existing community pharmacy medicines services). We will assess uncertainty using a within trial probabilistic sensitivity analysis undertaken using Monte Carlo simulation with results presented as Incremental Cost-Effectiveness rations (ICERs) and cost effectiveness acceptability curves.

For further detail on outcome measures, see Table 4.

*Table 4: Data analysis plan for primary and secondary outcomes and health economic analysis*

| Analysis activities | Assessed At | Statistical methods |
| --- | --- | --- |


For peer review only - http://bmjopen.bmj.com/site/about/guidelines.xhtml

| | | |
|---|---|---|
| **Primary Endpoint – all-cause mortality and heart failure-related rehospitalisation within 12 months post-registration** | 12 months post registration | The primary endpoint will be analysed using a two-level logistic regression model with participants nested within clusters, with clusters treated as random effects. The model will be adjusted for the following fixed effects: the cluster level stratification variables (level 2): geographical region, type of hospital and method of clinical handover/information transfer to community pharmacy, and patient level covariates (level 1) and other relevant known predictors of outcome. Results will be expressed as point estimates, with corresponding 95% CIs, p-values. An estimate of the ICC and corresponding CI will also be presented. |
| **Key secondary endpoint** | 12 months post-registration | This endpoint will be analysed using a similar modelling strategy as the primary endpoint. |
| **Other secondary endpoints** | 12 months post-registration | Time-to-event analyses will be by multi-level shared frailty models with the event right-censored at either 12 months follow-up, date of death or date of withdrawal, whichever is earliest.<br><br>Days alive and out of hospital will be analysed using mixed effects Poisson regression model.[29] with data censored at either 12-months post-registration, death or withdrawal, whichever is earliest.<br><br>Length of time on guideline recommended cardiovascular medications and cause specific death endpoints will be summarized descriptively. |
| | 2 & 6 weeks post-discharge; 12 months post-registration | Patient understanding of their medicines and satisfaction with medicines-related care will be analysed using Ordinal logistic regression with clustering model to compare participant responses between treatment arms. |
| **Cost-effectiveness analysis** | 12 months post-registration | The primary outcome will be Quality Adjusted Life Years (QALYs). A secondary cost effectiveness analysis will look at the incremental cost per all-cause deaths prevented and re-hospitalisation prevented.<br><br>In order to assess the long-term cost-effectiveness of the intervention, compared to treatment as usual, a decision analytic cost-effectiveness model will be used to estimate the expected incremental cost per QALY. The model will include a Value of Information Analysis (VOIA) to assess the value of undertaking further research to reduce decision uncertainty in the model. The perspective will be the same as the within-trial analysis but the time horizon will be the lifetime of the individual to capture the full impact of any mortality differences on the long-term cost-effectiveness. |


For peer review only - http://bmjopen.bmj.com/site/about/guidelines.xhtml

| | | |
|---|---|---|
| | | Estimation of health-related quality of life will use QALYs and discounting will be at the same rate as for the within trial analysis. Parameter uncertainty will be addressed using probabilistic sensitivity analysis and running Monte Carlo simulations. Results will meet international CHEERS standards of reporting and results will be presented as Incremental Cost-Effectiveness rations (ICERs), Incremental Net Health and Monetary Benefits and Cost Effectiveness Acceptability Curves (CEACs). |

**Data monitoring**

Data is monitored for quality and completeness by the Clinical Trials Research Unit (CTRU) using established verification, validation and checking processes. The Trial Management Group (TMG) comprising the chief investigator (CI), CTRU team, other co-applicants and PPI representative will be responsible for the on-going management of the trial. The TMG is responsible for any audits for trial conduct required. The TMG reports to the Trial Steering Committee (TSC) annually. The TSC is formed with an independent chair, health economist and statisticians.

**Methods to handle missing data**

Missing data are expected and the proportion of missing data will be compared between intervention and control groups. Sensitivity analyses of the primary endpoint will be conducted to assess the impact of missing data, the choice of imputation model and assumptions around the missingness mechanism, as appropriate.

**Ethics and dissemination**

The trial received favourable REC opinion from Yorkshire and the Humber – Bradford Leeds Health Research Ethics Committee on 30/01/2018 with reference number 18/YH/0017
The trial was registered (ISRCTN66212970).

The trial is sponsored by Bradford Teaching Hospitals NHS Foundation Trust, coordinated by the CTRU at the University of Leeds and supported by a TMG and Programme Management Group (PMG) TSC. The TSC includes independent external experts who provide overall supervision for a trial on behalf of the Trial Sponsor and Trial Funder.

This longstanding and ongoing engagement with stakeholders provides a direct pathway to impact for the outputs of this research. Our Patient-Led Steering Group (PLSG) will inform our dissemination strategy and its members will play an active role in the format and content of academic papers


For peer review only - http://bmjopen.bmj.com/site/about/guidelines.xhtml
For peer review only

(specifically patient implications) and will present at local, regional and national conferences and meetings.

**Patient and public involvement**

ISCOMAT has a Patient-Led Steering Group that has contributed throughout the programme. Patient representatives have membership of the TMG, Programme Management Group and TSC. The PLSG has played an integral role in several aspects of the study, including intervention development, the development of data collection tools and the analysis of qualitative interview data. Members of the group have also served as co-authors in programme and trial publications.

**COVID-19 pandemic and considerations for trial closure**

Sites had been brought on gradually in waves across a two-year site set-up period. Sites also closed at various points throughout this period due to reaching or exceeding site-specific targets sample sizes or due to changes in capacity to deliver the intervention. Prior to the onset of the COVID-19 pandemic, we had needed to extend recruitment for a minimum of one year in order to try and achieve target sample size. A full report on the recruitment rate and generalizability of results will be available in the trial results paper that will be published on completion of trial analysis.

The COVID-19 adversely affected routine NHS services, and there was a decline in admissions with cardiovascular disease.[30 31] Following national NIHR guidance, sponsor decision and TSC review, the ISCOMAT trial was paused in March 2020 and formally closed to recruitment in July 2020. Uncertainty about the post-COVID landscape raised concerns for intervention relevance and deliverability, future research capacity and participant willingness to be involved in research, number of admissions and organisation of heart failure services in sites, plus external policy changes, such as medicines use reviews (MURs). Additional concerns included the effect of site withdrawals following a restart on the trial design and increased post-COVID death rates on our findings. The decision to close the trial was supported by statistical review that concluded that closing recruitment would not have a significant impact on the statistical power or analysis of the primary and key secondary outcomes, given on the proportion of the target sample size recruited to date. Furthermore, this design maximizing routine data sources helped to minimise the impact of COVID-19 on trial analysis plans.


For peer review only - http://bmjopen.bmj.com/site/about/guidelines.xhtml

**Acknowledgements**

We would like to thank Alison Blenkinsopp, Gerry Armitage, Theo Rayner, Ian Kellar, Jan Speechley, the ISCOMAT Patient-led Steering Group, the ISCOMAT Trial Management Group, Trial Steering Committee and Programme Steering Committee.

**Author contributions**

IH, BF, SH, BC, AF, SA, DA, CB, LB, HI, EM, PG, CP, JS, AT, AJF, CPG developed the methodology and detail of the ISCOMAT trial protocol. LM drafted the manuscript, with all authors reviewing the draft critically for intellectual content. All authors approved the final version submitted for publication.

**Funding Statement**

The study was funded as part of a National Institute for Health Research Programme Grant for Applied Research (RP-PG-0514-20009). The views expressed in this paper are those of the authors and not necessarily those of the NHS, the NIHR or the Department of Health.

**Trial Sponsor**

The trial is sponsored by Bradford Teaching Hospitals NHS Foundation Trust.

**Data statement**

All data requests should be submitted to the corresponding author for consideration. Access to anonymised data may be granted following review.

**Disclaimer**

The views expressed are those of the authors and not necessarily those of the NIHR.

**Competing interests Statement.**

None declared.

For peer review only

## References


1. Ambrosy AP, Fonarow GC, Butler J, et al. The Global Health and Economic Burden of Hospitalizations for Heart Failure: Lessons Learned From Hospitalized Heart Failure Registries. *Journal of the American College of Cardiology* 2014;63(12):1123-33. doi: https://doi.org/10.1016/j.jacc.2013.11.053
2. Conrad N, Judge A, Tran J, et al. Temporal trends and patterns in heart failure incidence: a population-based study of 4 million individuals. *The Lancet* 2018;391(10120):572-80. doi: 10.1016/S0140-6736(17)32520-5
3. Kim D, Hayhoe B, Aylin P, et al. Health service use by patients with heart failure living in a community setting: a cross-sectional analysis in North West London. *British Journal of General Practice* 2020;70(697):e563. doi: 10.3399/bjgp20X711749
4. Shafie AA, Tan YP, Ng CH. Systematic review of economic burden of heart failure. *Heart Failure Reviews* 2018;23(1):131-45. doi: 10.1007/s10741-017-9661-0
5. Ponikowski P, Voors AA, Anker SD, et al. 2016 ESC Guidelines for the diagnosis and treatment of acute and chronic heart failure: The Task Force for the diagnosis and treatment of acute and chronic heart failure of the European Society of Cardiology (ESC)Developed with the special contribution of the Heart Failure Association (HFA) of the ESC. *European Heart Journal* 2016;37(27):2129-200. doi: 10.1093/eurheartj/ehw128
6. McMurray JJV, Adamopoulos S, Anker SD, et al. ESC Guidelines for the diagnosis and treatment of acute and chronic heart failure 2012 The Task Force for the Diagnosis and Treatment of Acute and Chronic Heart Failure 2012 of the European Society of Cardiology. Developed in collaboration with the Heart Failure Association (HFA) of the ESC. *Eur J Heart Fail* 2012;14(8):803-69. doi: 10.1093/eurjhf/hfs105
7. Fylan B, Marques I, Ismail H, et al. Gaps, traps, bridges and props: a mixed-methods study of resilience in the medicines management system for patients with heart failure at hospital discharge. *Bmj Open* 2019;9(2) doi: ARTN e023440

10.1136/bmjopen-2018-023440

8. (WHO) WHO. Medication safety in transition of care: World Health Organisation; 2019 [Available from: https://www.who.int/publications/i/item/WHO-UHC-SDS-2019.9 accessed 16/02 2022.
9. Simms AD, Baxter PD, Cattle BA, et al. An assessment of composite measures of hospital performance and associated mortality for patients with acute myocardial infarction. Analysis of individual hospital performance and outcome for the National Institute for Cardiovascular Outcomes Research (NICOR). *European Heart Journal: Acute Cardiovascular Care* 2013;2(1):9-18.
10. Raynor DK, Ismail H, Blenkinsopp A, et al. Experience-based co-design-Adapting the method for a researcher-initiated study in a multi-site setting. *Health Expect* 2020 doi: 10.1111/hex.13028 [published Online First: 2020/02/12]
11. Fylan B, Ismail H, Hartley S, et al. A non-randomised feasibility study of an intervention to optimise medicines at transitions of care for patients with heart failure. *Pilot and Feasibility Studies* 2021;7(1):85. doi: 10.1186/s40814-021-00819-x
12. Council MR. Developing and evaluating complex interventions [Available from: https://mrc.ukri.org/documents/pdf/complex-interventions-guidance/.
13. National Institute for Health & Care Excellence (NICE). Nice guideline [NG106}. Chronic heart failure in adults: diagnosis and management 2018 [Available from: https://www.nice.org.uk/guidance/ng106.



For peer review only - http://bmjopen.bmj.com/site/about/guidelines.xhtml

14. Powell C, Breen L, Fylan B, et al. Improving the Safety and Continuity Of Medicines management at Transitions of care (ISCOMAT): protocol for a process evaluation of a cluster randomised control trial. 2020;10(11):e040493. doi: 10.1136/bmjopen-2020-040493 %J BMJ Open
15. National Institute for Health Research. Clinical Research Network [Available from: https://www.nihr.ac.uk/explore-nihr/support/clinical-research-network.htm accessed 18/01/2021 2021.
16. AUKUH Member Organisations. https://www.universityhospitals.org.uk/ [
17. Pharmoutcomes [program].
18. Puffer S, Torgerson D, Watson J. Evidence for risk of bias in cluster randomised trials: review of recent trials published in three general medical journals. *BMJ* 2003;327(7418):785-9. doi: 10.1136/bmj.327.7418.785 [published Online First: 2003/10/04]
19. Gale NK, Heath G, Cameron E, et al. Using the framework method for the analysis of qualitative data in multi-disciplinary health research. *BMC medical research methodology* 2013;13(1):117.
20. Damschroder LJ, Aron DC, Keith RE, et al. Fostering implementation of health services research findings into practice: a consolidated framework for advancing implementation science. *Implementation science* 2009;4(1):50.
21. NHS Digital. Hospital Episode Statistics (HES) Data [Available from: https://digital.nhs.uk/data-and-information/data-tools-and-services/data-services/hospital-episode-statistics accessed 18/01/2021 2021.
22. Williams A. Euroqol - a New Facility for the Measurement of Health-Related Quality-of-Life. *Health Policy* 1990;16(3):199-208.
23. Data Protection Act 2018, 2018.
24. Shah RV, McNulty S, O'connor CM, et al. Effect of admission oral diuretic dose on response to continuous versus bolus intravenous diuretics in acute heart failure: an analysis from diuretic optimization strategies in acute heart failure. *American heart journal* 2012;164(6):862-68.
25. Palazzuoli A, Pellegrini M, Ruocco G, et al. Continuous versus bolus intermittent loop diuretic infusion in acutely decompensated heart failure: a prospective randomized trial. *Critical care* 2014;18(3):R134.
26. McMurray J, Packer M, Desai A, et al. PARADIGM-HF Investigators and Committees. *Angiotensin-neprilysin inhibition versus enalapril in heart failure N Engl J Med* 2014;371(11):993-1004.
27. Campbell MK, Piaggio G, Elbourne DR, et al. Consort 2010 statement: extension to cluster randomised trials. *Bmj* 2012;345:e5661. doi: 10.1136/bmj.e5661 [published Online First: 2012/09/07]
28. Kwakkenbos L, Imran M, McCall SJ, et al. CONSORT extension for the reporting of randomised controlled trials conducted using cohorts and routinely collected data (CONSORT-ROUTINE): checklist with explanation and elaboration. 2021;373:n857. doi: 10.1136/bmj.n857 %J BMJ
29. Fanaroff AC, Cyr D, Neely ML, et al. Days Alive and Out of Hospital: Exploring a Patient-Centered, Pragmatic Outcome in a Clinical Trial of Patients With Acute Coronary Syndromes. *Circ Cardiovasc Qual Outcomes* 2018;11(12):e004755. doi: 10.1161/CIRCOUTCOMES.118.004755 [published Online First: 2018/12/19]
30. Mafham MM, Spata E, Goldacre R, et al. COVID-19 pandemic and admission rates for and management of acute coronary syndromes in England. *Lancet (London, England)* 2020;396(10248):381-89. doi: 10.1016/s0140-6736(20)31356-8 [published Online First: 2020/07/18]

For peer review only



For peer review only - http://bmjopen.bmj.com/site/about/guidelines.xhtml

31. Shoaib A, Van Spall HGC, Wu J, et al. Substantial decline in hospital admissions for heart failure accompanied by increased community mortality during COVID-19 pandemic. *European Heart Journal - Quality of Care and Clinical Outcomes* 2021 doi: 10.1093/ehjqcco/qcab040

For peer review only



For peer review only - http://bmjopen.bmj.com/site/about/guidelines.xhtml

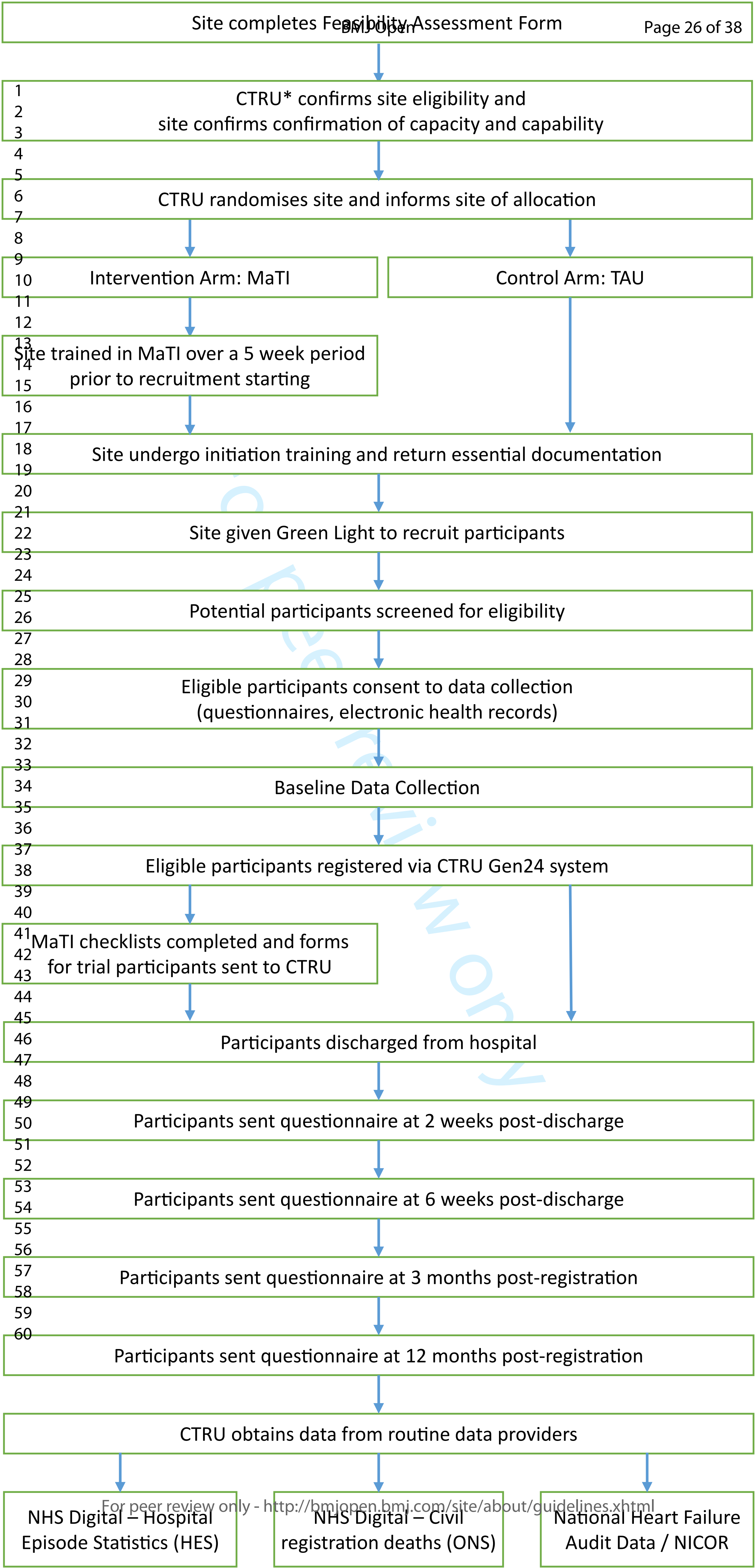
Site completes Feasibility Assessment Form
CTRU* confirms site eligibility and site confirms confirmation of capacity and capability
CTRU randomises site and informs site of allocation
Intervention Arm: MaTI
Control Arm: TAU
Site trained in MaTI over a 5 week period prior to recruitment starting
Site undergo initiation training and return essential documentation
Site given Green Light to recruit participants
Potential participants screened for eligibility
Eligible participants consent to data collection (questionnaires, electronic health records)
Baseline Data Collection
Eligible participants registered via CTRU Gen24 system
MaTI checklists completed and forms for trial participants sent to CTRU
Participants discharged from hospital
Participants sent questionnaire at 2 weeks post-discharge
Participants sent questionnaire at 6 weeks post-discharge
Participants sent questionnaire at 3 months post-registration
Participants sent questionnaire at 12 months post-registration
CTRU obtains data from routine data providers
NHS Digital – Hospital Episode Statistics (HES)
NHS Digital – Civil registration deaths (ONS)
National Heart Failure Audit Data / NICOR

## MaTI Form A

**Checklist for MaTI patients**

Keep this checklist in the MaTI box file in the agreed place on the ward

Patient name: ______________________________

DoB: ________________ NHS number: ________________

## MaTI Form A

**Checklist for MaTI patients**

*FOR STUDY USE ONLY*

INITIALS ______ DOB ______ TRIAL No. ______

SITE NAME ______ SITE No. ______

| Step | Description and tool used | When? | Date completed | Who? (role, initials) | Unable to complete (reason) |
|---|---|---|---|---|---|
| 1 | MaTI checklist in patient's notes | During admission | | | |
| 2a | Identify community pharmacist (Pharmacy form to include in notes) | During admission/ medicines reconciliation | | | |
| 2b | Contact the pharmacist to let them about the patient and discharge information | | | | |
| 3 | Patient introduced to the My Medicines Toolkit booklet (MaTI booklet) | Before the day of discharge | | | |
| 4 | Discharge heart failure medicines log completed (foldout in MaTI toolkit) | On day of discharge | | | |
| 5 | Patient informed about being referred to their community pharmacy for a follow up and a medicines use review / discussion about medicines | On day of discharge | | | |
| 6 | Transfer the patient's discharge advice letter and medicines list to the community pharmacy (MaTI community pharmacy cover letter) | At / soon after discharge | | | |
| 7 | Information has been received by the community pharmacist? (by telephone) | At / soon after discharge | Y/ N | | |

## MaTI Form B
**Community pharmacy record**

Date: ______________________________

Patient name: ______________________________

DoB: ______________________________

---

## MaTI Form B
**Community pharmacy record**

| *FOR STUDY USE ONLY* | INITIALS | | DOB | | TRIAL No. | |
|---|---|---|---|---|---|---|
| | SITE NAME | | | | SITE No. | |

Date: ______________________________

Community pharmacy name: ______________________________

Community pharmacy address: ______________________________

______________________________

______________________________

______________________________

Phone: ______________________________

Fax: ______________________________

Secure NHS email: ______________________________

# Community Pharmacy Cover Letter

**Ward Contact Details** Name ______________________ Telephone ______________________

**Handover of patient medicines care to community pharmacy**

Patient name: ______________________________________________

D.O.B: ______________________________________________

To the Pharmacist in charge

**A patient has now been discharged from hospital and action is required by you**

## What is this about?

We are writing to you because we have introduced improved discharge communication between our hospital and community pharmacy for patients with heart failure. It is part of a study called ISCOMAT/MaTI. For more information please see **https://www.brad.ac.uk/life-sciences/pharmacy-medical-sciences/morg/iscomat/**

## What do I need to do?

We have attached a copy of the patient's discharge advice note / letter and this includes a copy of the patient's discharge medicines list. You now need to:

- Reconcile the patient's medicines (resolve any discrepancies between the medicines in the discharge list and the patient's next repeat prescription).
- Invite the patient for a post-discharge Medicines Use Review (MUR), or a discussion about their medicines (to answer any questions they may have about their medicines, to resolve any discrepancies and to provide support as necessary).
- Complete the checklist included and keep it with your records.

If the patient wishes their MUR to take place by telephone you can apply for permission to do this using the form on the PSNC website:
**http://psnc.org.uk/services-commissioning/advanced-services/murs/conducting-murs-off-the-pharmacy-premises/**

## Free online CPD for you and your staff

If you and/or a member of your team would like to you can complete a short e-learning CPD module for staff involved with patients' medicines - to help them find out more about the risks that patients face when their care is transferred between providers. This training, which will take approximately one hour, is available free of charge to you and your team. We hope you will encourage your staff to undertake it. It has been developed by the Centre for Pharmacy Postgraduate Education (CPPE). It can be accessed here:
**www.cppe.ac.uk/canvas/iscomat**

ISCOMAT is a national study and has Health Research Authority (HRA) and Research Ethics Committee approval.

**If you require any further information about the patient, please contact staff on the ward using the contact details at the top of this letter.**

# Reporting checklist for protocol of a clinical trial.

Based on the SPIRIT guidelines.

## Instructions to authors

Complete this checklist by entering the page numbers from your manuscript where readers will find each of the items listed below.

Your article may not currently address all the items on the checklist. Please modify your text to include the missing information. If you are certain that an item does not apply, please write "n/a" and provide a short explanation.

Upload your completed checklist as an extra file when you submit to a journal.

In your methods section, say that you used the SPIRITreporting guidelines, and cite them as:

Chan A-W, Tetzlaff JM, Gøtzsche PC, Altman DG, Mann H, Berlin J, Dickersin K, Hróbjartsson A, Schulz KF, Parulekar WR, Krleža-Jerić K, Laupacis A, Moher D. SPIRIT 2013 Explanation and Elaboration: Guidance for protocols of clinical trials. BMJ. 2013;346:e7586

| | | Reporting Item | Page Number |
|---|---|---|---|
| **Administrative information** | | | |
| Title | #1 | Descriptive title identifying the study design, population, interventions, and, if applicable, trial acronym | 1 |
| Trial registration | #2a | Trial identifier and registry name. If not yet registered, | 3 |

For peer review only

| | | | |
|---|---|---|---|
| | | name of intended registry | |
| Trial registration: data set | #2b | All items from the World Health Organization Trial Registration Data Set | N/A |
| Protocol version | #3 | Date and version identifier | 6 |
| Funding | #4 | Sources and types of financial, material, and other support | 22 |
| Roles and responsibilities: contributorship | #5a | Names, affiliations, and roles of protocol contributors | 1-2; 21-2 |
| Roles and responsibilities: sponsor contact information | #5b | Name and contact information for the trial sponsor | 22 |
| Roles and responsibilities: sponsor and funder | #5c | Role of study sponsor and funders, if any, in study design; collection, management, analysis, and interpretation of data; writing of the report; and the decision to submit the report for publication, including whether they will have ultimate authority over any of these activities | 22 |
| Roles and responsibilities: committees | #5d | Composition, roles, and responsibilities of the coordinating centre, steering committee, endpoint adjudication committee, data management team, and other individuals or groups overseeing the trial, if applicable (see Item 21a for data monitoring committee) | 20 |

**Introduction**

For peer review only

| | | | |
|---|---|---|---|
| Background and rationale | #6a | Description of research question and justification for undertaking the trial, including summary of relevant studies (published and unpublished) examining benefits and harms for each intervention | 5 |
| Background and rationale: choice of comparators | #6b | Explanation for choice of comparators | 11 |
| Objectives | #7 | Specific objectives or hypotheses | 5 |
| Trial design | #8 | Description of trial design including type of trial (eg, parallel group, crossover, factorial, single group), allocation ratio, and framework (eg, superiority, equivalence, non-inferiority, exploratory) | |
| **Methods: Participants, interventions, and outcomes** | | | |
| Study setting | #9 | Description of study settings (eg, community clinic, academic hospital) and list of countries where data will be collected. Reference to where list of study sites can be obtained | 9 |
| Eligibility criteria | #10 | Inclusion and exclusion criteria for participants. If applicable, eligibility criteria for study centres and individuals who will perform the interventions (eg, | 10 |

For peer review only

| | | | |
|---|---|---|---|
| | | surgeons, psychotherapists) | |
| Interventions: description | #11a | Interventions for each group with sufficient detail to allow replication, including how and when they will be administered | 11 |
| Interventions: modifications | #11b | Criteria for discontinuing or modifying allocated interventions for a given trial participant (eg, drug dose change in response to harms, participant request, or improving / worsening disease) | N/A |
| Interventions: adherance | #11c | Strategies to improve adherence to intervention protocols, and any procedures for monitoring adherence (eg, drug tablet return; laboratory tests) | 12 |
| Interventions: concomitant care | #11d | Relevant concomitant care and interventions that are permitted or prohibited during the trial | N/A |
| Outcomes | #12 | Primary, secondary, and other outcomes, including the specific measurement variable (eg, systolic blood pressure), analysis metric (eg, change from baseline, final value, time to event), method of aggregation (eg, median, proportion), and time point for each outcome. Explanation of the clinical relevance of chosen efficacy and harm outcomes is strongly recommended | 5, 18 |
| Participant timeline | #13 | Time schedule of enrolment, interventions (including any run-ins and washouts), assessments, and visits for participants. A schematic diagram is highly recommended (see Figure) | 8 |

For peer review only

| | | | |
|---|---|---|---|
| Sample size | #14 | Estimated number of participants needed to achieve study objectives and how it was determined, including clinical and statistical assumptions supporting any sample size calculations | 6 |
| Recruitment | #15 | Strategies for achieving adequate participant enrolment to reach target sample size | |
| **Methods: Assignment of interventions (for controlled trials)** | | | |
| Allocation: sequence generation | #16a | Method of generating the allocation sequence (eg, computer-generated random numbers), and list of any factors for stratification. To reduce predictability of a random sequence, details of any planned restriction (eg, blocking) should be provided in a separate document that is unavailable to those who enrol participants or assign interventions | 9 |
| Allocation concealment mechanism | #16b | Mechanism of implementing the allocation sequence (eg, central telephone; sequentially numbered, opaque, sealed envelopes), describing any steps to conceal the sequence until interventions are assigned | 9 |
| Allocation: implementation | #16c | Who will generate the allocation sequence, who will enrol participants, and who will assign participants to interventions | 9 |

For peer review only

| | | | |
|---|---|---|---|
| Blinding (masking) | #17a | Who will be blinded after assignment to interventions (eg, trial participants, care providers, outcome assessors, data analysts), and how | 9-10 |
| Blinding (masking): emergency unblinding | #17b | If blinded, circumstances under which unblinding is permissible, and procedure for revealing a participant's allocated intervention during the trial | 9-10 |
| **Methods: Data collection, management, and analysis** | | | |
| Data collection plan | #18a | Plans for assessment and collection of outcome, baseline, and other trial data, including any related processes to promote data quality (eg, duplicate measurements, training of assessors) and a description of study instruments (eg, questionnaires, laboratory tests) along with their reliability and validity, if known. Reference to where data collection forms can be found, if not in the protocol | 15 |
| Data collection plan: retention | #18b | Plans to promote participant retention and complete follow-up, including list of any outcome data to be collected for participants who discontinue or deviate from intervention protocols | 15 |
| Data management | #19 | Plans for data entry, coding, security, and storage, including any related processes to promote data quality (eg, double data entry; range checks for data values). | 20 |

For peer review only

| | | | |
|---|---|---|---|
| | | Reference to where details of data management procedures can be found, if not in the protocol | |
| Statistics: outcomes | #20a | Statistical methods for analysing primary and secondary outcomes. Reference to where other details of the statistical analysis plan can be found, if not in the protocol | 17 |
| Statistics: additional analyses | #20b | Methods for any additional analyses (eg, subgroup and adjusted analyses) | 17 |
| Statistics: analysis population and missing data | #20c | Definition of analysis population relating to protocol non-adherence (eg, as randomised analysis), and any statistical methods to handle missing data (eg, multiple imputation) | 17, 20 |
| **Methods: Monitoring** | | | |
| Data monitoring: formal committee | #21a | Composition of data monitoring committee (DMC); summary of its role and reporting structure; statement of whether it is independent from the sponsor and competing interests; and reference to where further details about its charter can be found, if not in the protocol. Alternatively, an explanation of why a DMC is not needed | N/A |
| Data monitoring: interim analysis | #21b | Description of any interim analyses and stopping guidelines, including who will have access to these interim results and make the final decision to terminate the trial | 18 |
| Harms | #22 | Plans for collecting, assessing, reporting, and managing solicited and spontaneously reported adverse events and other unintended effects of trial interventions or trial | 14 |

For peer review only

| | | | |
|---|---|---|---|
| | | conduct | |
| Auditing | #23 | Frequency and procedures for auditing trial conduct, if any, and whether the process will be independent from investigators and the sponsor | 20 |
| **Ethics and dissemination** | | | |
| Research ethics approval | #24 | Plans for seeking research ethics committee / institutional review board (REC / IRB) approval | 4, 20 |
| Protocol amendments | #25 | Plans for communicating important protocol modifications (eg, changes to eligibility criteria, outcomes, analyses) to relevant parties (eg, investigators, REC / IRBs, trial participants, trial registries, journals, regulators) | 4 |
| Consent or assent | #26a | Who will obtain informed consent or assent from potential trial participants or authorised surrogates, and how (see Item 32) | 9 |
| Consent or assent: ancillary studies | #26b | Additional consent provisions for collection and use of participant data and biological specimens in ancillary studies, if applicable | N/A |
| Confidentiality | #27 | How personal information about potential and enrolled participants will be collected, shared, and maintained in order to protect confidentiality before, during, and after the trial | 14 |
| Declaration of | #28 | Financial and other competing interests for principal | 22 |

For peer review only

| | | | |
|---|---|---|---|
| interests | | investigators for the overall trial and each study site | |
| Data access | #29 | Statement of who will have access to the final trial dataset, and disclosure of contractual agreements that limit such access for investigators | 22 |
| Ancillary and post trial care | #30 | Provisions, if any, for ancillary and post-trial care, and for compensation to those who suffer harm from trial participation | N/A |
| Dissemination policy: trial results | #31a | Plans for investigators and sponsor to communicate trial results to participants, healthcare professionals, the public, and other relevant groups (eg, via publication, reporting in results databases, or other data sharing arrangements), including any publication restrictions | 4 |
| Dissemination policy: authorship | #31b | Authorship eligibility guidelines and any intended use of professional writers | 4 |
| Dissemination policy: reproducible research | #31c | Plans, if any, for granting public access to the full protocol, participant-level dataset, and statistical code | 22 |
| **Appendices** | | | |
| Informed consent materials | #32 | Model consent form and other related documentation given to participants and authorised surrogates | |
| Biological specimens | #33 | Plans for collection, laboratory evaluation, and storage of biological specimens for genetic or molecular analysis in the current trial and for future use in ancillary studies, if applicable | N/A |

For peer review only

None The SPIRIT Explanation and Elaboration paper is distributed under the terms of the Creative Commons Attribution License CC-BY-NC. This checklist can be completed online using https://www.goodreports.org/, a tool made by the EQUATOR Network in collaboration with Penelope.ai

For peer review only
